# Genomic alterations associated with mutational signatures, DNA damage repair and chromatin remodeling pathways in cervical carcinoma

**DOI:** 10.1038/s41525-021-00244-2

**Published:** 2021-10-07

**Authors:** Mari K. Halle, Aishwarya Sundaresan, Jianqing Zhang, Chandra Sekhar Pedamallu, Vinodh Srinivasasainagendra, Jessica Blair, Dewey Brooke, Bjørn I. Bertelsen, Kathrine Woie, Sadeep Shrestha, Hemant Tiwari, Yick Fu Wong, Camilla Krakstad, Akinyemi I. Ojesina

**Affiliations:** 1 Department of Obstetrics and Gynaecology, Haukeland University Hospital, Bergen, Norway; 2 Centre for Cancer Biomarkers, Department of Clinical Science, University of Bergen, Bergen, Norway; 3 Department of Epidemiology, University of Alabama at Birmingham, Birmingham, AL USA; 4 Department of Biostatistics, University of Alabama at Birmingham, Birmingham, AL USA; 5 Department of Pathology, Haukeland University Hospital, Bergen, Norway; 6 Department of Obstetrics and Gynecology, The Chinese University of Hong Kong, Prince of Wales Hospital, Shatin, Hong Kong; 7 O’Neal Comprehensive Cancer Center, University of Alabama at Birmingham, Birmingham, AL USA; 8 HudsonAlpha Institute for Biotechnology, Huntsville, AL USA

**Keywords:** Prognostic markers, Genome assembly algorithms, Predictive markers, Cancer genomics, Cancer

## Abstract

Despite recent advances in the prevention of cervical cancer, the disease remains a leading cause of cancer-related deaths in women worldwide. By applying the GISTIC2.0 and/or the MutSig2CV algorithms on 430 whole-exome-sequenced cervical carcinomas, we identified previously unreported significantly mutated genes (SMGs) (including *MSN*, *GPX1*, *SPRED3, FAS*, and *KRT8*), amplifications (including *NFIA, GNL1*, *TGIF1*, and *WDR87*) and deletions (including *MIR562, PVRL1*, and *NTM*). Subset analyses of 327 squamous cell carcinomas and 86 non-squamous cell carcinomas revealed previously unreported SMGs in *BAP1* and *IL28A*, respectively. Distinctive copy number alterations related to tumors predominantly enriched for *CpG- and Tp*C mutations were observed. *CD274*, *GRB2*, *KRAS*, and *EGFR* were uniquely significantly amplified within the Tp*C-enriched tumors. A high frequency of aberrations within DNA damage repair and chromatin remodeling genes were detected. Facilitated by the large sample size derived from combining multiple datasets, this study reveals potential targets and prognostic markers for cervical cancer.

## Introduction

Cervical cancer (CC) is the fourth leading cause of cancer in women and is responsible for more than 340,000 annual deaths^1^. Although the recently revised Federation Internationale de Gynècologie et d’Obstètrique (FIGO) classification system^2^ has improved risk stratification, patients with advanced disease stages have few targeted treatment options and poor survival. In terms of histology, cervical cancer is a diverse disease. Squamous cell carcinomas and adenocarcinomas comprise the majority with ~70% and 22% of diagnosed cases, respectively^3^. The less abundant adenosquamous (3%), neuroendocrine (1%), and undifferentiated (1%) carry a particularly poor prognosis, yet due to their marginal prevalence, they are also less studied^3,4^. There is a need to identify somatic drivers, particularly within these less abundant histologies to facilitate the development of new therapeutic strategies for cervical cancer patients.

Recent large-scale genomic sequencing studies have uncovered distinctive molecular features characterizing invasive cervical cancer such as the APOBEC mutational signature^5,6^. Distinctive mutational signatures may point to definite underlying mutational processes^7^. Correspondingly, tumors with distinct mutational signatures may be associated with specific genomic alterations, which complement the underlying processes. However, the impact of different mutational signatures on molecular subtypes and potential treatment strategies in cervical cancer have so far not been investigated. In addition, the multiplatform nature of large-scale projects like the Cancer Genome Atlas (TCGA) is associated with tradeoffs of analytical breadth versus depth on the data from any single platform. We have performed a comprehensive analysis of somatic alterations (mutations and copy number) by combining publicly available data (including but not limited to TCGA) and report distinctive genomic features associated with major mutational profiles and other unique subsets associated with cervical carcinoma. Furthermore, we present previously unreported functionally altered signaling pathways that may drive carcinogenesis and serve as prognostic and/or therapeutic markers.

## Results

### Somatic mutations

All samples had at least 28 Mb of the whole exome covered. Mutect2.0 analyses revealed a total of 111,892 somatic mutations, including 70,060 missense, 6,044 nonsense, 29,491 silent, 2494 splice site mutations, as well as 2400 deletions, 857 insertions, 399 translation start site, and 147 nonstop mutations. The median nonsilent mutation rate was 3.1 per Mb. Squamous cell carcinomas (SCCs) had significantly higher nonsilent mutation rate per Mb (*n* = 327, median 2.84), when compared to adenocarcinomas (*n* = 88, median 1.77) (*P* < 0.001, Supplementary Fig. 1). Adenosquamous (*n* = 13) and neuroendocrine (*n* = 2) tumors had a median nonsilent mutation rate of 1.84 and 2.05, respectively, yet patient numbers were too small to gain statistical significance (Supplementary Fig. 1). The clinicopathological, epidemiological, and mutational characteristics of all included cases are provided in Supplementary Tables 1 and 2.

The most frequent significantly mutated genes (SMGs) across the 430 cervical carcinomas were previously reported^5,6,8–11^ and included *PIK3CA (*27%)*, KMT2C (*also known as *MLL3*) (19%), *KMT2D (*also known as *MLL2*) *(*13%)*, EP300 (*12%)*, FBXW7* (12%), *FAT1* (8%), and *PTEN* (8%) (Supplementary Table 3). The increased sample size enabled identification of SMGs previously unreported in cervical carcinoma including *NBPF10* (8%), *RANBP2* (6%), *MSN* (6%), *KRT8* (6%), *POTEC* (6%), *ZC3H11A* (4%), *BMS1* (4%), *GPX1* (3%), *OTOP*1 (3%), *DNAH12* (2%), *COIL* (2%), *TBC1D26* (2%), *SPRED3* (2%), *FAM115A* (2%), and *ARIH1* (1%) (Table 1). Interestingly, nonsilent mutations within *GPX1* associated significantly with poor survival (*P* = 0.01, Supplementary Fig. 2). Associations to overall survival for all the previously unreported SMGs are summarized in Supplementary Fig. 2.

**Table 1:** Novel significantly mutated genes not previously reported on in cervical carcinoma.

Gene	Nonsilent mutations	Relative frequency	Patients	Unique sites	Missense mutation	Silent mutations	“LOF” mutations^a^	FDR
*KRT8*	26	6%	25	3	26	1	0	6.10E-06
*GPX1*	12	3%	11	3	11	1	1	2.57E-04
*ZC3H11A*	20	4%	19	9	10	3	10	3.73E-04
*OTOP1*	11	3%	11	5	4	0	7	9.88E-03
*ARIH1*	3	1%	3	1	0	0	3	1.05E-02
*COIL*	10	2%	9	7	5	0	5	1.05E-02
*TBC1D26*	9	2%	8	3	9	1	0	1.68E-02
*RANBP2*	29	6%	26	23	24	5	5	2.15E-02
*POTEC*	26	6%	24	13	23	3	3	2.52E-02
*SPRED3*	7	2%	7	1	0	0	7	3.45E-02
*BMS1*	15	3%	15	10	14	4	1	5.29E-02
*DNAH12*	10	2%	10	9	5	2	5	5.49E-02
*MSN*	26	6%	26	8	25	16	1	5.49E-02
*NBPF10*	35	8%	33	26	29	23	6	5.49E-02
*FAM155A*	7	2%	7	4	2	0	5	7.68E-02

^a^”LOF” represents “loss of function” mutations, a category that includes nonsense, indel, and frameshift mutations.

**Fig. 1:**
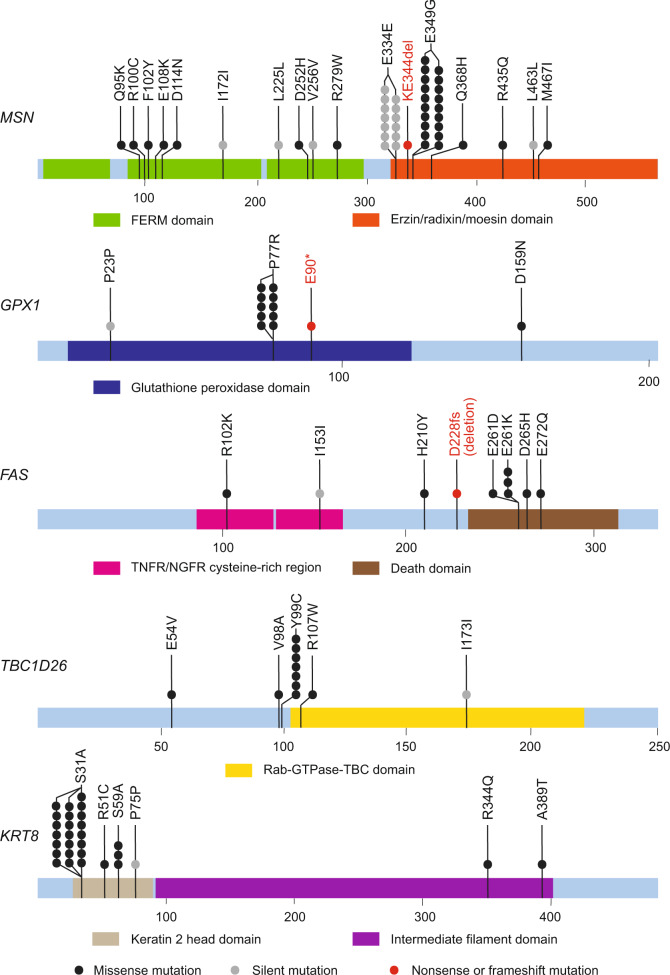
Somatic mutations in previously unreported significantly mutated genes (SMGs) among 430 exome-sequenced cervical carcinomas. Protein-coding portions of each SMG are displayed, and numbers refer to amino acid residues. Each highlighted section represents UniProt/Pfam functional domains. Circles represent a single mutation with black, gray, and red circles representing missense, silent, and nonsense or frameshift mutations, respectively.

Formerly unreported hotspot mutations were also identified, including E349G in *MSN*, P77R in *GPX1*, E261K/D in *FAS*, Y99C in *TBC1D26*, S31A in *KRT8* (Fig. 1), and in-frame PS120del mutations in *SPRED3*. Strikingly, although the *GPX1* P77R mutation was detected in only ten patients, it was associated with poor overall survival (*P* = 0.005, Supplementary Fig. 3).

Subset-based MutSig2CV analyses of SCCs revealed *PIK3CA* (26%), *MLL3* (20%), *MLL2* (15%), *EP300* (14%), *FBXW7* (9%), *FAT1* (9%), *PTEN* (7%), *RB1* (7%), *HLA-A* (7%), *RANBP2* (7%), and *MSN* (6%) as SMGs (Supplementary Table 4). Within the non-SCC cohort, *PIK3CA* (27%), *MLL3* (18%), *KRAS* (15%), *ARID1A* (13%), *TP53* (10%), *FBXW7* (8%), *PTEN* (7%), *ZC3H11A* (6%), *IL28A* (5%), and *AKT1* (5%) were identified as SMGs (Supplementary Table 5).

**Fig. 2:**
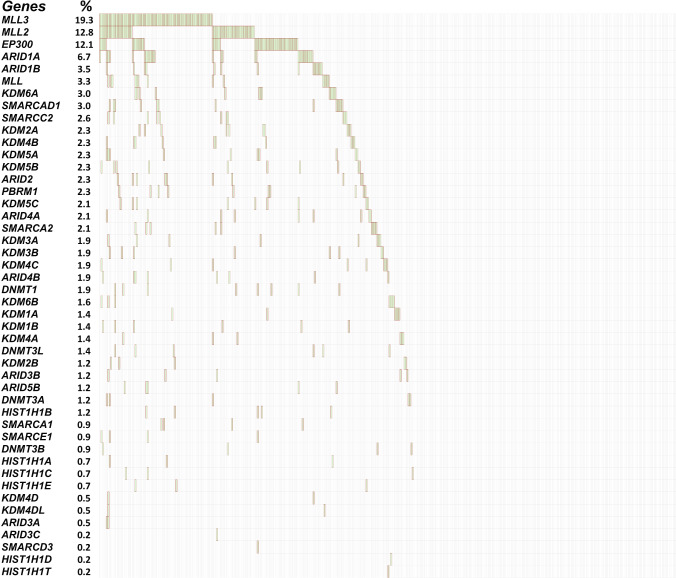
Frequency of mutations in chromatin remodeling genes in cervical carcinoma. Gene names are sorted in order of frequency with % of patients with gene mutation indicated next to each gene name. Each column represents one patient, and a dark red color represents a mutated gene.

The recognition of the chromatin remodifying genes *ARID1A, EP300, MLL2*, and *MLL3* as SMGs prompted the investigation of mutations in chromatin remodeling genes that did not meet the mutational significance (MutSig2CV) SMG threshold (Fig. 2). We observed that 228 of 430 (53%) of cervical tumors harbored at least one somatic mutation in a chromatin-modifying gene. This includes genes in the myeloid/lymphoid or mixed-lineage leukemia (MLL) family (27.7%), lysine (K)-specific demethylase (KDM) family (21.9%), AT-rich interactive domain (ARID) family (13.7%), SWI/SNF related, matrix associated, actin-dependent regulator of chromatin (SMARC) family (8.1%), histone cluster 1 (HIST1) family (3.7%), and the Polybromo 1 (*PBRM1*) gene (2.1%) (Supplementary Table 6).

### Copy number alterations in the combined set and by major histological classifications

**Fig. 3:**
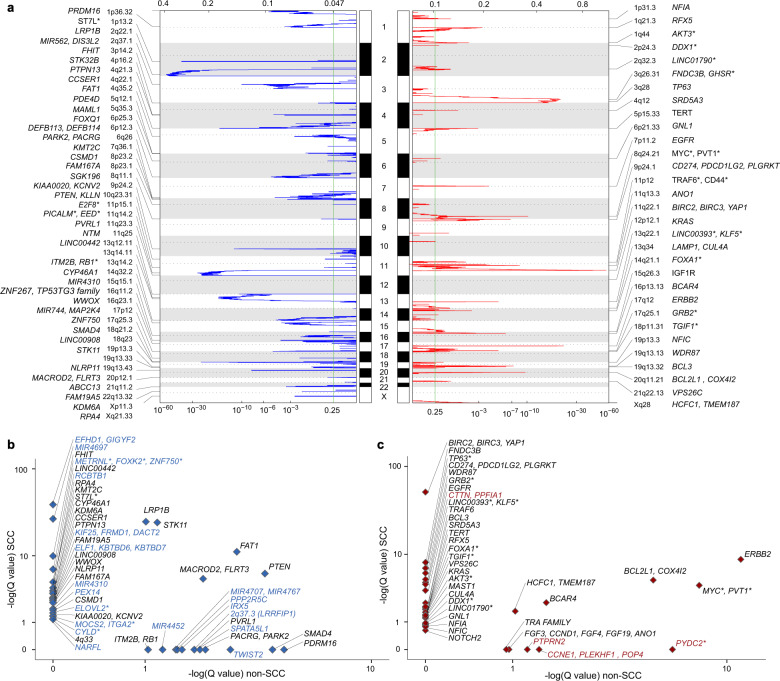
Focal somatic copy number alterations across 376 cervical carcinomas. **a** Somatic copy number alterations were analyzed by GISTIC. Chromosome positions are indicated along the *y* axis. On the *x axis*, focal deletions or amplifications are depicted with horizontal blue or red bars, respectively. The green line represents the significance threshold of *q* < 0.25 (the false discovery rate after multiple hypothesis testing). The locations of each peak region (cytoband) and the known or putative cancer-related genes within each peak are displayed. **b**, **c** Scatterplots showing the relationships between focal deletions (**b**) or amplification (**c**) in squamous cell carcinomas *(y* axis) and non-squamous cell carcinomas (*x* axis). Negative equivalents of log-transformed *Q* values are shown and the known or putative cancer-related genes within each chromosomal peak/region are displayed. The chromosomal position of the listed genes has been documented in the Cancer Gene Census. * denotes genes within the deleted/amplified region, but not within the peak.

Analyses of somatic copy number alterations across the entire dataset by GISTIC2.0 (9, 13) revealed (at a false discovery rate of *q* < 0.25) 31 significant focal amplifications and 43 significant focal deletions (Fig. 3a and Supplementary Table 7). Recurrent amplification events previously unreported in cervical cancer were identified (in genomic order, frequency in percentages) at 1p31.1 (*NFIA,* 31%), 1q21.3 (*RFX5,* 51%), 4q12 (*SRD5A3,* 6%), 6p21.33 (*GNL1*, 26%), 18p11.31 (*TGIF1*, 18%), 19q13.13 (*WDR87,* 12%), 19q13.2 (*NFIC,* 13%), and Xq28 (*HCFC1, TMEM187,* 23%) (Supplementary Table 7a). Recurrent deletion peaks/genes previously unreported in cervical cancer include 2q37.1 (*MIR562, DIS3L2,* 41%), 4q22.1 (*CCSER1*, 43%), 5q12.1 (*PDE4D*, 24%), 6p25.3 (*FOXQ1*, 16%), 6p26 (*PACRG, PARK2*), 8p23.2 (*CSMD1*, 9%), 11q14.2 (*PICALM, EED*, 38%), 11q23.3 (*PVRL1,* 54%), 11q25 (*NTM,* 53%), 14q32.2 (*CYP46A1,* 16%), 15q15.1 (*MIR4310,* 21%), 16q11.2 (*ZNF267, TP53TG3* family, 17%), and Xp11.3 (*KDM6A,* 22%) (Supplementary Table 7b). Pathway analyses using a combined set of single-nucleotide and copy number data across the whole cohort of cervical carcinomas revealed that ERBB- and PI3K/AKT/mTOR signaling were enhanced within mutated and/or amplified oncogenes (Supplementary Table 8), while p53 signaling and interferon signaling were enriched among deleted and mutated tumor suppressors (Supplementary Table 9).

Analyses of the SCC (*n* = 290) and non-SCC (*n* = 69) subsets revealed additional SCNAs. For brevity, SCNA peaks were highlighted as subtype-associated if the *q* value for a peak was <0.1 and (i) it was nearly identical to or lower than the *q* value for the combined set, or (ii) the peak occurred only in a particular subset but not in the combined set or other subset(s). The comparison of significant SCNA peaks/genes in SCC versus non-SCC subsets is shown in Fig. 3a (amplification) and 3b (deletions).

Amplifications peaks associated with the SCC subset include 1q44 (*AKT3*), 3q28 (*TP63*), 4q12 (*SRD5A3*), 7p11.2 (*EGFR*), 9p24.1 (*CD274, PDCD1LG2*), 11p12 (*TRAF6*), 11q13.3 (*CTTN, PPFIA1*), 11q22.1 (*YAP1, BIRC2, BIRC3*), 12p12.1 (*KRAS*), 13q22.1 (*LINC00393, KLF5)*, 17q25.1 *(GRB2*), 18p11.31 (*TGIF1*), 19q13.13 (*WDR87*), 19q13.32 (*BCL3*), and 21q22.13 (*VPS26C* also known as *DSCR3*) (Supplementary Table 10a). In the non-SCC subset, 3q26.2 (*PYDC2*), 7q36.3 (*PTPRN2*), and 19q12 (*CCNE1, PLEKHF1, POP4*) were unique amplification peaks (Supplementary Table 10b). Within the SCCs, amplified and mutated oncogenes were associated with apoptotic-, proliferative-, ERBB2-, and PI3K/AKT/mTOR signaling, regulation of cell death, and TNF signaling via NF-Κβ signaling (Supplementary Table 11), whilst in the non-SCC group, amplified and mutated oncogenes were associated with proliferation, cytokine signaling, and positive regulation of cell death (Supplementary Table 12).

SCC-associated deletion peaks included 1p36.22 (*PEX14*), 1p13.2 (*ST7L*), 2q37.1 (*EFHD1, GIGYF2*,), 3p14.2 (*FHIT*), 4q21.3 (*PTPN13*), 4q22.1 (*CCSER1*), 5q11.2 (*MOCS2, ITGA2*), 6p24 (*ELOVL2*), 6q27 (*KIF25, FRMD1, DACT2*), 8p23.1 (*FAM167A*), 8p23.2 *(CSMD1*), 9p24.2 (*KIAA0020, KCNV2*), 11q25 (*MIR4697*), 13q12.11 (*LINC00442*), 13q14.11 (*ELF1, KBTBD6, KBTBD7*), 13q14.2 (*RCBTB1*), 16q12.1 (*CYLD*), 16p13.3 (*NARFL*), 17q25.3 (*METRNL, FOXK2, ZNF750*), 18q23 (*LINC00908*), 19q13.43 (*NLRP11*), Xp11.3 (*KDM6A*), and Xq21.33 (*RPA4*) (Supplementary Table 13a). Deletion peaks associated with non-SCC tumors include 1p36.32 (*PRDM16*), 2q37.3 (*LRRFIP1*), 4q21.3 (*MIR4452*), 11q23.3, 14q32.31 (*PPP2R5C*), 15q21.1 (*SPATA5L1*), 16q12.2 (*IRX5*), 18q21.2 (*SMAD4*), and Xp22.31 (*MIR4707, MIR4767*) (Supplementary Table 13b). Overall, in the SCC group, deleted and/or mutated tumor suppressors were significantly associated with interferon α/β/γ response, positive regulation of developmental processes and apoptotic signaling (Supplementary Table 14), while in the non-SCC histological subgroup, genes associated with differentiation were enriched (Supplementary Table 15). Supplementary Tables 7, 10, and 13 describe known associations for some of the putative drivers in cervical cancer or other cancers for each of the subpopulations investigated.

### Copy number alterations based on mutational signatures

We also utilized hierarchical clustering methods to classify tumors based on the relative frequencies of the trinucleotide mutational contexts in each tumor. Three distinct clusters were identified (Supplementary Fig. 4, Supplementary Tables 16 and 19): tumors with predominantly *CpG mutations (*n* = 87), tumors with a preponderance of Tp*C mutations (*n* = 245), and tumors with a diverse array of other mutational signatures (*n* = 39). GISTIC analyses were performed for each of the mutational signature-derived subsets, and the significant focal peaks were compared and contrasted to identify subset-associated SCNAs in a manner similar to that described for histology-based subsets (Fig. 3 and Supplementary Figs. 5 and 6).

**Fig. 4:**
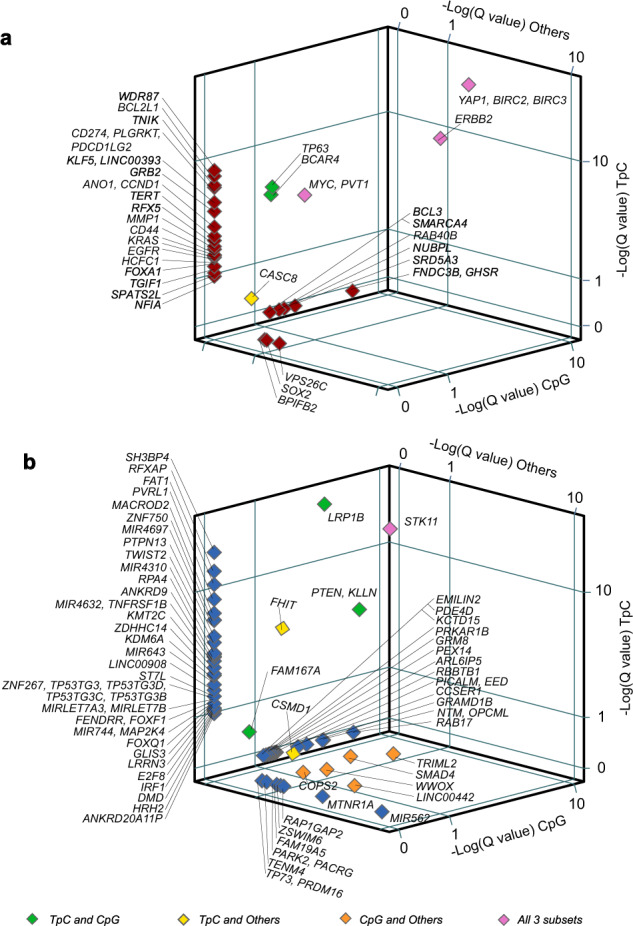
Copy number alterations within 371 cervical carcinomas with predominantly TpC (*n* = 245), CpG (*n* = 87), and other (*n* = 39) mutational profiles. Somatic copy number alterations were analyzed by GISTIC. 3D scatterplots showing the association between focal amplifications (**a**) and deletions (**b**) in tumors with predominantly different mutational signatures. Negative equivalents of log-transformed *Q* values of each focal copy number alteration and the cancer-related gene(s) within that chromosomal peak/region for TpC tumors (*y* axis), CpG tumors (*z* axis), and tumors with other mutational profiles (*x* axis).

Well-known oncogenes like *YAP1, BIRC2, BIRC3, ERBB2, MYC*, and *PVT1* were significantly amplified across all three groups (Fig. 4a), while the tumor suppressor *STK11* was significantly deleted across all three groups (Fig. 4b).

Tp*C subset-associated amplified genes included *WDR87*, *TNIK*, *CD274*, *PDCD1LG2, PDCD1LG2*, *KLF5, LINC00393, BCAR4, GRB2, ANO1, CCND1, TERT, RFX5, MMP1, CD44, KRAS*, *TGIF1, SPATS2L,* and *NFIA* (Supplementary Table 16a*);* while amplifications in *FNDC3B, GHSR, SRD5A3, RAB40B, NUBPL*, and *SMARCA4* were associated uniquely with the *CpG-predominant tumors (Supplementary Table 16b). The “Other” subset of tumors was associated with significant amplifications in *VPS26C*, *SOX2*, and *BPIFB2* (Supplementary Table 16c), while *TP63* and *BCAR4* were significantly amplified in both the Tp*C- and *CpG groups. Interestingly, Molecular Signatures Database (MSigDB) analyses revealed that genes amplified within TpC-predominant tumors were significantly associated with Notch, TNFα/NF-Κβ, and IL2-STAT signaling, epithelial to mesenchymal transition (EMT), and early estrogen response (Supplementary Tables 17 and 18). Details of all significantly amplified cytoband/genes are provided in Supplementary Table 16a–c and Supplementary Fig. 5.

Tp*C subset-associated focally deleted genes included *SH3BP4, RFXAP, FAT1, PVRL1, MACROD2, ZNF750, MIR4697, PTPN13, TWIST2, MIR4310, RPA4, ANKRD9, KMT2C, MIR4632, TNFRSF1B, ZDHHC14, KDM6A, MIR643, LINC00908, ST7L, ZNF267, TP53TG3* family*, MIRLET7A3, MIRLET7B, FENDRR, FOXF1, FOXQ1, MIR744, MAP2K4, GLIS3, LRRN3, E2F8, IRF1, DMD, HRH2*, and *ANKRD20A11P* (Supplementary Table 19a). Focal deletions in *RAB17, TRIML2, GRAMD1B, NTM, CCSER1, SMAD4, PICALM, EED, RCBTB1, ARL6IP5, PEX14, GRM8, PRKAR1B, KCTD15*, and *EMILIN2* were associated uniquely with the *CpG-predominant tumors (Supplementary Table 19b). The ‘Other’ subset of tumors had significant deletions in *MIR562, MTNR1A, LINC00442, PARK2, PARCG, CSMD1, RAP1GAP2, ZSWIM6, PRDM16, TP73, COPS2,* and *TENM4* (Supplementary Table 19c). In addition, *PTEN, KLLN,* and *LRP1B* were significantly deleted in both the Tp*C- and *CpG groups, *FHIT* deletions were associated with the Tp*C and “Other” groups, while *WWOX* was significantly deleted in the *CpG and “Other” subsets. The 4q35 centromeric region (including *TRIML2*) was significantly deleted in the *CpG- and “Other” groups. Details of all significantly deleted cytobands/genes are provided in Supplementary Table 19a-c and Supplementary Fig. 6.

### DNA damage repair (DDR) genes

**Fig. 5:**
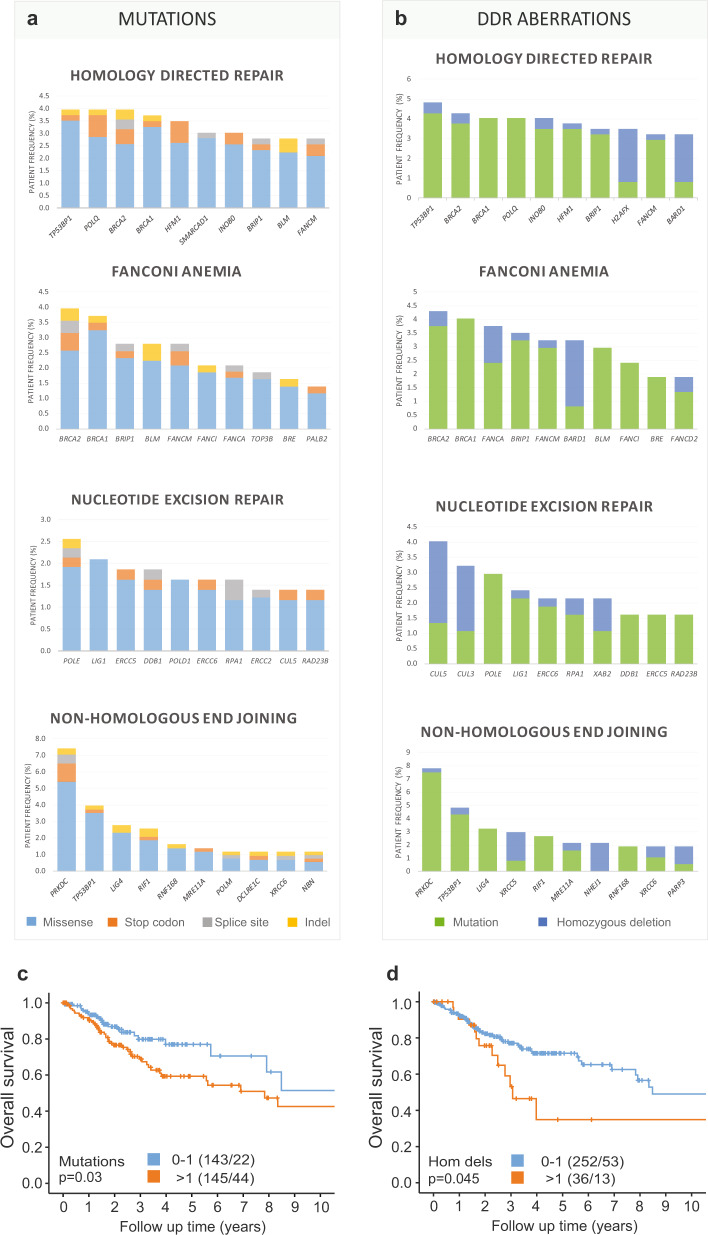
Single-nucleotide mutation and homozygous deletion frequencies in major DNA damage repair groups. **a**, **b** The ten genes with the highest frequency of mutation (left) and mutation plus homozygous deletion (right) within four major DNA damage repair (DDR) groups. **a** Mutation frequencies in DDR genes across 430 cervical carcinomas. Total mutation frequencies include missense (blue), stop codon (orange), splice site (gray), and indels (yellow). **b** Frequencies of DDR aberrations including mutation (green) and homozygous deletion (blue) in 372 cervical carcinomas. Overall survival for cervical cancer patients in relation to the total number of mutations (**c**) and homozygous deletions (**d**) in DDR genes are represented by Kaplan–Meier curves with probability values for Mantel–Cox log-rank test that compares categories. The number of patients and events are given in parentheses (patients/events). Hom dels homozygous deletions.

A high frequency of deletions and mutations within DNA damage repair (DDR) genes were detected in the investigated cervical carcinoma cohort (Fig. 5 and Supplementary Fig. 7). Therefore, we performed comprehensive analyses of mutations and copy number alterations occurring in ten major DDR pathways/subsets (see “Methods”). The distribution of mutations within five of these DDR groups is displayed in Fig. 5a. Genes in the homology-directed repair (HDR) pathway had the highest frequency of mutations being mutated in 342 patients, including *TP53BP1* (4.0%) and *POLQ* (4.0%). Fanconi Anemia (FA) genes were mutated in 152 patients, with *BRCA2* (4.0%), *BRCA1* (3.7%), and *BRIP1* (2.8%) mutations occurring most frequently. Nucleotide excision repair (NER) genes were mutated in 134 patients, and *POLE* had the highest frequency (2.6%). DNA end-joining (NHEJ) genes were mutated in 120 patients, and *PRKDC* had the highest frequency (7.4%).

The distribution of mutations plus homozygous deletions (homdels) for each DDR group is displayed in Fig. 5b for 372 patients. Frequently deleted DDR genes include the HDR gene *H2AFX* (2.7%), the HDR and FA gene *BARD1* (2.4%), the NER genes *CUL5* (2.7%) and *CUL3* (2.2%), and the NHEJ genes *XRCC5* (3.0%) and *NHEJ1* (2.2%).

Mutated DDR genes not assigned to any of the major DDR groups (“Others” group) include *PTEN* (7.7%), *TP53* (6.7%), *ATRX* (5.3%), *HERC2* (4.7%), *SMARCA4* (3.0%), *ATM* (3.0%), *TOPBP1* (2.8%), *ATR* (2.8%), *MDC1* (2.8%), and *CHEK2* (2.6%). In addition, deleted genes within the “Others” group include *PTEN* (3.2%), *ATM* (2.7%), and *CHEK1* (3.2%) (Supplementary Fig. 7).

High DDR mutation (>1) and homozygous deletion (>1) count were significantly associated with poor overall survival (*P* = 0.03 and *P* = 0.045, Fig. 5c and d, respectively).

### Clinical and functional investigation of *WDR87* amplification

**Fig. 6:**
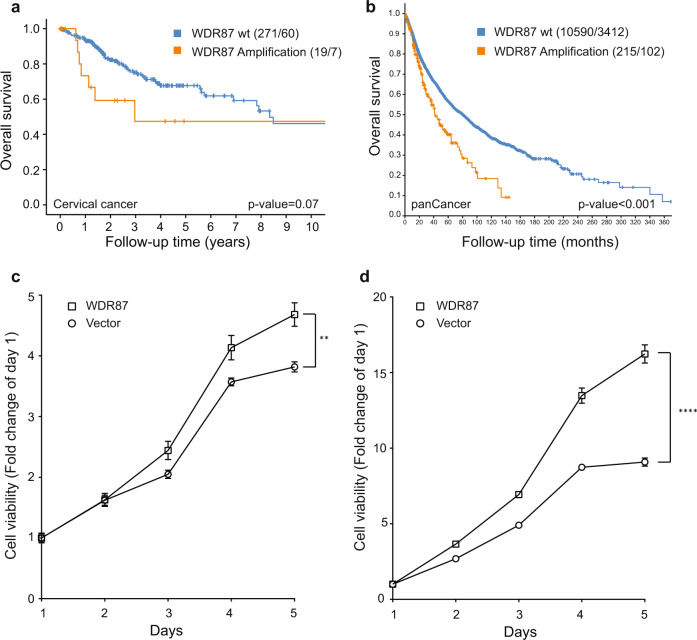
*WDR87* amplification in relation to survival and tumor cell growth. Overall survival in The Cancer Genome Atlas (TCGA) cervical cancer patients (*n* = 290) (**a**) and within the cBioPortal PanCancer Atlas Dataset (**b**) in relation to *WDR87* gene amplification status represented by a Kaplan–Meier curve with probability values for Mantel–Cox log-rank test that compares categories. The number of patients and events are given in parentheses (patients/events). Cellular proliferation assay in the cervical cancer cell line CRL1595 (**c**) and the kidney-derived HA1E cell line (**d**) comparing WDR87 overexpression *versus* WDR87 normal expression. The error bars represent standard error.

The chr19q13.13 copy number amplification peak harbored one gene, *WDR87*. Interestingly, we found a trend toward poorer survival for patients harboring high-level *WDR87* amplification (*P* = 0.07, Fig. 6a). This association was further reinforced within the cBioPortal PanCancer Atlas dataset (cBioPortal) as *WDR87* amplification was highly associated with poor overall survival (*P* = 3.49e-6, Fig. 6b). To investigate the functional implications of *WDR87* amplification, we performed transient transfection of *WDR87* constructs into a cervical cancer cell line CRL1595 and an immortalized embryonic kidney cell line HA1E. In both cases, cells overexpressing *WDR87* had higher rates of cellular proliferation than control cells (*P* < 0.01 and *P* < 0.001, Fig. 6c and d, respectively).

## Discussion

The large sample size in this study has facilitated the identification of previously unreported putative drivers and potential therapeutic targets in cervical cancer. We detected previously unreported recurrent mutations in cervical cancer in *GPX1, MSN, FAS, KRT8*, and *SPRED3*, all genes known to modulate tumorigenic processes. *GPX1* encodes the important antioxidant enzyme glutathione peroxidase 1^12^, and intriguingly, the recurrent P77R mutation in *GPX1* was significantly associated with worse overall patient survival. *MSN* encodes the ERM family member protein Moesin, which is implicated in cell adhesion, cell polarity, and migration^13^ known to influence invasive and metastatic abilities in tumor cells^14^. *FAS* encodes tumor necrosis factor superfamily member 6, a death receptor located on the cell surface involved in apoptosis, and previous studies have demonstrated that the E261K mutation exerts a strong dominant-negative effect by disrupting the interaction between FAS and FADD^15^. *SPRED3* encodes a tyrosine kinase-binding protein that inhibits ERK signaling^16^, and it is conceivable that the loss-of-function mutations in this gene may potentially promote tumorigenesis.

This study reveals frequent mutations in several chromatin remodeling gene families. Chromatin remodeling proteins are major players in cancer development and progression^17^ through the integration of the extracellular and cytoplasmic signals to control gene activity. Consequently, extensive dysregulation of chromatin remodelers and the resulting inappropriate expression of regulatory genes, contribute to carcinogenesis. Drugs targeting chromatin remodelers including BET proteins (BETi), histone methylation (EZH2i), histone acetylation (HDACi), and DNA methylation (DNMTi) may change gene expression in cancers and are currently being explored in several cancers^18^. Patients with tumors harboring extensive aberrations in chromatin remodeling genes could be candidates for such treatments.

We also performed comprehensive analyses of copy number data to systematically nominate drivers for almost all copy number peaks identified in this study. One of the previously unreported focal amplification peaks was chr19q13.13, which harbors one single gene, *WDR87*. Although little is known about the function of this 8 kb gene, we observed that *WDR87* amplification is associated with a poor prognosis. In addition, we performed functional experiments and demonstrated that overexpression of WDR87 increased cellular proliferation within both cell lines investigated, suggesting that this protein may in fact have tumor-promoting properties. Future studies will fully characterize the role of WDR87 and other putative drivers identified in this study.

Over 40% of the tumors harbored a deletion in cytoband 2q37.1. Similar to findings in Wilms’ tumor^19^, we identified *MIR562* as a putative driver in that deletion peak. *MIR562* has been shown to reduce the expression of c-MET in glioblastoma cells by directly binding to its 3’-UTR^20^. Considering that c-MET overexpression has recently been shown as a prognostic marker in cervical cancer^21,22^, the potential role of Met inhibition in cervical cancers with 2q37 deletions is a worthy area for future studies.

Recent studies have provided a deeper understanding of nucleotide mutational patterns in cancer with the characterization of mutational signatures^23^. We and others have previously shown that signature 2 and 13, which are both attributed to the activity of the AID/APOBEC family of cytidine deaminases, are enriched in cervical cancer^5,6,24^. APOBEC3B, known to be upregulated in cervical cancer^25^, is a major player in this process, and its activity is closely linked to the predominant mutational signatures (*CpG and Tp*C) identified in this study. Interestingly, we observed striking differences in the copy number alterations associated with *CpG- versus Tp*C-predominant tumors. Comparison of GISTIC outputs between these two groups revealed 14 amplification events including *CD274*, *EGFR*, and *KRAS* and 32 deletion events were unique to the Tp*C tumors, whereas six amplification events and 16 deletion events were unique to the *CpG tumors. Our findings suggest that the mutational processes associated with different signatures may elevate the importance of some copy number alterations for cervical carcinogenesis, while perhaps making others redundant.

We detected a broad range of mutations and homozygous deletions in DDR genes. DNA damage and mutagenesis are enabling hallmarks of genomic instability in cancer^26^. Patients with advanced cervical cancer typically receive radiotherapy in combination with platin-based chemotherapy. These platinum-based agents generate inter- and intra-strand DNA crosslinks, which affect DNA unwinding and subsequently blocking DNA replication, leading to cytotoxicity predominantly in the S-phase. In this process, single-stranded DNA becomes exposed and recruits ATRIP, which further triggers the ATR signaling axis to stabilize and restart replication. In addition, ATR regulates cell cycle progression by activating Chk1 (gene name: *CHEK1*), which in turn activates p53. ATR is also involved in double-strand DNA breaks by activating BRCA1, among others. ATM plays a key role in double-strand DNA break repair by inducing cell cycle arrest through activating Chk2 (gene name: *CHEK2*). This study revealed frequent deletions and mutations in all the aforementioned genes pointing to previously unreported DDR inhibiting strategies for cervical cancer. Based on preclinical evidence, the response to chemo/radiation therapy may be increased using ATM or Chk1 inhibitors for cervical cancer patients^27^. In addition, we observed mutations in *PRKDC* which encodes a key component in the NHEJ pathway and is regarded as a potential prognostic marker for chemo-resistance in breast, colorectal and gastric cancer^28–30^. Functional studies of PRKDC expression in relation to chemosensitivity and as a possible target for treatment in cervical cancer would therefore be of high interest.

In summary, this comprehensive analysis of 430 cervical carcinomas has revealed oncogenic alterations involving multiple pathways including chromatin remodeling and DNA damage repair. We have identified specific drivers associated with mutational signatures while providing a detailed compendium of putative copy number drivers in cervical cancers. These findings lay a broad foundation for developing novel prognostic and therapeutic applications in cervical cancer.

## Methods

### Ethical statements

This study was approved by the institutional review board of the University of Alabama at Birmingham (IRB-160517009). All participants gave written informed consent prior to inclusion in the respective cohorts.

### Mutational analyses

Whole-exome sequencing data from 430 cervical carcinomas were analyzed by combining our previous published work^6,10^ and publicly available data generated by TCGA (https://portal.gdc.cancer.gov/)^5^. The Mutect2.0 and MutSig2CV algorithms were used to identify somatic mutations and significantly mutated genes (SMGs), respectively^31,32^ in the entire cohort and on subsets of 327 squamous cell carcinomas and 83 non-squamous carcinomas (including adenocarcinomas, adenosquamous, and neuroendocrine tumors).

MutSig2CV analysis identifies genes that are mutated more frequently than expected by chance given background mutational processes and other covariates. Genes were determined to be significantly mutated by MutSig2CV analyses if they had a false discovery rate of *q* < 0.1 after correction for multiple hypothesis testing.

### Copy number alterations

SNP6.0 copy number data from 376 cervical carcinomas were analyzed. We derived the data from combining our previously published work^6^ with publicly available data generated by the TCGA (https://portal.gdc.cancer.gov/)^5^. Copy number alterations were analyzed using the GISTIC2.0 algorithm^33,34^. *q* values of <0.25 were considered significant. In general, the nomination of putative target driver genes in each focal copy number alteration peak was determined by a combination of the GISTIC peak output, a confirmatory search on the UCSC Genome Browser, and a literature search to determine if there were previous reports of the gene(s) exhibiting tumor-promoting or tumor-inhibiting properties (for amplifications and deletions, respectively). If a gene was listed as being present in a narrow peak and it was confirmed by UCSC genome browsing with literature search support for the expected role, it was nominated as a putative target driver gene for that peak. If no gene in the narrow peak was supported by literature searches, other genes in the wider peak were subjected to similar UCSC and literature searches, and the gene(s) closest in genomic distance to the narrow peak was/were nominated as putative target driver genes for that peak. These second-tier driver genes are denoted with asterisks (*) in the figures. Multiple putative driver genes are listed whenever there is the discrepancy between the list of gene(s) in the peak(s) and the results of UCSC genome searches.

### Mutational signature analyses

The frequencies of the 96 trinucleotide contexts were aggregated into three groups for each patient: Tp*C, *CpG, and Others. The frequencies of Tp*C and *CpG mutations were adjusted by redistributing Tp*CpG mutations proportionately to each group, based on the relative frequencies of the other Tp*C and*CpG mutations in each tumor. Then, the relative frequencies of Tp*C, *CpG, and non-Tp*C-non-*CpG (Others) were subjected to hierarchical clustering which resulted in three tumor clusters: “TpC-predominant”, “CpG-predominant”, and “Others”. These three categories were subsequently used for downstream analyses.

### Integrated pathway analyses

In order to identify altered molecular pathways and gene sets, the lists of SMGs (derived from MutSig2CV analyses) or copy number altered genes (derived from GISTIC2.0 analyses) were investigated in the MSigDB^35^ with a focus on gene sets c2 (curated gene sets), c5 (gene ontology gene sets), and H (Hallmark gene sets). Enhanced biological processes were identified by combining the lists of mutated or amplified oncogenes while attenuated molecular pathways were identified using lists of mutated or deleted tumor suppressor genes.

### DNA damage repair genes

We used a previously curated list of 276 DNA repair pathway genes^36^ as our baseline set for analyses (Supplementary Table 20a). Of these, 255 genes overlapped with genes reported in the mutational allelic frequency (MAF) file and were investigated for point mutations in 430 patients and for homozygous deletions in 372 patients with available copy number data (Supplementary Table 20b). Most of the genes were assigned to one or more well established functional DNA damage response (DDR) pathways: base excision repair (BER), direct damage reversal/repair (DR), damage sensor (DS), the Fanconi anemia (FA), homology dependent recombination (HR), mismatch repair (MMR), nucleotide excision repair (NER), non-homologous end joining (NHEJ), nucleotide pool maintenance (NP), and translesion DNA synthesis (TLS). Other genes known to modulate DDR (e.g., *PTEN, TP53*) were also included. The frequencies of point mutations and copy number deletions in genes from each DDR group were extracted from MutSig2CV and GISTIC outputs, respectively.

### *WDR87* transfection and cellular proliferation assays

The cDNA of *WDR87* (variant 1, NM_001291088.1) was synthesized (GenScript, Piscataway, NJ) and cloned into the BamHI site of pcDNA 3.1^+^/C-(K)-DYK and subcloned into BamHI/XhoI site of pReceiver-203 (GeneCopoeia, Rockville, MD). Plasmids were prepared following the manufacturer’s instructions (Zymo Research, Irvine, CA). The cervical cancer cell line CRL1595 and the immortalized embryonic kidney cell line HA1E were transfected with *WDR87* cDNA expression clones and the vectors (pcDNA 3.1^+^/C-(K)-DYK, and pReceiver-203) using EndoFectin-Max (GeneCopoeia, Rockville, MD). In addition, HA1E cells transfected with *WDR87* and its vector pReceiver-203 were gated in a single-parameter histogram for the EGFP and 5 × 10^4^ cells were collected in DMEM with 10% FBS (Comprehensive Flow Cytometry Core at UAB), plated in 10-cm culture plates and expanded up to confluence. In all cases, cells were seeded in 24-well plates and cultured for up to 4 days. The number of live cells was measured at wavelength 570 nm using the MTT assay for mitochondrial enzymatic activity (Promega Corp, Madison, WI). The cell viability was calculated as the fold change of cells seeded at day 0, respectively. The viabilities were compared by paired *t* test. Experiments were duplicated and representative data are presented. Experiments were duplicated and representative data are presented. The means and standard errors of viabilities were compared by paired *t* test (*P* < 0.05)

### Clinicopathological and survival analyses

Clinicopathological data were analyzed by using the Software package SPSS Statistics (Statistical Package of Social Science) version 25.0 (IBM, Armonk, USA). All probability values were two-sided and considered statistically significant if <0.05. Correlation between groups was assessed using Pearson *χ*^2^ or Fisher´s exact test as appropriate for categorical variables, whilst the Mann–Whitney *U* or the Kruskal–Wallis test was applied as appropriate for continuous variables. Due to variability in follow-up recordings between the three patient cohorts, survival analyses were performed solely within the TCGA cohort. Overall survival was calculated from the date of primary treatment until death. The survival analyses on the PanCancer Atlas Dataset were performed within the cBioPortal framework. Patient survival analyses were performed by applying the Kaplan–Meier (product-limit) method, and survival differences were determined by the log-rank test (Mantel–Cox).

### Reporting summary

Further information on research design is available in the Nature Research Reporting Summary linked to this article.

## Supplementary information

Reporting Summary

Supplementary Information

## Data Availability

The TCGA datasets used and/or analyzed during this study are available in dbGaP (accession number phs000178)^5^. The Ojesina et al. dataset is available in dbGaP (accession number phs000600)^6^. The Chung et al. dataset is available in dbGaP (accession number phs000723)^10^.

## Code availability

All applied software versions are described in “Methods”. Mutect2.0 can be accessed through https://software.broadinstitute.org/cancer/cga/mutect, while MutSig2CV can be accessed through https://software.broadinstitute.org/cancer/cga/mutsig. GISTIC2.0 can be accessed through ftp://ftp.broadinstitute.org/pub/GISTIC2.0/all_versions. No custom scripts were created for the analyses within this paper.
