# Supplementary material for: Genomic alterations associated with mutational signatures, DNA damage repair and chromatin remodeling pathways in cervical carcinoma
Source: NPJ Genom Med. 2021 Oct 7;6:82. doi: 10.1038/s41525-021-00244-2 (PMC8497615; doi:10.1038/s41525-021-00244-2)
Supplement: Supplementary file 1 — Reporting Summary [file 41525_2021_244_MOESM1_ESM.pdf]

nature portfolio

Corresponding author(s): Akinyemi I. Ojesina

Last updated by author(s): Aug 10, 2021

Reporting Summary

Nature Portfolio wishes to improve the reproducibility of the work that we publish. This form provides structure for consistency and transparency in reporting. For further information on Nature Portfolio policies, see our Editorial Policies and the Editorial Policy Checklist.

Statistics

For all statistical analyses, confirm that the following items are present in the figure legend, table legend, main text, or Methods section.

n/a	Confirmed	
[ ]	[x]	The exact sample size (*n*) for each experimental group/condition, given as a discrete number and unit of measurement
[ ]	[x]	A statement on whether measurements were taken from distinct samples or whether the same sample was measured repeatedly
[ ]	[x]	The statistical test(s) used AND whether they are one- or two-sided<br>*Only common tests should be described solely by name; describe more complex techniques in the Methods section.*
[x]	[ ]	A description of all covariates tested
[ ]	[x]	A description of any assumptions or corrections, such as tests of normality and adjustment for multiple comparisons
[ ]	[x]	A full description of the statistical parameters including central tendency (e.g. means) or other basic estimates (e.g. regression coefficient) AND variation (e.g. standard deviation) or associated estimates of uncertainty (e.g. confidence intervals)
[x]	[ ]	For null hypothesis testing, the test statistic (e.g. *F*, *t*, *r*) with confidence intervals, effect sizes, degrees of freedom and *P* value noted<br>*Give P values as exact values whenever suitable.*
[x]	[ ]	For Bayesian analysis, information on the choice of priors and Markov chain Monte Carlo settings
[x]	[ ]	For hierarchical and complex designs, identification of the appropriate level for tests and full reporting of outcomes
[x]	[ ]	Estimates of effect sizes (e.g. Cohen's *d*, Pearson's *r*), indicating how they were calculated

Our web collection on statistics for biologists contains articles on many of the points above.

Software and code

Policy information about availability of computer code

Data collection: N/A

Data analysis: The Mutect2.0 and MutSig2CV algorithms were used to identify somatic mutations and significantly mutated genes (SMGs), respectively. Mutect2.0 can be accessed through https://software.broadinstitute.org/cancer/cga/mutect while MutSig2CV can be accessed through https://software.broadinstitute.org/cancer/cga/mutsig.
Copy number alterations were analyzed using the GISTIC2.0 algorithm which can be accessed through ftp://ftp.broadinstitute.org/pub/GISTIC2.0/all_versions. The lists of SMGs (derived from MutSig2CV analyses) or copy number altered genes (derived from GISTIC2.0 analyses) were investigated on the MSigDB website (https://www.gsea-msigdb.org/gsea/msigdb/).

For manuscripts utilizing custom algorithms or software that are central to the research but not yet described in published literature, software must be made available to editors and reviewers. We strongly encourage code deposition in a community repository (e.g. GitHub). See the Nature Portfolio guidelines for submitting code & software for further information.

Data

Policy information about availability of data

All manuscripts must include a data availability statement. This statement should provide the following information, where applicable:

- Accession codes, unique identifiers, or web links for publicly available datasets
- A description of any restrictions on data availability
- For clinical datasets or third party data, please ensure that the statement adheres to our policy

The TCGA data sets used and/or analysed during the current study are available in dbGaP (accession number phs000178). The Ojesina et al data set is available in dbGaP (accession number phs000600). The Hong Kong dataset (from Chung et al) is available in dbGaP (accession number phs000723).

Field-specific reporting

Please select the one below that is the best fit for your research. If you are not sure, read the appropriate sections before making your selection.

☒ Life sciences ☐ Behavioural & social sciences ☐ Ecological, evolutionary & environmental sciences

For a reference copy of the document with all sections, see nature.com/documents/nr-reporting-summary-flat.pdf

Life sciences study design

All studies must disclose on these points even when the disclosure is negative.

Sample size	This is a retrospective study gathering genomic data from all publically available sources in addition to our own data comprising the largest genomic study on WES sequenced cervical carcinomas to date (n=430). Multiple testing was performed on p-values.
Data exclusions	No data was excluded from the study.
Replication	All in silico and in vitro analyses were performed at least twice to ensure reproducibility.
Randomization	No random subgroupings were performed within this study. Subgroup analyses were performed within and between lesions with different histologic type and/or mutational profile.
Blinding	N/A

Reporting for specific materials, systems and methods

We require information from authors about some types of materials, experimental systems and methods used in many studies. Here, indicate whether each material, system or method listed is relevant to your study. If you are not sure if a list item applies to your research, read the appropriate section before selecting a response.

Materials & experimental systems

n/a	Involved in the study	
☒	☐	Antibodies
☐	☒	Eukaryotic cell lines
☒	☐	Palaeontology and archaeology
☒	☐	Animals and other organisms
☐	☒	Human research participants
☐	☒	Clinical data
☒	☐	Dual use research of concern

Methods

n/a	Involved in the study	
☒	☐	ChIP-seq
☒	☐	Flow cytometry
☒	☐	MRI-based neuroimaging

Eukaryotic cell lines

Policy information about cell lines

Cell line source(s)	CRL1595 was obtained from ATCC while HA1E was a kind gift from Dr. William Hahn (Dana-Farber Cancer Institue).
Authentication	Cell lines received no further authentication.
Mycoplasma contamination	All cell lines tested negative for mycoplasma contamination.
Commonly misidentified lines (See ICLAC register)	N/A

Human research participants

Policy information about studies involving human research participants

Population characteristics	Cervical cancer patients with fresh frozen tumors with a tumor cell content above 50% and preferably 80% with available WES and SNP data were included in this study.
Recruitment	Clinical data from the TCGA cohort was retrieved from the publicly available cBioPortal; for the Hong Kong cohort, from already published data; and for the Ojesina cohort, from review of patient records. All participants gave written informed consent prior to inclusion in the respective cohort.

Ethics oversight	University of Alabama at Birmingham

Note that full information on the approval of the study protocol must also be provided in the manuscript.

Clinical data

Policy information about clinical studies

All manuscripts should comply with the ICMJE guidelines for publication of clinical research and a completed CONSORT checklist must be included with all submissions.

Clinical trial registration	N/A
Study protocol	N/A
Data collection	N/A
Outcomes	N/A
