# Supplementary material for: Genomic alterations associated with mutational signatures, DNA damage repair and chromatin remodeling pathways in cervical carcinoma
Source: NPJ Genom Med. 2021 Oct 7;6:82. doi: 10.1038/s41525-021-00244-2 (PMC8497615; doi:10.1038/s41525-021-00244-2)
Supplement: Supplementary file 2 — Supplementary Information [file 41525_2021_244_MOESM2_ESM.pdf]

Supplementary Information

Genomic alterations associated with mutational signatures, DNA damage repair and chromatin remodeling pathways in cervical carcinoma

Mari K. Halle[1,2,3], Aishwarya Sundaresan[4], Jianqing Zhang[3], Chandra Sekhar Pedamallu[3], Vinodh Srinivasasainagendra[4], Jessica Blair[3], Dewey Brooke[3], Bjørn I. Bertelsen[5], Kathrine Woie[1], Sadeep Shrestha[3], Hemant Tiwari[4], Yick Fu Wong[6], Camilla Krakstad[1,2]*, Akinyemi I. Ojesina[3,7,8]*#

[1]Department of Obstetrics and Gynaecology, Haukeland University Hospital, Bergen, Norway [2]Centre for Cancer Biomarkers, Department of Clinical Science, University of Bergen, Norway [3]Department of Epidemiology, University of Alabama at Birmingham, Birmingham, Alabama [4]Department of Biostatistics, University of Alabama at Birmingham, Birmingham, Alabama [5]Department of Pathology, Haukeland University Hospital, Bergen, Norway [6]Department of Obstetrics and Gynecology, The Chinese University of Hong Kong, Prince of Wales Hospital, Shatin, Hong Kong [7]O'Neal Comprehensive Cancer Center, University of Alabama at Birmingham, Birmingham, Alabama [8]HudsonAlpha Institute for Biotechnology, Huntsville, Alabama

*Shared last authors #Corresponding author

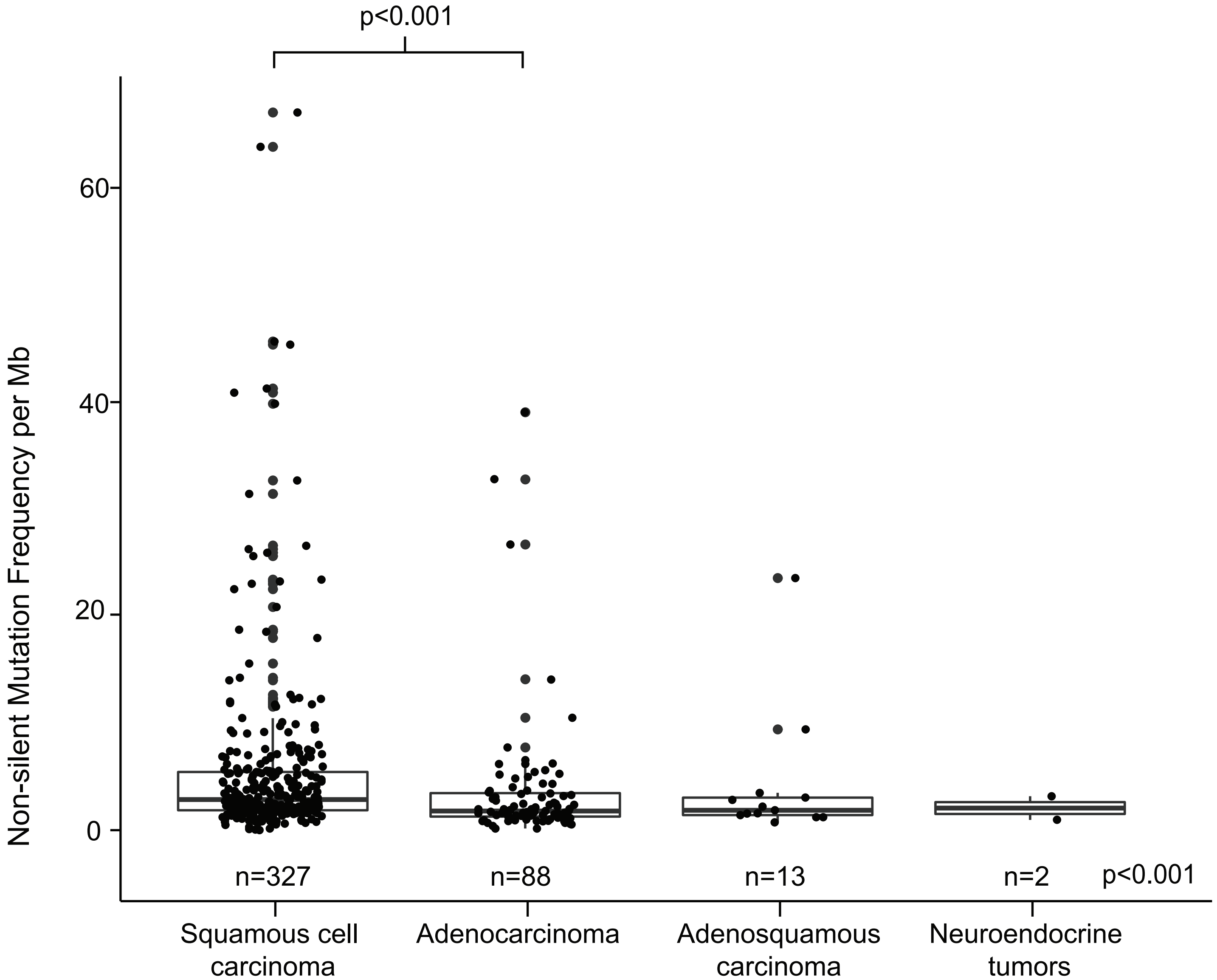

Supplementary Figure 1. Non silent mutation rate related to histologic types. Abbreviations: MB: Megabase.

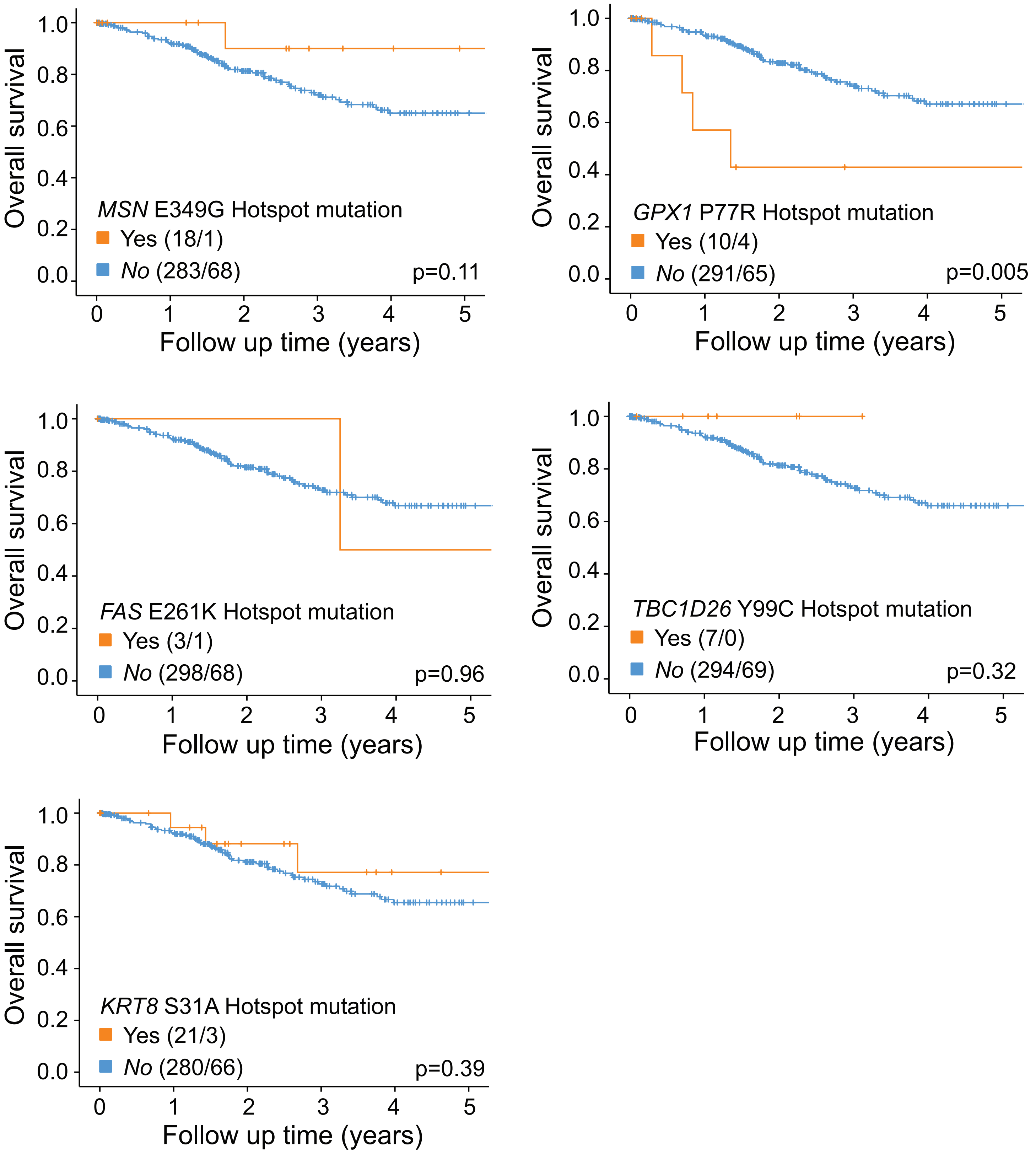

Supplementary Figure 2. Selected recurrent hotspot mutations in relation to survival. Overall survival for cervical cancer patients in relation to hotspot mutation status represented by Kaplan-Meier curves with probability values for Mantel-Cox log rank test that compares categories. The number of patients and events are given in parentheses (patients/events).

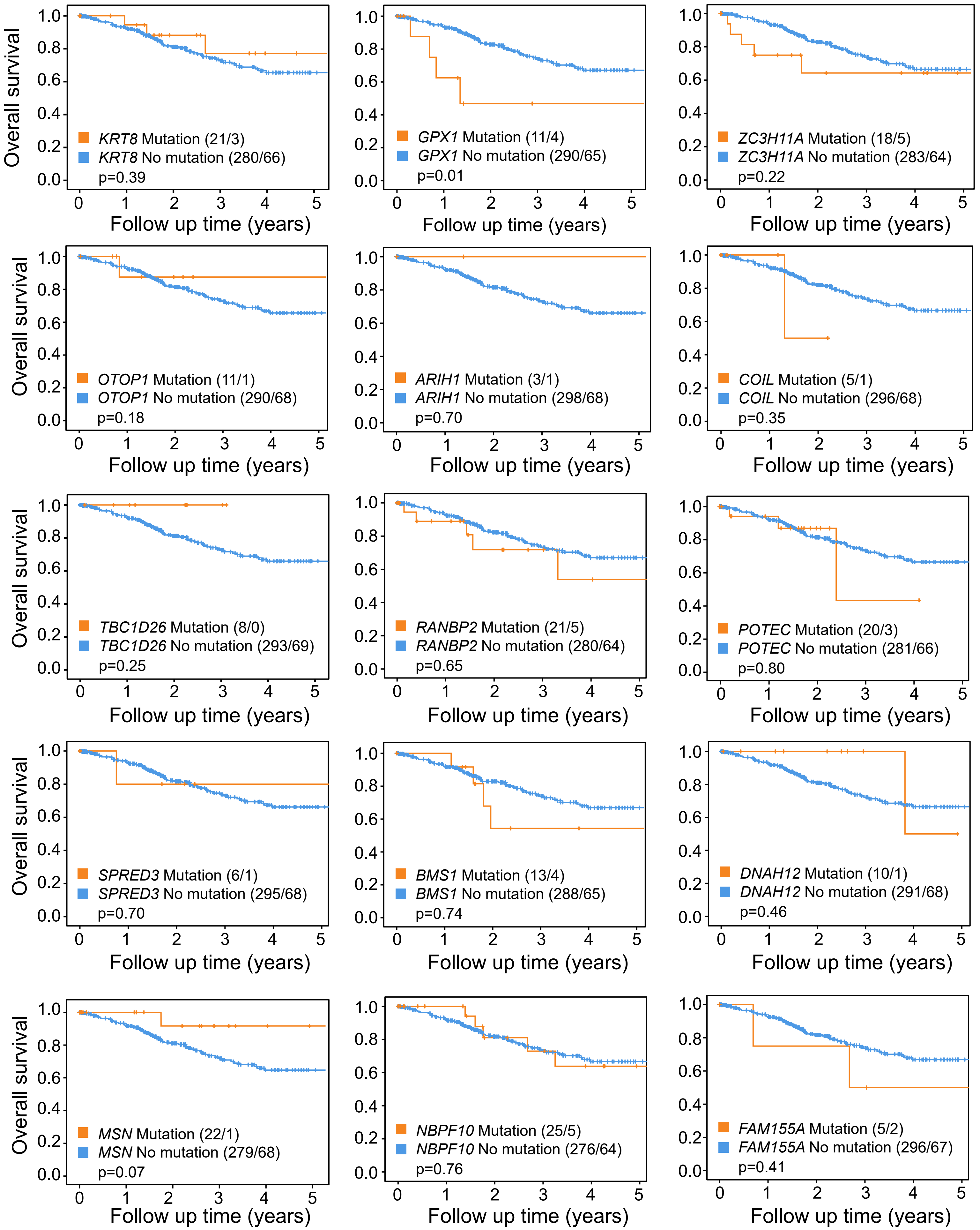

Supplementary Figure 3. Mutated previously unreported significantly mutated genes in relation to survival. Overall survival for cervical cancer patients in relation to mutation status of all novel significantly mutated genes discovered within this study. Kaplan-Meier curves are presented with probability values for Mantel-Cox log rank tests that compare categories. The number of patients and events are given in parenthesis (patients/events).

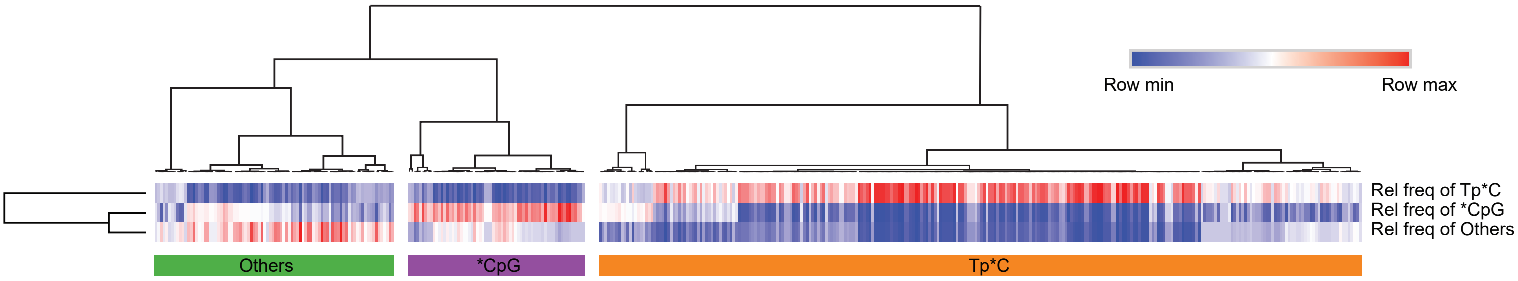

Supplementary Figure 4. Hierarchical clustering of the relative frequencies of the trinucleotide mutational contexts in each tumor.
Abbreviations: Rel freq: Relative frequencies

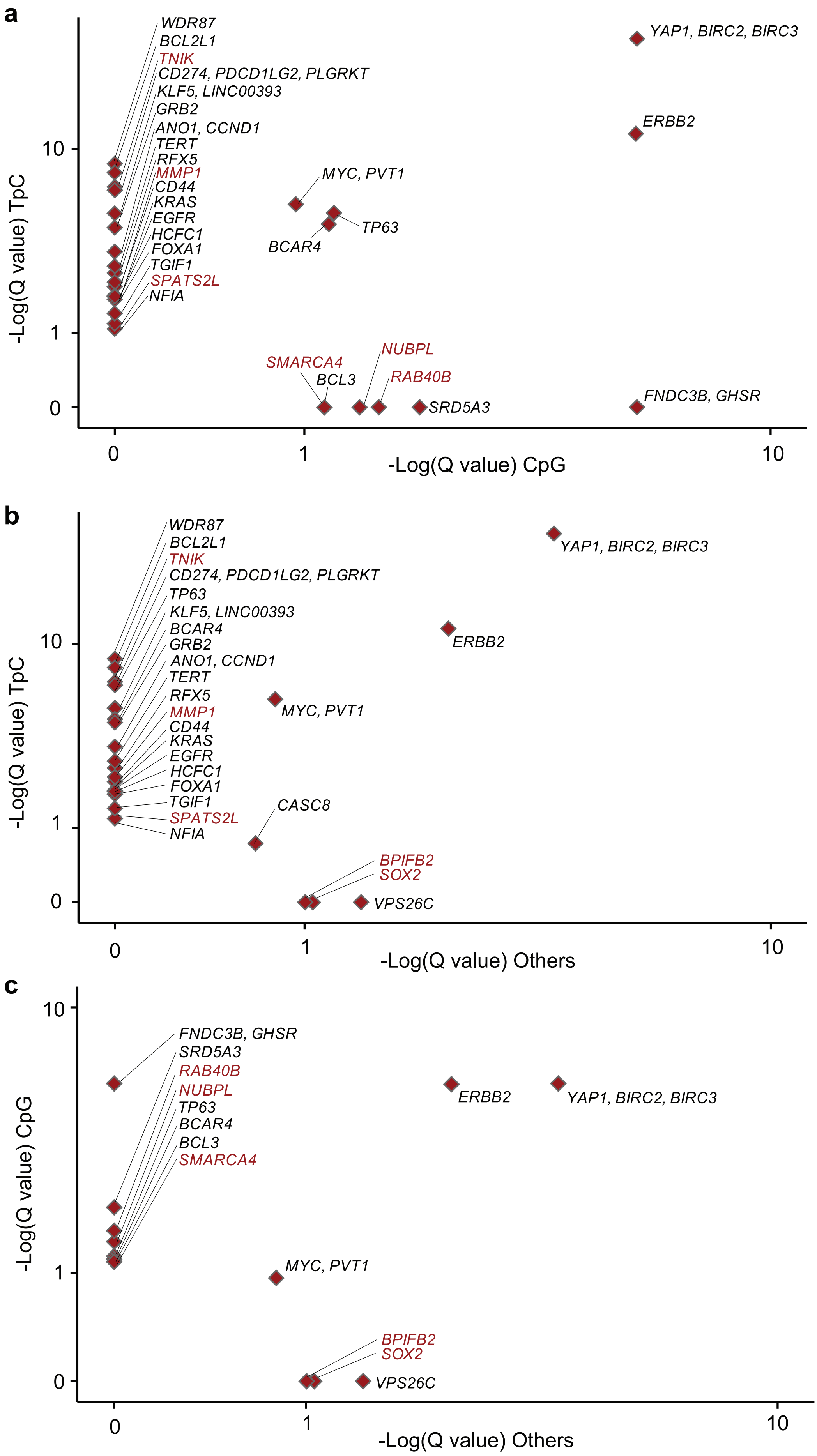

Supplementary Figure 5. Focal amplification events within 371 cervical carcinomas with predominantly TpC (n=245), CpG (n=87) and other (n=39) mutational profiles. Somatic copy number alterations were analyzed by GISTIC. 2D scatterplots showing the association between focal amplifications in tumors with predominantly different mutational signatures. Negative equivalents of log transformed Q values of each focal amplification event and the cancer-related gene(s) within that chromosomal peak/region for TpC tumors (*y-axis*) vs CpG tumors (*x-axis*) (**a**), TpC tumors (*y-axis*) vs tumors with other mutational profiles (x-axis) (**b**) and CpG tumors vs tumors with other mutational profiles (*x-axis*) (**c**). Cancer related genes in peaks unique to the specific subgroup/not found in the full dataset are outlined in red. Chromosomal position of the listed genes has been documented in the Cancer Gene Census.

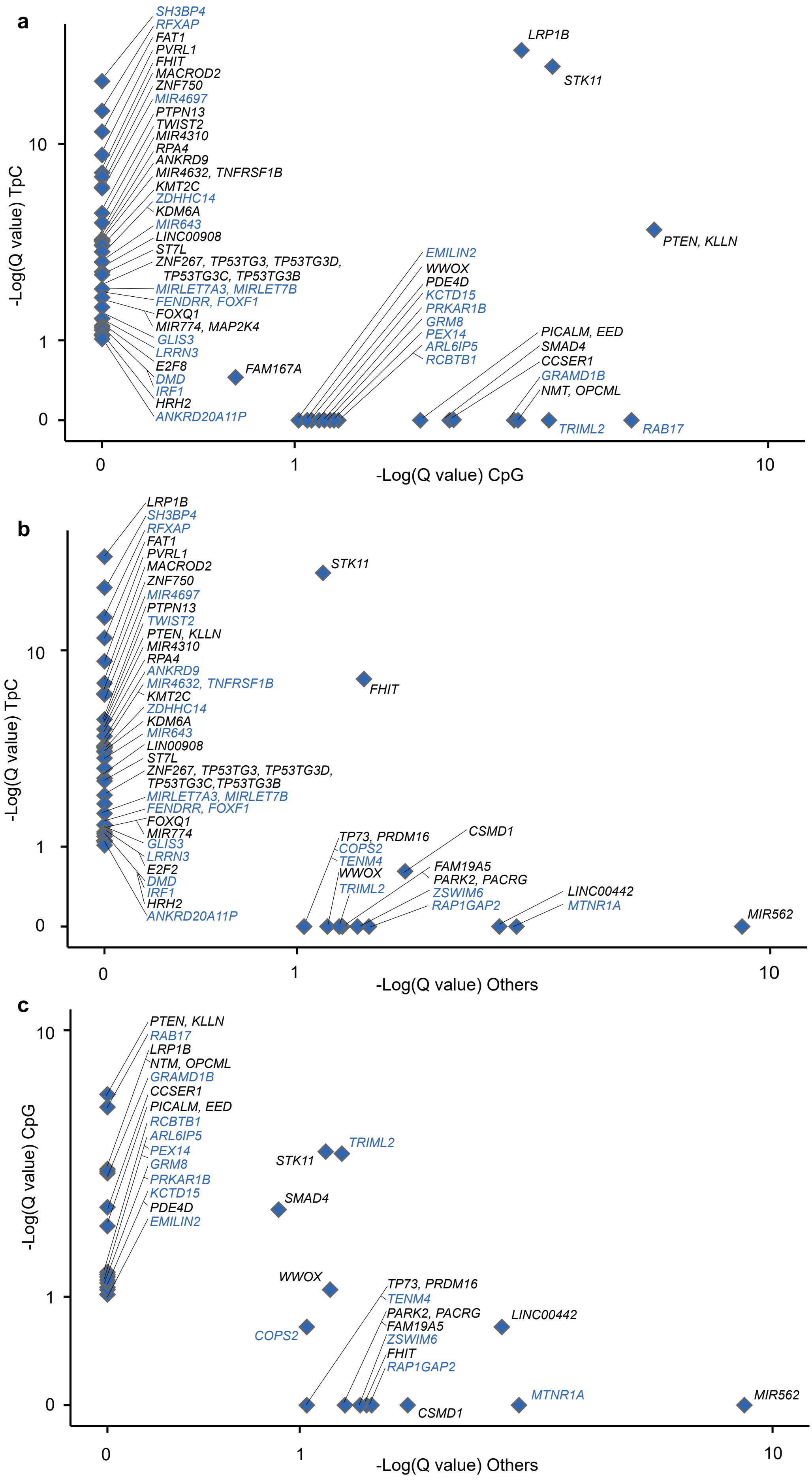

Supplementary Figure 6. Focal deletion events within 371 cervical carcinomas with predominantly TpC (n=245), CpG (n=87) and other (n=39) mutational profiles. Somatic copy number alterations were analyzed by GISTIC. Scatterplots showing the association between focal deletions in tumors with predominantly different mutational signatures. Negative equivalents of log transformed Q values of each focal deletion event and the cancer-related gene(s) within that chromosomal peak/region for TpC tumors (*y-axis*) vs CpG (*x-axis*) (**a**), TpC (*y-axis*) vs other mutational profiles (*x-axis*) (**b**) and CpG vs other mutational profiles (*x-axis*) (**c**). Cancer related genes in peaks unique to the specific subgroup/not found in the full dataset are outlined in blue. Chromosomal position of the listed genes has been documented in the Cancer Gene Census.

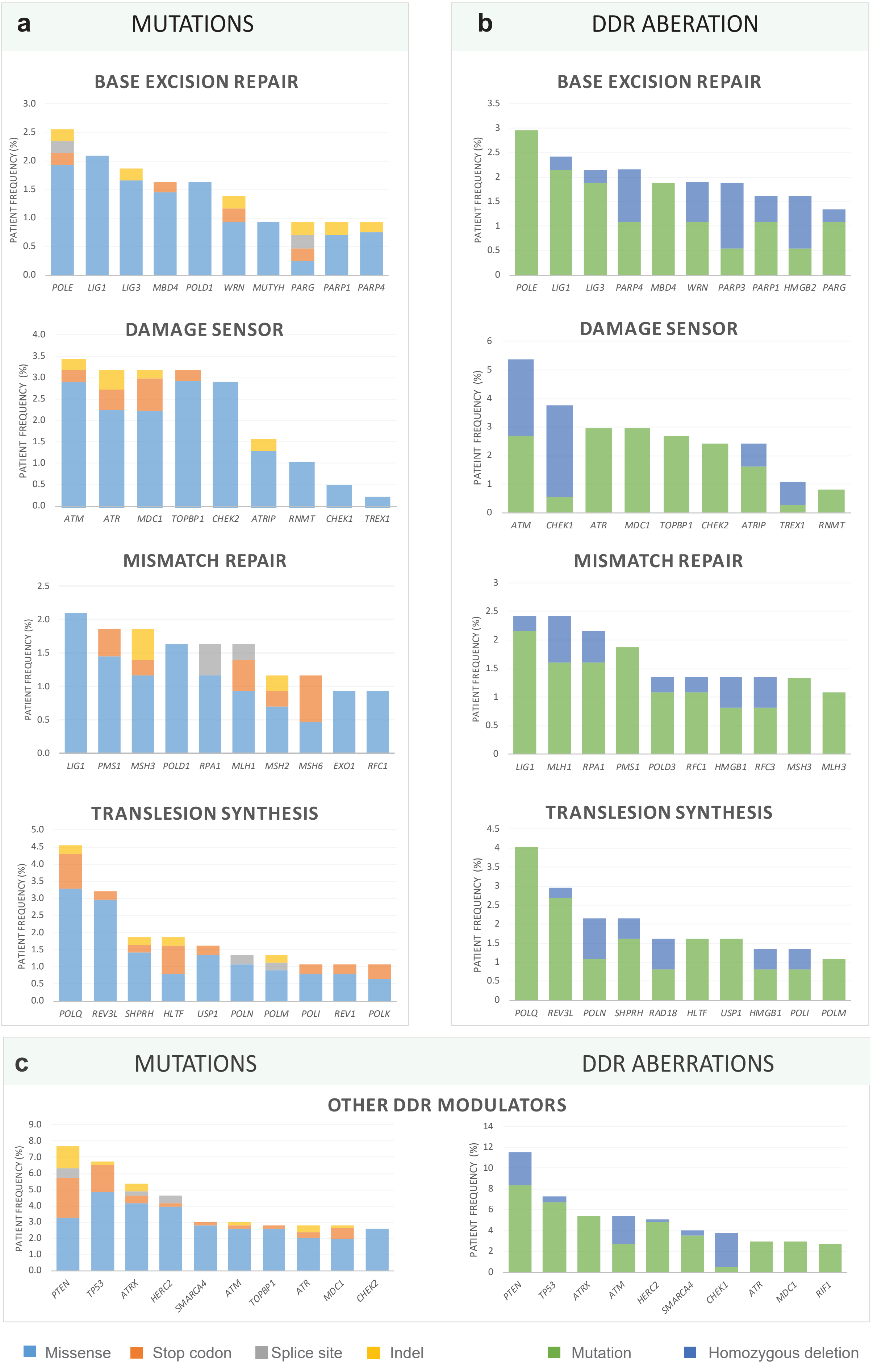

Supplementary Figure 7. Single nucleotide mutation and homozygous deletion frequencies in other DNA damage repair (DDR) genes. The ten genes with highest frequency of mutation (left) and mutation plus homozygous deletion (right) within base excision repair, damage sensor, mismatch repair, translesion synthesis and other DDR modulators. **a**) and **b**, left) Total mutation frequencies were calculated across 430 cervical carcinomas and includes missense (blue), stop codon (orange), splice site (grey) and indels (yellow). **b** and **c**, right) Frequencies of DDR aberrations including mutation (green) and homozygous deletion (blue) were calculated across 372 cervical carcinomas with overlapping GISTIC and MutSig data.

Supplementary Table 1 *Clinicopathological Characteristics across the three included patient cohorts.*

Cohorts<br>Clinicopathological characteristics	TCGA<br>n=301<br>n (%)	Ojesina *et al*<br>n=114<br>n (%)	Chung *et al*<br>n=15<br>n (%)	Total<br>n=430<br>n (%)
Median age at diagnosis	46	47	54	46.5
Histological type				
Squamous cell carcinoma	249 (83)	78 (68)	0 (0)	327 (76)
Adenocarcinoma	47 (15)	26 (24)	15 (100)	88 (20.5)
Adenosquamous carcinoma	5 (2)	8 (7)	0 (0)	13 (3)
Neuroendocrine carcinoma	0 (0)	2 (2)	0 (0)	2 (0.5)
FIGO stage				
I	159 (54)	78 (78)	7 (47)	244 (60)
II	69 (23)	18 (18)	8 (53)	95 (23)
III	46 (16)	2 (2)	0 (0)	48 (12)
IV	20 (7)	2 (2)	0 (0)	22 (5)
Grade				
Grade 1	18 (7)	6 (6)		24 (7)
Grade 2	134 (50)	54 (57)		188 (51)
Grade 3	118 (43)	35 (37)		153 (42)
Race				
Caucasian	205 (77)	114 (100)	0 (0)	319 (81)
Asian/Mongolian	30 (11.5)	0 (0)	15 (100)	45 (11)
Black	30 (11.5)	0 (0)	0 (0)	30 (8)

[a]Missing data for the TCGA cohort: Age at diagnosis: n=2, FIGO stage: n=7, Grade: n=31, Race: n= 36

[b]Missing data for the Ojesina et al cohort: Age at diagnosis and FIGO stage: n=14, Grade: n=19

[c]Missing data for the Chung et al cohort: Grade: n=15

Abbreviations: c: cohort

Supplementary Table 2 *Mutation counts and frequencies across 430 cervical carcinomas.*

Type of mutation	Count	Frequency (%)
Frame Shift Deletion	1787	1.6
Frame Shift Insertion	728	0.7
In Frame Deletion	613	0.5
In Frame Insertion	129	0.1
Missense Mutation	70060	62.6
Nonsense Mutation	6044	5.4
Nonstop Mutation	147	0.1
Silent Mutation	29491	26.4
Splice Site	2494	2.2
Translation Start Site	399	0.4
Total	***111892***	***100***

Supplementary Table 3 *Top 50 significantly mutated genes across 430 cervical carcinomas called by the MutSig2CV analysis*.*

Rank	Gene	Coding Nucleotides	Silent mutations	Mis-sense mutations	Non-sense mutations	Splice-site mutations	Indels	Non-silent mutations	Patients	Unique sites	MutFreq (% pat)	p	q
1	*EP300*	7365	4	36	20	3	5	64	52	53	12.1	1.00E-16	6.29E-13
2	*FBXW7*	2580	0	39	13	0	1	53	50	29	11.6	1.00E-16	6.29E-13
3	*KRAS*	709	0	20	0	0	0	20	20	6	4.7	1.00E-16	6.29E-13
4	*PTEN*	1244	1	17	13	3	7	40	33	31	7.7	4.77E-15	2.25E-11
5	*HLA-B*	1119	3	6	7	4	7	24	21	20	4.9	2.42E-13	9.11E-10
6	*HLA-A*	1128	2	8	6	7	3	24	24	19	5.6	9.16E-13	2.88E-09
7	*MLL3*	14968	11	47	43	4	13	107	83	99	19.3	9.51E-12	2.56E-08
8	*KRT8*	1481	1	26	0	0	0	26	25	3	5.8	2.59E-09	6.10E-06
9	*NFE2L2*	1834	1	22	1	0	1	24	20	16	4.7	3.72E-09	7.80E-06
10	*RB1*	3716	2	8	11	5	6	30	25	29	5.8	5.24E-09	9.89E-06
11	*MLL2*	16826	14	37	30	3	5	75	55	71	12.8	2.51E-08	4.31E-05
12	*MAPK1*	1115	1	18	0	0	0	18	18	5	4.2	4.13E-08	6.49E-05
13	*ARID1A*	6934	5	14	18	1	8	41	29	37	6.7	8.53E-08	1.24E-04
14	*ZNF750*	2176	3	5	4	0	7	16	15	16	3.5	1.75E-07	2.36E-04
15	*GPX1*	725	1	11	1	0	0	12	11	3	2.6	2.04E-07	2.57E-04
16	*ZC3H11A*	2493	3	10	9	0	1	20	19	9	4.4	3.17E-07	3.73E-04
17	*SMAD4*	1699	0	9	4	0	0	13	13	12	3	7.71E-07	8.56E-04
18	*DDX3X*	2053	0	13	3	0	2	18	18	18	4.2	1.78E-06	1.86E-03
19	*TGFBR2*	1807	0	6	1	2	2	11	11	10	2.6	1.99E-06	1.98E-03
20	*CASP8*	1749	3	10	9	2	2	23	21	21	4.9	2.10E-06	1.98E-03
21	*PIK3CA*	3287	0	129	0	2	2	133	118	32	27.4	2.65E-06	2.38E-03
22	*TP63*	2258	2	10	1	1	0	12	12	10	2.8	4.98E-06	4.27E-03
23	*ERBB2*	3896	4	25	0	0	1	26	21	17	4.9	5.65E-06	4.64E-03
24	*B2M*	374	0	3	3	0	2	8	8	8	1.9	8.03E-06	6.31E-03
25	*OTOP1*	1861	0	4	0	0	7	11	11	5	2.6	1.31E-05	9.88E-03
26	*ARIH1*	1726	0	0	0	0	3	3	3	1	0.7	1.49E-05	1.05E-02
27	*COIL*	1757	0	5	1	0	4	10	9	7	2.1	1.50E-05	1.05E-02
28	*NF2*	1894	2	5	5	3	1	14	14	13	3.3	1.67E-05	1.13E-02
29	*TBC1D26*	792	1	9	0	0	0	9	8	3	1.9	2.58E-05	1.68E-02
30	*RANBP2*	9787	5	24	2	1	2	29	26	23	6	3.41E-05	2.15E-02
31	*POTEC*	1669	3	23	1	1	1	26	24	13	5.6	4.14E-05	2.52E-02
33	*OR5H1*	940	2	13	0	0	0	13	8	5	1.9	6.04E-05	3.45E-02
32	*SPRED3*	1293	0	0	0	0	7	7	7	1	1.6	5.90E-05	3.45E-02
34	*FAS*	1044	1	7	0	0	1	8	8	6	1.9	6.25E-05	3.45E-02
35	*OR2J2*	940	0	1	2	0	2	5	5	3	1.2	6.40E-05	3.45E-02
36	*AKT1*	1495	0	9	0	0	0	9	9	5	2.1	7.04E-05	3.69E-02
37	*KLF5*	1386	2	10	1	0	1	12	10	7	2.3	7.42E-05	3.78E-02
38	*TRAF3*	1747	0	4	2	1	0	7	7	5	1.6	7.61E-05	3.78E-02
39	*CTNNB1*	2406	0	9	0	0	0	9	7	7	1.6	9.89E-05	4.78E-02
40	*BMS1*	3937	4	14	1	0	0	15	15	10	3.5	1.12E-04	5.29E-02
42	*DNAH12*	9554	2	5	2	1	2	10	10	9	2.3	1.25E-04	5.49E-02
41	*MSN*	1782	16	25	0	0	1	26	26	8	6	1.24E-04	5.49E-02
43	*NBPF10*	10994	23	29	4	0	2	35	33	26	7.7	1.25E-04	5.49E-02
44	*CREBBP*	7449	7	20	6	3	3	32	29	30	6.7	1.29E-04	5.53E-02
45	*ERBB3*	4266	5	19	0	0	1	20	18	16	4.2	1.50E-04	6.27E-02
46	*FAM155A*	1385	0	2	1	0	4	7	7	4	1.6	1.87E-04	7.68E-02
47	*TP53*	1890	5	23	8	0	1	32	29	28	6.7	2.02E-04	8.10E-02
48	*FAT1*	13871	11	16	15	1	8	40	35	40	8.1	2.54E-04	9.98E-02
49	*TNFRSF10C*	796	0	4	0	0	0	4	4	2	0.9	2.84E-04	1.09E-01
50	*SLC4A9*	2966	0	2	1	0	1	4	4	3	0.9	3.29E-04	1.24E-01

*Categories are extensively explained in under Supplementary Table 5.

Supplementary Table 4 *Top 50 significantly mutated genes 327 squamous cell carcinomas called by the MutSig2CV analysis*.*

Rank	Gene	Coding Nucleotides	Silent mutations	Mis-sense mutations	Non-sense mutations	Splice-site mutations	Indels	Non-silent mutations	Patients	Unique sites	MutFreq (% pat)	p	q
1	*EP300*	7365	3	32	17	2	5	56	45	47	13.8	1.00E-16	1.89E-12
2	*FBXW7*	2580	0	31	12	0	1	44	41	26	12.5	3.33E-16	3.14E-12
3	*HLA-A*	1128	2	6	6	7	3	22	22	18	6.7	7.77E-16	4.89E-12
4	*PTEN*	1244	0	11	12	3	3	29	24	24	7.3	2.26E-14	1.07E-10
5	*HLA-B*	1119	2	5	7	3	6	21	19	18	5.8	3.86E-13	1.46E-09
6	*MAPK1*	1115	1	17	0	0	0	17	17	5	5.2	5.84E-12	1.84E-08
7	*MLL3*	14968	8	35	37	4	9	85	64	78	19.6	1.96E-10	5.29E-07
8	*RB1*	3716	2	7	11	5	5	28	23	27	7	3.08E-10	7.25E-07
9	*NFE2L2*	1834	1	21	1	0	1	23	19	16	5.8	2.03E-09	4.25E-06
10	*MLL2*	16826	13	32	29	2	5	68	48	64	14.7	2.76E-09	5.20E-06
11	*CASP8*	1749	2	8	9	2	2	21	19	19	5.8	7.85E-08	1.35E-04
12	*KRT8*	1481	1	17	0	0	0	17	17	1	5.2	2.13E-07	3.35E-04
13	*KRAS*	709	0	8	0	0	0	8	8	5	2.4	2.49E-07	3.61E-04
14	*ZNF750*	2176	3	5	3	0	7	15	14	15	4.3	1.48E-06	2.00E-03
15	*TP63*	2258	1	10	1	1	0	12	12	10	3.7	1.85E-06	2.33E-03
16	*TGFBR2*	1807	0	6	1	1	2	10	10	9	3.1	7.73E-06	9.11E-03
17	*PIK3CA*	3287	0	95	0	0	2	97	87	23	26.6	8.50E-06	9.16E-03
18	*SMAD4*	1699	0	6	4	0	0	10	10	9	3.1	8.75E-06	9.16E-03
19	*OTOP1*	1861	0	3	0	0	6	9	9	4	2.8	1.04E-05	1.03E-02
20	*RANBP2*	9787	4	21	1	1	2	25	22	20	6.7	1.53E-05	1.44E-02
21	*TP53*	1890	2	13	6	0	0	19	17	15	5.2	1.86E-05	1.67E-02
22	*COIL*	1757	0	5	0	0	4	9	8	6	2.4	2.32E-05	1.99E-02
23	*KLF5*	1386	2	9	0	0	1	10	8	5	2.4	2.90E-05	2.37E-02
24	*ZC3H11A*	2493	2	7	6	0	1	14	14	8	4.3	3.55E-05	2.74E-02
25	*OR2J2*	940	0	1	2	0	2	5	5	3	1.5	3.64E-05	2.74E-02
26	*SPRED3*	1293	0	0	0	0	6	6	6	1	1.8	3.92E-05	2.84E-02
27	*GPX1*	725	1	9	1	0	0	10	9	3	2.8	4.41E-05	2.99E-02
28	*POTEC*	1669	2	18	1	1	1	21	20	10	6.1	4.44E-05	2.99E-02
29	*FAS*	1044	1	7	0	0	1	8	8	6	2.4	5.80E-05	3.78E-02
30	*DDX3X*	2053	0	8	3	0	2	13	13	13	4	6.53E-05	4.11E-02
31	*ERBB3*	4266	5	10	0	0	1	11	10	8	3.1	6.87E-05	4.18E-02
32	*B2M*	374	0	3	3	0	2	8	8	8	2.4	7.11E-05	4.19E-02
33	*SLC4A9*	2966	0	2	1	0	1	4	4	3	1.2	8.63E-05	4.93E-02
34	*C12orf43*	811	0	5	0	0	0	5	5	2	1.5	9.48E-05	5.26E-02
35	*FAT1*	13871	7	14	12	1	8	35	30	35	9.2	1.07E-04	5.78E-02
36	*BMS1*	3937	3	12	1	0	0	13	13	9	4	1.10E-04	5.79E-02
37	*MSN*	1782	14	21	0	0	0	21	21	5	6.4	1.25E-04	6.38E-02
38	*NF2*	1894	2	5	4	2	1	12	12	11	3.7	1.35E-04	6.72E-02
39	*C3orf27*	450	1	3	0	0	2	5	5	4	1.5	1.65E-04	7.92E-02
40	*BAP1*	2254	0	3	7	0	1	11	11	9	3.4	1.74E-04	7.92E-02
41	*TRIM41*	2009	0	3	1	0	3	7	7	6	2.1	1.75E-04	7.92E-02
42	*IFNGR1*	1495	0	3	7	0	0	10	9	9	2.8	1.76E-04	7.92E-02
43	*SIRPB1*	2358	0	8	0	0	0	8	8	4	2.4	2.06E-04	9.03E-02
44	*TRAF3*	1747	0	4	2	1	0	7	7	5	2.1	2.54E-04	1.09E-01
45	*MEF2A*	1696	1	7	0	0	2	9	9	8	2.8	3.13E-04	1.31E-01
46	*CGB7*	508	0	3	0	2	0	5	5	4	1.5	3.26E-04	1.34E-01
47	*USP28*	3332	1	6	1	3	0	10	10	9	3.1	4.00E-04	1.61E-01
48	*MUC17*	13532	8	37	5	0	3	45	39	42	11.9	4.78E-04	1.88E-01
49	*C6orf223*	786	0	1	0	0	2	3	3	1	0.9	5.06E-04	1.90E-01
50	*TNFRSF10C*	796	0	4	0	0	0	4	4	2	1.2	5.11E-04	1.90E-01

*Categories are extensively explained in under Supplementary Table 5.

Supplementary Table 5 *Top 30 mutated genes across 86 non-squamous cell carcinomas called by the MutSig2CV analysis*.*

Rank	Gene	Coding Nucleotides	Silent mutations	Missense mutations	Non-sense mutations	Splice-site mutations	Indels	Non-silent mutations	Patients	Unique sites	MutFreq (% pat)	p	q
1	*KRAS*	709	0	12	0	0	0	12	12	4	14.0	2.87E-13	5.42E-09
2	*ARID1A*	6934	0	3	6	0	3	12	11	11	12.8	1.53E-08	1.44E-04
3	*IL28A*	627	0	2	1	1	0	4	4	4	4.7	2.63E-06	1.65E-02
4	*TP53*	1890	1	7	1	0	1	9	8	9	9.3	4.33E-06	1.66E-02
5	*FBXW7*	2580	0	6	1	0	0	7	7	7	8.1	4.40E-06	1.66E-02
6	*MLL3*	14968	2	9	5	0	4	18	16	18	18.6	9.20E-06	2.89E-02
7	*PIK3CA*	3287	0	25	0	2	0	27	22	12	25.6	1.35E-05	3.64E-02
8	*PTEN*	1244	1	3	0	0	4	7	6	7	7.0	2.77E-05	6.53E-02
9	*AKT1*	1495	0	4	0	0	0	4	4	3	4.7	4.64E-05	9.67E-02
10	*ZC3H11A*	2493	0	3	3	0	0	6	5	3	5.8	5.13E-05	9.67E-02
11	*ERBB2*	3896	0	9	0	0	0	9	8	7	9.3	9.73E-05	1.67E-01
12	*EP300*	7365	1	1	3	1	0	5	4	5	4.7	1.53E-04	2.40E-01
13	*KRT8*	1481	0	3	0	0	0	3	3	1	3.5	4.87E-04	7.07E-01
14	*CBFB*	615	0	1	0	0	1	2	2	2	2.3	9.65E-04	1
15	*SOX17*	1249	0	1	0	0	1	2	2	2	2.3	1.14E-03	1
16	*TGM4*	2109	0	0	1	1	0	2	2	2	2.3	1.22E-03	1
17	*ATXN3*	1128	0	0	0	0	3	3	2	2	2.3	1.37E-03	1
18	*ELF3*	1148	1	2	1	0	3	6	6	6	7.0	1.54E-03	1
19	*POLM*	1527	0	2	0	1	1	4	3	4	3.5	1.65E-03	1
20	*PODXL*	1709	0	0	0	0	2	2	2	2	2.3	2.57E-03	1
21	*HSH2D*	1082	0	2	1	0	0	3	2	3	2.3	2.80E-03	1
22	*C21orf58*	1005	0	1	0	0	2	3	3	2	3.5	3.02E-03	1
23	*CCDC33*	2603	0	3	0	0	0	3	3	3	3.5	3.13E-03	1
24	*DNAH12*	9554	0	0	1	1	1	3	3	3	3.5	3.57E-03	1
25	*ABHD4*	1055	0	0	2	0	0	2	2	1	2.3	3.95E-03	1
26	*GPR110*	2837	0	1	1	0	0	2	2	1	2.3	4.16E-03	1
27	*ZFP36L1*	1198	0	1	1	0	0	2	2	2	2.3	4.20E-03	1
28	*LAMTOR4*	314	0	0	0	1	1	2	2	2	2.3	4.40E-03	1
29	*MBNL1*	1685	0	1	2	0	0	3	2	3	2.3	4.58E-03	1
30	*DNASE1L1*	1226	0	2	0	0	0	2	2	1	2.3	4.91E-03	1

*Extended category explanation:

Coding nucleotides: number of coding nucleotides within this gene covered by sequencing
Silent mutations: number of silent mutations in this gene across the individual set
Missense mutation: number of missense mutations in this gene across the individual set
Non-sense mutations: number of nonsense mutations in this gene across the individual set
Splice site mutations: number of splice site mutations in this gene across the individual set
Indel: number of indel mutations in this gene across the individual set
Non-silent mutations: number of (non-silent) mutations in this gene across the individual set
Patients: number of patients (individuals) with at least one non-silent mutation
Unique sites: number of unique sites having a non-silent mutation
p: p-value (overall)
q: q-value, False Discovery Rate (Benjamini-Hochberg procedure)

Supplementary Table 6 *Mutations in chromatin remodeling families or genes.*

Gene/Family	***Family members mutated***	***# patients with mutation***	***Frequency (%)***
MLL family	*MLL, MLL2, MLL3*	119	27.7
KDM Family	*KDM1A, KDM1B, KDM2A, KDM2B, KDM3A, KDM3B, KDM4A, KDM4B, KDM4C, KDM4D, KDM4DL, KDM5A, KDM5B, KDM5C, KDM5D, KDM6A, KDM6B*	94	21.9
ARID Family	*ARID1A, ARID1B, ARID2, ARID3A, ARID3B, ARID3C, ARID4A, ARID4B ARID5A, ARID5B*	59	13.7
EP300		52	12.1
SMARC Family	*SMARCA1, SMARCA2, SMARCAD1, SMARCC2, SMARCD1, SMARCD3, SMARCE1*	35	8.1
DNMT Family	*DNMT1, DNMT3A, DNMT3B, DNMT3L*	22	5.1
HIST Family	*HIST1H1A, HIST1H1B, HIST1H1C, HIST1H1D, HIST1H1E, HIST1H1T*	16	3.7
PBRM1		9	2.1
**# samples with mutated chromatin modifiers**		**228**	**53.0**
**Total samples**		**430**	**100.0**

Supplementary Table 7 *Novel focal amplifications (**a**) and deletions (**b**) across 430 cervical carcinomas.*

Supplementary Table 7a Focal copy number amplifications				
Cytoband	***Genomic region***	***Putative driver gene(s)***	***Comment***	***Reference(s)***
**1p31.1**	chr1:61831890 - 61932604	*NFIA*		(1)
**1q21.3**	chr1:151300377 - 151331927	*RFX5*	Transcriptional activator in hepatocellular carcinoma	(2, 3)
**4q12**	chr4:56093585 - 56222716	*SRD5A3*	Overexpressed in prostate cancer.	(4)
**6p21.33**	chr6:30526845 - 30526845	*GNL1*	GNL1 is a nucleolar GTPase, which promotes cell cycle and proliferation by inducing hyperphosphorylation of retinoblastoma protein.	(5)
**18p11.31**	chr18:3155700 - 3254846	*TGIF1*		(6, 7)
**19q13.13**	chr19:38373478 - 38390659	*WDR87*	*Manuscript in preparation.*	
**19q13.2**	chr19:3383496 - 3401817	*NFIC*		(8)
**Xq28**	chrX:153212816 - 153254952	*HCFC1, TMEM187*	HCFC1 is overexpressed in multiple tumors	(9)

Supplementary Table 7b Focal copy number deletions				
Cytoband	***Genomic region***	***Putative driver gene(s)***	***Comment***	***References(s)***
**2q37.1**	chr2:233034495 - 233048132	*MIR562*	The putative role of miR-562 as the tumor suppressor in the 2q37.1 deletion peak has been previously proposed in the context of Wilms' tumors.	(10)
		DIS3L2	DISA3L2 loss fosters susceptibility to Wilms tumor and its overexpression is associated with decreased cellular proliferation. Tumor suppressive in colon cancer, inversely proportional to cell cycle inhibitors p27 and p21, and knockdown promotes cellular proliferation.	(11, 12)
**4q22.1**	chr4:91039783 - 92523213	*CCSER1*	Patel et al demonstrated that CCSER1 (aka FAM190A) deficient is associated with a cell division defect.	(13)
**5q12.1**	chr5:58261529-59818040	*PDE4D*	Although thought to have pro-cancer properties, PDE4D deletions have been observed in multiple tumor types	(14, 15)
**6p25.3**	chr6:1309918 - 1315309	*FOXQ1*	Possibly paradoxical; FOXQ1 has oncogenic properties in several cancer types	(16)
**6q26**	chr6:163145416 - 163736814	*PACRG, PARK2*		(17, 18)
**8p23.2**	chr8:2792739 - 4852925	*CSMD1*		(19, 20)
**11q14.2**	chr11:85803238 - 85805593	*EED*	EED is a context-dependent tumor-suppressor gene in KRAS-driven lung cancer.	(21)
		PICALM	PICALM deficiency increases cholesterol biosynthesis.	(22)
**11q23.3**	chr11:85803238 - 85805593	*PVRL1*	PVRL1 is a metastasis suppressor gene in melanoma.	(23)
**11q25**	chr11:131239804 - 132208428	*NTM*	NTM belongs to the IgLON family of GPI-anchored cell adhesion molecules, which is part of the immunoglobulin (Ig) domain-containing superfamily. The IgLON family gets its name from the 3 members LSAMP, OPCML, and neurotrimin (NTM).	(24)
**14q32.2**	chr14:100148237 - 100195717	*CYP46A1*	CYP46A1 is a cholesterol-regulatory gene with putative tumor suppressor function in glioblastoma.	(25)
**15q15.1**	chr15:42138456 - 42186351	*MIR4310*	Negative transcriptional regulator of MMP-10, which promotes cervical cancer progression.	(26, 27)
**16q11.2**	chr16:31850299 - 46615362	*ZNF267, TP53TG3, TP53TG3B TP53TG3C TP53TG3D*	Induced by TP53.	(28)
**Xp11.3**	chrX:44729855 - 44975013	*KDM6A*	Inactivated in multiple cancers.	(29-31)

Supplementary Table 8 *Significantly enriched MSigDB Curated (c2), Gene Ontology (c5) and Hallmarks (H) gene sets within amplified and mutated oncogenes in the whole cohort.*

Significantly enriched Curated gene sets (c2)	***P-value***	***FDR q-value***
KEGG_PATHWAYS_IN_CANCER	1.05E-20	5.83E-17
KEGG_ENDOMETRIAL_CANCER	2.30E-19	6.37E-16
KEGG_ERBB_SIGNALING_PATHWAY	5.61E-17	9.84E-14
KEGG_PROSTATE_CANCER	7.12E-17	9.84E-14
BIOCARTA_TFF_PATHWAY	2.82E-15	3.12E-12
REACTOME_SIGNALING_BY_ERBB2	5.33E-15	4.24E-12
KEGG_FOCAL_ADHESION	5.37E-15	4.24E-12
KEGG_NON_SMALL_CELL_LUNG_CANCER	1.03E-14	7.10E-12
REACTOME_CYTOKINE_SIGNALING_IN_IMMUNE_SYSTEM	4.43E-14	2.66E-11
KEGG_GLIOMA	4.93E-14	2.66E-11
REACTOME_NEGATIVE_REGULATION_OF_THE_PI3K_AKT_NETWORK	5.29E-14	2.66E-11
BIOCARTA_TEL_PATHWAY	7.96E-14	3.67E-11
KEGG_PANCREATIC_CANCER	9.16E-14	3.90E-11
KEGG_CHRONIC_MYELOID_LEUKEMIA	1.30E-13	5.14E-11
PID_ERBB2_ERBB3_PATHWAY	3.06E-13	1.13E-10
KEGG_SMALL_CELL_LUNG_CANCER	4.17E-13	1.44E-10
BIOCARTA_HER2_PATHWAY	6.45E-13	2.10E-10
REACTOME_SIGNALING_BY_ERBB2_IN_CANCER	1.47E-12	4.50E-10
KEGG_ACUTE_MYELOID_LEUKEMIA	2.08E-12	6.05E-10
KEGG_COLORECTAL_CANCER	3.85E-12	1.06E-09
Significantly enriched Gene Ontology gene sets (c5)	***P-value***	***FDR q-value***
GO_POSITIVE_REGULATION_OF_CELL_POPULATION_PROLIFERATION	1.04E-15	1.06E-11
GO_NEGATIVE_REGULATION_OF_CELL_DEATH	6.45E-14	2.32E-10
GO_REGULATION_OF_CELL_POPULATION_PROLIFERATION	6.82E-14	2.32E-10
GO_REGULATION_OF_CELL_DEATH	9.48E-14	2.41E-10
GO_POSITIVE_REGULATION_OF_MULTICELLULAR_ORGANISMAL_PROCESS	2.04E-13	4.04E-10
GO_TUBE_DEVELOPMENT	2.38E-13	4.04E-10
GO_REGULATION_OF_IMMUNE_SYSTEM_PROCESS	5.95E-13	8.66E-10
GO_POSITIVE_REGULATION_OF_RNA_METABOLIC_PROCESS	8.85E-13	1.13E-09
GO_REGULATION_OF_INTRACELLULAR_SIGNAL_TRANSDUCTION	3.70E-12	3.65E-09
GO_POSITIVE_REGULATION_OF_SIGNALING	3.91E-12	3.65E-09
GO_POSITIVE_REGULATION_OF_NUCLEOBASE_CONTAINING_COMPOUND_METABOLIC_PROCESS	3.94E-12	3.65E-09
GO_TUBE_MORPHOGENESIS	4.73E-12	4.01E-09
GO_POSITIVE_REGULATION_OF_CELLULAR_BIOSYNTHETIC_PROCESS	1.09E-11	8.53E-09
GO_TRANSCRIPTION_FACTOR_BINDING	2.64E-11	1.92E-08
GO_POSITIVE_REGULATION_OF_DEVELOPMENTAL_PROCESS	1.15E-10	7.82E-08
GO_POSITIVE_REGULATION_OF_TRANSCRIPTION_BY_RNA_POLYMERASE_II	1.37E-10	8.75E-08
GO_APOPTOTIC_SIGNALING_PATHWAY	1.74E-10	1.05E-07
GO_RESPONSE_TO_ABIOTIC_STIMULUS	2.04E-10	1.16E-07
GO_POSITIVE_REGULATION_OF_INTRACELLULAR_SIGNAL_TRANSDUCTION	2.66E-10	1.43E-07
GO_CELLULAR_RESPONSE_TO_DNA_DAMAGE_STIMULUS	4.65E-10	2.37E-07
Significantly enriched Hallmark gene sets (H)	***P-value***	***FDR q-value***
HALLMARK_APOPTOSIS	3.42E-09	1.71E-07
HALLMARK_TNFA_SIGNALING_VIA_NFKB	1.54E-08	3.84E-07
HALLMARK_PI3K_AKT_MTOR_SIGNALING	1.62E-05	2.69E-04
HALLMARK_UV_RESPONSE_DN	5.57E-05	6.97E-04
HALLMARK_IL2_STAT5_SIGNALING	1.94E-04	1.41E-03
HALLMARK_APICAL_JUNCTION	1.98E-04	1.41E-03
HALLMARK_ESTROGEN_RESPONSE_EARLY	1.98E-04	1.41E-03
HALLMARK_WNT_BETA_CATENIN_SIGNALING	1.67E-03	1.04E-02
HALLMARK_TGF_BETA_SIGNALING	2.75E-03	1.15E-02
HALLMARK_ALLOGRAFT_REJECTION	2.99E-03	1.15E-02
HALLMARK_G2M_CHECKPOINT	2.99E-03	1.15E-02
HALLMARK_GLYCOLYSIS	2.99E-03	1.15E-02
HALLMARK_KRAS_SIGNALING_UP	2.99E-03	1.15E-02
HALLMARK_IL6_JAK_STAT3_SIGNALING	6.97E-03	2.49E-02
HALLMARK_PROTEIN_SECRETION	8.42E-03	2.81E-02
HALLMARK_ANDROGEN_RESPONSE	9.11E-03	2.85E-02

Supplementary Table 9 *Significantly enriched MSigDB Curated (c2), Gene Ontology (c5) and Hallmarks (H) gene sets within deleted and mutated tumor suppressors within the whole cohort.*

Significantly enriched Curated gene sets (c2)	**P-value**	**FDR q-value**
GRESHOCK_CANCER_COPY_NUMBER_UP	2.10E-14	1.16E-10
REACTOME_RNA_POLYMERASE_II_TRANSCRIPTION	2.50E-12	6.90E-09
KEGG_PATHWAYS_IN_CANCER	3.59E-10	6.61E-07
TAKADA_GASTRIC_CANCER_COPY_NUMBER_DN	1.54E-09	2.12E-06
BIOCARTA_FAS_PATHWAY	2.22E-09	2.46E-06
PID_P53_DOWNSTREAM_PATHWAY	5.65E-09	5.21E-06
LIN_MELANOMA_COPY_NUMBER_DN	1.15E-08	9.09E-06
BIOCARTA_PML_PATHWAY	2.23E-08	1.54E-05
REACTOME_CHROMATIN_MODIFYING_ENZYMES	3.48E-08	1.93E-05
BIOCARTA_TGFB_PATHWAY	3.62E-08	1.93E-05
DACOSTA_UV_RESPONSE_VIA_ERCC3_DN	3.83E-08	1.93E-05
BIOCARTA_NTHI_PATHWAY	8.23E-08	3.79E-05
REACTOME_ACTIVATION_OF_ANTERIOR_HOX_GENES_IN_HINDBRAIN_DEVELOPMENT_DURING_EARLY_EMBRYOGENESIS	9.24E-08	3.90E-05
BIOCARTA_CTCF_PATHWAY	9.87E-08	3.90E-05
KUROKAWA_LIVER_CANCER_EARLY_RECURRENCE_DN	1.11E-07	4.08E-05
DING_LUNG_CANCER_MUTATED_SIGNIFICANTLY	1.38E-07	4.78E-05
KEGG_ADHERENS_JUNCTION	2.21E-07	7.18E-05
DER_IFN_ALPHA_RESPONSE_UP	2.36E-07	7.26E-05
REACTOME_DISEASE	2.93E-07	8.19E-05
TCGA_GLIOBLASTOMA_MUTATED	3.09E-07	8.19E-05
Significantly enriched Gene Ontology gene sets (c5)	***P-value***	***FDR q-value***
GO_PEPTIDYL_AMINO_ACID_MODIFICATION	6.18E-11	5.38E-07
GO_INTRINSIC_APOPTOTIC_SIGNALING_PATHWAY_BY_P53_CLASS_MEDIATOR	1.06E-10	5.38E-07
GO_NEGATIVE_REGULATION_OF_SIGNALING	4.28E-10	1.45E-06
GO_APOPTOTIC_SIGNALING_PATHWAY	7.87E-10	2.00E-06
GO_SIGNAL_TRANSDUCTION_BY_P53_CLASS_MEDIATOR	1.31E-09	2.66E-06
GO_GROWTH	2.17E-09	3.46E-06
GO_REGULATION_OF_CELLULAR_RESPONSE_TO_TRANSFORMING_GROWTH_FACTOR_BETA_STIMULUS	2.37E-09	3.46E-06
GO_DEVELOPMENTAL_GROWTH	2.88E-09	3.67E-06
GO_REGULATION_OF_PROTEIN_MODIFICATION_PROCESS	3.80E-09	3.67E-06
GO_REGULATION_OF_CELL_DIFFERENTIATION	3.83E-09	3.67E-06
GO_NEGATIVE_REGULATION_OF_TRANSCRIPTION_BY_RNA_POLYMERASE_II	3.96E-09	3.67E-06
GO_CELLULAR_RESPONSE_TO_ENDOGENOUS_STIMULUS	4.73E-09	4.01E-06
GO_TRANSCRIPTION_COREGULATOR_ACTIVITY	5.66E-09	4.03E-06
GO_NEGATIVE_REGULATION_OF_RESPONSE_TO_STIMULUS	6.09E-09	4.03E-06
GO_POSITIVE_REGULATION_OF_RNA_METABOLIC_PROCESS	6.47E-09	4.03E-06
GO_TRANSCRIPTION_COACTIVATOR_ACTIVITY	6.86E-09	4.03E-06
GO_TRANSCRIPTION_REGULATOR_ACTIVITY	6.94E-09	4.03E-06
GO_RESPONSE_TO_ENDOGENOUS_STIMULUS	7.12E-09	4.03E-06
GO_HEART_DEVELOPMENT	8.22E-09	4.40E-06
GO_REGULATION_OF_CELL_DEATH	8.96E-09	4.40E-06
Significantly enriched Hallmark gene sets (H)	***P-value***	***FDR q-value***
HALLMARK_INTERFERON_GAMMA_RESPONSE	3.12E-05	7.81E-04
HALLMARK_P53_PATHWAY	3.12E-05	7.81E-04
HALLMARK_WNT_BETA_CATENIN_SIGNALING	2.61E-03	3.87E-02
HALLMARK_APOPTOSIS	3.09E-03	3.87E-02
HALLMARK_ALLOGRAFT_REJECTION	5.66E-03	4.05E-02
HALLMARK_APICAL_JUNCTION	5.66E-03	4.05E-02
HALLMARK_E2F_TARGETS	5.66E-03	4.05E-02

Supplementary Table 10 *Focal copy number amplifications across 290 squamous cell carcinoma (**a**) and 69 non-squamous cell carcinomas (**b**).*

Supplementary Table 10a Squamous cell carcinoma				
Cytoband	*Genomic region*	*Putative driver gene(s)*	*Comment*	*Reference(s)*
**1q44**	chr1:244167131-244243575	*AKT3*		(32)
**3q28**	chr3:189647767-189660321	*TP63*		(33)
**4q12**	chr4:56093585-56222716	*SRD5A3*	Overexpressed in prostate cancer.	(4)
**7p11.2**	chr7:55054805-55094029	*EGFR*		(34, 35)
**9p24.1**	chr9:5341593-5423103	*CD274, PLGRKT, PDCD1LG2*		(36, 37)
**11p12**	chr11:36320780-36962794	*TRAF6*		(38)
**11q13.3**	chr11:70189517-70370188	*CTTN, PPFIA1*	CTTN is an amplification core in oral SCC correlated with PPFIA1.	(39)
**11q22.1**	chr11:101894838-101984747	*YAP1, BIRC2, BIRC3*		(40)
**12p12.1**	chr12:25240140-25784001	*KRAS*		(41, 42)
**13q22.1**	chr13:73953432-73954914	*LINC00393, KLF5*		(43, 44)
**17q25.1**	chr17:73270906-73289296	*GRB2*		(45)
**18p11.31**	chr18:3155700-3254846	*TGIF1*		(6, 7)
**19q13.13**	chr19:38373478-38390659	*WDR87*	*Manuscript in preparation.*	
**19q13.32**	chr19:45253104-45256035	*BCL3*		(46)
**21q22.13**	chr21:38470347-38682147	*VPS26C/DSCR3*	VPS26C/DSCR3 is a member of the Retriever complex for endosomal cargo cycling, required for HPV viral DNA to reach the nucleus.	(47)

Supplementary Table 10b Non-squamous cell carcinoma				
Cytoband	*Genomic Region*	*Putative driver gene(s)*	*Comment*	*Reference(s)*
**3q26.2**	chr3:191370766-191372559	*PYDC2*	Regulates inflammasome activation.	(48)
**7q36.3**	chr7:157696405-157700614	*PTPRN2*	Tyrosine phosphatase receptor that promotes metastasis in breast cancer through actin remodeling.	(49)
**19q12**	chr19:29309499-30307451	*CCNE1, PLEKHF1, POP4*	Inhibition of POP4, PLEKHF1, CCNE1 and TSZH3 lowers cell viability in cancer cells harboring their amplification.	(50)
			POP4 represses H3.3 chromatin assembly and promotes H3K9me3 and H3K27me3.	(51)

Supplementary Table 11 *Significantly enriched MSigDB Curated (c2), Gene Ontology (c5) and Hallmarks (H) gene sets within amplified and mutated SCC oncogenes.*

Significantly enriched Curated gene sets (c2)	**P-value**	**FDR q-value**
KEGG_ERBB_SIGNALING_PATHWAY	1.75E-13	6.36E-10
KEGG_PATHWAYS_IN_CANCER	2.30E-13	6.36E-10
KEGG_ENDOMETRIAL_CANCER	3.87E-13	7.12E-10
SNIJDERS_AMPLIFIED_IN_HEAD_AND_NECK_TUMORS	6.22E-12	8.60E-09
REACTOME_SIGNALING_BY_ERBB2	4.20E-11	4.65E-08
REACTOME_SHC1_EVENTS_IN_ERBB2_SIGNALING	6.38E-11	5.35E-08
KEGG_NON_SMALL_CELL_LUNG_CANCER	6.80E-11	5.35E-08
BIOCARTA_HER2_PATHWAY	8.14E-11	5.35E-08
REACTOME_NEGATIVE_REGULATION_OF_THE_PI3K_AKT_NETWORK	8.71E-11	5.35E-08
KEGG_DORSO_VENTRAL_AXIS_FORMATION	1.03E-10	5.68E-08
REACTOME_SIGNALING_BY_ERBB2_IN_CANCER	1.59E-10	7.98E-08
CHARAFE_BREAST_CANCER_LUMINAL_VS_BASAL_DN	2.12E-10	9.75E-08
KEGG_PANCREATIC_CANCER	3.40E-10	1.45E-07
DUTTA_APOPTOSIS_VIA_NFKB	4.08E-10	1.50E-07
PID_CD40_PATHWAY	4.08E-10	1.50E-07
KEGG_CHRONIC_MYELOID_LEUKEMIA	4.40E-10	1.52E-07
REACTOME_CYTOKINE_SIGNALING_IN_IMMUNE_SYSTEM	4.77E-10	1.55E-07
KEGG_SMALL_CELL_LUNG_CANCER	1.04E-09	3.20E-07
KEGG_PROSTATE_CANCER	1.48E-09	4.31E-07
KEGG_BLADDER_CANCER	2.02E-09	5.40E-07
Significantly enriched Gene Ontology gene sets (c5)	**P-value**	**FDR q-value**
GO_POSITIVE_REGULATION_OF_CELL_POPULATION_PROLIFERATION	1.04E-15	1.06E-11
GO_NEGATIVE_REGULATION_OF_CELL_DEATH	6.45E-14	2.32E-10
GO_REGULATION_OF_CELL_POPULATION_PROLIFERATION	6.82E-14	2.32E-10
GO_REGULATION_OF_CELL_DEATH	9.48E-14	2.41E-10
GO_POSITIVE_REGULATION_OF_MULTICELLULAR_ORGANISMAL_PROCESS	2.04E-13	4.04E-10
GO_TUBE_DEVELOPMENT	2.38E-13	4.04E-10
GO_REGULATION_OF_IMMUNE_SYSTEM_PROCESS	5.95E-13	8.66E-10
GO_POSITIVE_REGULATION_OF_RNA_METABOLIC_PROCESS	8.85E-13	1.13E-09
GO_REGULATION_OF_INTRACELLULAR_SIGNAL_TRANSDUCTION	3.70E-12	3.65E-09
GO_POSITIVE_REGULATION_OF_SIGNALING	3.91E-12	3.65E-09
GO_POSITIVE_REGULATION_OF_NUCLEOBASE_CONTAINING_COMPOUND_METABOLIC_PROCESS	3.94E-12	3.65E-09
GO_TUBE_MORPHOGENESIS	4.73E-12	4.01E-09
GO_POSITIVE_REGULATION_OF_CELLULAR_BIOSYNTHETIC_PROCESS	1.09E-11	8.53E-09
GO_TRANSCRIPTION_FACTOR_BINDING	2.64E-11	1.92E-08
GO_POSITIVE_REGULATION_OF_DEVELOPMENTAL_PROCESS	1.15E-10	7.82E-08
GO_POSITIVE_REGULATION_OF_TRANSCRIPTION_BY_RNA_POLYMERASE_II	1.37E-10	8.75E-08
GO_APOPTOTIC_SIGNALING_PATHWAY	1.74E-10	1.05E-07
GO_RESPONSE_TO_ABIOTIC_STIMULUS	2.04E-10	1.16E-07
GO_POSITIVE_REGULATION_OF_INTRACELLULAR_SIGNAL_TRANSDUCTION	2.66E-10	1.43E-07
GO_CELLULAR_RESPONSE_TO_DNA_DAMAGE_STIMULUS	4.65E-10	2.37E-07
Significantly enriched Hallmark gene sets (H)	**P-value**	**FDR q-value**
HALLMARK_TNFA_SIGNALING_VIA_NFKB	1.89E-07	9.46E-06
HALLMARK_APOPTOSIS	1.80E-06	4.50E-05
HALLMARK_UV_RESPONSE_DN	3.24E-05	5.41E-04
HALLMARK_APICAL_JUNCTION	1.16E-04	1.16E-03
HALLMARK_G2M_CHECKPOINT	1.16E-04	1.16E-03
HALLMARK_PI3K_AKT_MTOR_SIGNALING	3.14E-04	2.62E-03
HALLMARK_PROTEIN_SECRETION	6.47E-03	4.62E-02

Supplementary Table 12 *Significantly enriched MSigDB Curated (c2), Gene Ontology (c5) and Hallmarks (H) gene sets within amplified and mutated non-SCC oncogenes.*

Significantly enriched Curated gene sets (c2)	**P-value**	**FDR q-value**
KEGG_PATHWAYS_IN_CANCER	2.06E-10	7.85E-07
LIN_MELANOMA_COPY_NUMBER_UP	2.84E-10	7.85E-07
CLIMENT_BREAST_CANCER_COPY_NUMBER_UP	4.70E-10	8.67E-07
CHIN_BREAST_CANCER_COPY_NUMBER_UP	9.31E-10	1.29E-06
REACTOME_INTERLEUKIN_4_AND_INTERLEUKIN_13_SIGNALING	2.76E-09	3.05E-06
REACTOME_CYTOKINE_SIGNALING_IN_IMMUNE_SYSTEM	5.97E-09	5.50E-06
KEGG_ENDOMETRIAL_CANCER	1.42E-08	1.13E-05
KEGG_PANCREATIC_CANCER	4.80E-08	3.12E-05
KEGG_MELANOMA	5.08E-08	3.12E-05
KEGG_CHRONIC_MYELOID_LEUKEMIA	5.69E-08	3.14E-05
KEGG_SMALL_CELL_LUNG_CANCER	1.00E-07	5.05E-05
REACTOME_PI3K_AKT_SIGNALING_IN_CANCER	2.20E-07	1.01E-04
REACTOME_NEGATIVE_REGULATION_OF_THE_PI3K_AKT_NETWORK	2.98E-07	1.27E-04
PID_CD40_PATHWAY	5.38E-07	2.12E-04
PID_IL2_PI3K_PATHWAY	7.15E-07	2.64E-04
REACTOME_DISEASES_OF_SIGNAL_TRANSDUCTION_BY_GROWTH_FACTOR_RECEPTORS_AND_SECOND_MESSENGERS	1.17E-06	3.82E-04
KEGG_JAK_STAT_SIGNALING_PATHWAY	1.18E-06	3.82E-04
NIKOLSKY_BREAST_CANCER_11Q12_Q14_AMPLICON	1.27E-06	3.90E-04
KEGG_BLADDER_CANCER	1.37E-06	3.98E-04
PID_IL6_7_PATHWAY	1.93E-06	5.25E-04
Significantly enriched Gene Ontology gene sets (c5)	**P-value**	**FDR q-value**
GO_POSITIVE_REGULATION_OF_CELL_POPULATION_PROLIFERATION	1.43E-08	1.46E-04
GO_POSITIVE_REGULATION_OF_EPITHELIAL_CELL_PROLIFERATION	6.44E-08	3.19E-04
GO_CYTOKINE_MEDIATED_SIGNALING_PATHWAY	9.40E-08	3.19E-04
GO_REGULATION_OF_CELL_POPULATION_PROLIFERATION	1.22E-06	3.10E-03
GO_RESPONSE_TO_CYTOKINE	1.64E-06	3.34E-03
GO_REGULATION_OF_INTRACELLULAR_SIGNAL_TRANSDUCTION	2.25E-06	3.34E-03
GO_POSITIVE_REGULATION_OF_SIGNALING	2.31E-06	3.34E-03
GO_EPITHELIAL_CELL_PROLIFERATION	2.63E-06	3.34E-03
GO_RESPONSE_TO_UV_A	3.86E-06	4.37E-03
GO_POSITIVE_REGULATION_OF_TRANSPORT	7.94E-06	8.08E-03
GO_REGULATION_OF_RESPONSE_TO_STRESS	8.72E-06	8.08E-03
GO_POSITIVE_REGULATION_OF_INTRACELLULAR_SIGNAL_TRANSDUCTION	1.01E-05	8.17E-03
GO_REGULATION_OF_PROTEIN_LOCALIZATION	1.07E-05	8.17E-03
GO_PROTEIN_KINASE_B_SIGNALING	1.12E-05	8.17E-03
GO_RESPONSE_TO_OXYGEN_CONTAINING_COMPOUND	1.52E-05	1.03E-02
GO_RESPONSE_TO_ABIOTIC_STIMULUS	2.87E-05	1.77E-02
GO_RESPONSE_TO_GROWTH_FACTOR	3.03E-05	1.77E-02
GO_POSITIVE_REGULATION_OF_CELL_DEATH	3.13E-05	1.77E-02
GO_REGULATION_OF_CELLULAR_RESPONSE_TO_STRESS	3.43E-05	1.84E-02
GO_NEGATIVE_REGULATION_OF_RELEASE_OF_CYTOCHROME_C_FROM_MITOCHONDRIA	4.38E-05	2.23E-02
Significantly enriched Hallmark gene sets (H)	**P-value**	**FDR q-value**
HALLMARK_APOPTOSIS	7.82E-05	3.91E-03
HALLMARK_ANDROGEN_RESPONSE	1.24E-03	3.09E-02
HALLMARK_UV_RESPONSE_DN	2.54E-03	3.44E-02
HALLMARK_IL2_STAT5_SIGNALING	4.77E-03	3.44E-02
HALLMARK_ESTROGEN_RESPONSE_EARLY	4.82E-03	3.44E-02
HALLMARK_G2M_CHECKPOINT	4.82E-03	3.44E-02
HALLMARK_TNFA_SIGNALING_VIA_NFKB	4.82E-03	3.44E-02

Supplementary Table 13 *Focal copy number deletions in 290 squamous cell carcinomas (**a**) and 69 non-squamous cell carcinomas (**b**).*

***Supplementary Table 13a* Squamous cell carcinoma**				
Cytoband	***Genomic region***	***Putative driver gene(s)***	***Comment***	***Reference(s)***
**1p36.22**	chr1:10531619-10694040	*PEX14*		(52)
**1p13.2**	chr1:112912722-113195407	*ST7L*		(53)
**2q37.1**	chr2:233514309-233654381	*EFHD1, GIGYF2*	EFHD1 has putative tumor suppressive properties in renal cell carcinoma. GIGYF2 is a translational repressor.	(54, 55)
**3p14.2**	chr3:59702821-61237398	*FHIT*		(56)
**4q21.3**	chr4:87511571-87737413	*PTPN13*	Loss is associated with HPV-induced oncogenic cervical transformation, and expression is restored by curcumin treatment of cervical cancer cells.	(57, 58)
**4q22.1**	chr4:91039783-92523213	*CCSER1*	Patel et al demonstrated that CCSER1 (aka FAM190A) deficient is associated with a cell division defect.	(13)
**5q11.2**	chr5:52391230-52416203	*MOCS2, ITGA2*	Silencing of ITGA2 stimulates breast cancer cell migration	(59)
**6p24**	chr6:11077198-11084637	*ELOVL2*	Tumor suppressive in breast cancer and neuroblastoma.	(60, 61)
**6q27**	chr6:168331594-168862574	*KIF25, FRMD1, DACT2*	DACT2 is a tumor suppressor in colorectal cancer.	(62)
**8p23.1**	chr8:11278600-11332602	*FAM167A*		
**8p23.2**	chr8:2792739-4852925	*CSMD1*		(19)
**9p24.2**	chr9:2720219-2847114	*KIAA0020, KCNV2*		(63)
**11q25**	chr11:133766301-133768886	*MIR4697*		
**13q12.11**	chr13:19581842-19586754	*LINC00442*		
**13q14.11**	chr13:41482177-42387731	*ELF1, KBTBD6, KBTBD7*	ELF1 is downregulated in prostate cancer. Decreased in metastasis and inhibits EMT. *KBTBD6/KBTBD7 d*epletion is associated with increased invasive potential.	(64, 65)
**13q14.2**	chr13:50105055-50160945	*RCBTB1*	Deletion is associated with sarcoma metastasis and docetaxel resistance.	(66)
**16q12.1**	chr16:50903329-50904271	*CYLD*	CYLD loss of function mutations are common in HPV-positive cylindroma-like basaloid carcinomas of the anus.	(67)
**16p13.3**	chr16:762953-797786	*NARFL*	Cellular defense against hyperoxia-induced loss of sister chromatid cohesion.	(68)
**17q25.3**	chr17:80917016-80917016	*METRNL*	METRNL regulates immune-adipose interactions and insulin resistance.	(69)
		FOXK2, ZNF750	FOXK2 is a transcriptional repressor, which inhibits hypoxic response and suppresses breast cancer proliferation and invasion.	(70, 71)
**18q23**	chr18:74240503-74326105	*LINC00908*		(72)
**19q13.43**	chr19:56296582-56348438	*NLRP11*	Targets TRAF6 for ubiquitination.	(73)
**Xp11.3**	chrX:44729855-44975013	*KDM6A*	Inactivated in multiple cancers.	(29-31)
**Xq21.33**	chrX:95939469-96860054	*RPA4*	Replication factor essential for DNA repair. RPA4 expression is decreased in cancerous tissues.	(74, 75)

Supplementary Table 13b Non-squamous carcinoma				
Cytoband	***Genomic Region***	***Putative driver gene(s)***	***Comment***	***Reference(s)***
**1p36.32**	chr1:3218610-3400790	*TP73, PRDM16*	Suppresses lung adenocarcinoma metastasis.	(76)
**2q37.3**	chr2:238534955-238724424	*LRRFIP1*	Paradoxical; suggested to have oncogenic properties	(77)
**4q21.3**	chr4:87460995-87466631	*MIR4452*		

**11q23.3**	chr11:117947433-119604854	*PVRL1*	PVRL1 is a metastasis suppressor gene in melanoma.	(23)
**14q32.31**	chr14:102225526-102395370	*PPP2R5C*	Dephosphorylates and activates p53.	(78)
**15q21.1**	chr15:45694421-45720715	*SPATA5L1*		
**16q12.2**	chr16:54952032-54978787	*IRX5*	IRX5 deficiency facilitates growth of Wilms' tumors.	(79)
**18q21.2**	chr18:48492168-48616656	*SMAD4*	Smad4/DPC4-mediated tumor suppression through suppression of angiogenesis.	(80)
**Xp22.31**	chrX:9101276-9103093	*MIR4707, MIR4767*	Depletion through lncRNA sponging leads to suppression of apoptosis and promotion of cell migration in vascular endothelial cells.	(81, 82)

Supplementary Table 14 *Significantly enriched MSigDB Curated (c2), Gene Ontology (c5) and Hallmarks (H) gene sets within deleted and mutated SCC tumor suppressors.*

**Significantly enriched Curated gene sets (c2)**	**P-value**	**FDR q-value**
TAKADA_GASTRIC_CANCER_COPY_NUMBER_DN	9.64E-10	3.17E-06
DACOSTA_UV_RESPONSE_VIA_ERCC3_DN	1.15E-09	3.17E-06
DER_IFN_BETA_RESPONSE_UP	1.93E-08	3.55E-05
GRESHOCK_CANCER_COPY_NUMBER_UP	5.95E-08	8.22E-05
KUROKAWA_LIVER_CANCER_EARLY_RECURRENCE_DN	8.38E-08	9.27E-05
DER_IFN_ALPHA_RESPONSE_UP	1.49E-07	1.37E-04
BIOCARTA_FAS_PATHWAY	1.74E-07	1.38E-04
LIN_MELANOMA_COPY_NUMBER_DN	6.36E-07	3.80E-04
REACTOME_ENDOSOMAL_VACUOLAR_PATHWAY	6.88E-07	3.80E-04
WUNDER_INFLAMMATORY_RESPONSE_AND_CHOLESTEROL_DN	6.88E-07	3.80E-04
KEGG_PATHWAYS_IN_CANCER	1.07E-06	5.40E-04
BIOCARTA_CTL_PATHWAY	1.19E-06	5.48E-04
BROWNE_HCMV_INFECTION_24HR_DN	4.22E-06	1.79E-03
DER_IFN_GAMMA_RESPONSE_UP	5.88E-06	2.32E-03
BIOCARTA_CTCF_PATHWAY	8.31E-06	3.06E-03
PID_P73PATHWAY	9.01E-06	3.07E-03
REACTOME_ANTIGEN_PRESENTATION_FOLDING_ASSEMBLY_AND_PEPTIDE_LOADING_OF_CLASS_I_MHC	9.43E-06	3.07E-03
DACOSTA_UV_RESPONSE_VIA_ERCC3_COMMON_DN	1.01E-05	3.09E-03
DING_LUNG_CANCER_MUTATED_SIGNIFICANTLY	1.06E-05	3.10E-03
KEGG_SMALL_CELL_LUNG_CANCER	1.15E-05	3.15E-03
Significantly enriched Gene Ontology gene sets (c5)	**P-value**	**FDR q-value**
GO_NEGATIVE_REGULATION_OF_RESPONSE_TO_STIMULUS	1.33E-09	1.36E-05
GO_NEGATIVE_REGULATION_OF_SIGNALING	7.54E-09	3.84E-05
GO_REGULATION_OF_RESPONSE_TO_STRESS	2.44E-08	8.29E-05
GO_GLAND_DEVELOPMENT	4.05E-08	8.61E-05
GO_REGULATION_OF_CELL_DIFFERENTIATION	4.23E-08	8.61E-05
GO_APOPTOTIC_SIGNALING_PATHWAY	5.12E-08	8.70E-05
GO_RESPONSE_TO_ABIOTIC_STIMULUS	8.69E-08	1.19E-04
GO_MESODERM_DEVELOPMENT	9.30E-08	1.19E-04
GO_EPITHELIUM_DEVELOPMENT	1.51E-07	1.58E-04
GO_DIGESTIVE_SYSTEM_DEVELOPMENT	1.55E-07	1.58E-04
GO_REGULATION_OF_PHOSPHORUS_METABOLIC_PROCESS	1.87E-07	1.73E-04
GO_REGULATION_OF_PHOSPHORYLATION	2.97E-07	2.53E-04
GO_REGULATION_OF_APOPTOTIC_SIGNALING_PATHWAY	3.52E-07	2.68E-04
GO_REGULATORY_REGION_NUCLEIC_ACID_BINDING	4.23E-07	2.68E-04
GO_POSITIVE_REGULATION_OF_DEVELOPMENTAL_PROCESS	4.56E-07	2.68E-04
GO_EMBRYONIC_MORPHOGENESIS	5.22E-07	2.68E-04
GO_POSITIVE_REGULATION_OF_APOPTOTIC_SIGNALING_PATHWAY	5.31E-07	2.68E-04
GO_POSITIVE_REGULATION_OF_RNA_METABOLIC_PROCESS	5.49E-07	2.68E-04
GO_CELLULAR_RESPONSE_TO_ENDOGENOUS_STIMULUS	5.58E-07	2.68E-04
GO_IMMUNE_SYSTEM_DEVELOPMENT	5.72E-07	2.68E-04
**Significantly enriched Hallmark gene sets (H)**	**P-value**	**FDR q-value**
HALLMARK_INTERFERON_GAMMA_RESPONSE	2.00E-05	1.00E-03
HALLMARK_INTERFERON_ALPHA_RESPONSE	5.56E-04	1.39E-02
HALLMARK_UV_RESPONSE_DN	1.74E-03	2.90E-02
HALLMARK_APOPTOSIS	2.39E-03	2.99E-02
HALLMARK_ALLOGRAFT_REJECTION	4.39E-03	3.14E-02
HALLMARK_APICAL_JUNCTION	4.39E-03	3.14E-02
HALLMARK_P53_PATHWAY	4.39E-03	3.14E-02

Supplementary Table 15 *Significantly mutated gene sets within deleted and mutated non-SCC tumor suppressors.*

**Significantly enriched Curated gene sets (c2)**	**P-value**	**FDR q-value**
REACTOME_RNA_POLYMERASE_II_TRANSCRIPTION	3.51E-07	1.94E-03
DING_LUNG_CANCER_MUTATED_SIGNIFICANTLY	8.92E-07	2.47E-03
TCGA_GLIOBLASTOMA_COPY_NUMBER_DN	1.54E-06	2.84E-03
LIN_MELANOMA_COPY_NUMBER_DN	3.63E-06	5.02E-03
TCGA_GLIOBLASTOMA_MUTATED	1.43E-05	1.58E-02
HELLEBREKERS_SILENCED_DURING_TUMOR_ANGIOGENESIS	2.45E-05	2.26E-02
DING_LUNG_CANCER_MUTATED_FREQUENTLY	3.37E-05	2.66E-02
FLECHNER_BIOPSY_KIDNEY_TRANSPLANT_REJECTED_VS_OK_DN	4.54E-05	3.14E-02
Significantly enriched Gene Ontology gene sets (c5)	**P-value**	**FDR q-value**
GO_CELL_MORPHOGENESIS_INVOLVED_IN_DIFFERENTIATION	8.57E-07	3.19E-03
GO_NEURON_DEVELOPMENT	9.06E-07	3.19E-03
GO_CELLULAR_COMPONENT_MORPHOGENESIS	1.08E-06	3.19E-03
GO_POSITIVE_REGULATION_OF_CELLULAR_RESPONSE_TO_TRANSFORMING_GROWTH_FACTOR_BETA_STIMULUS	1.25E-06	3.19E-03
GO_CAMERA_TYPE_EYE_MORPHOGENESIS	1.69E-06	3.45E-03
GO_CELL_MORPHOGENESIS_INVOLVED_IN_NEURON_DIFFERENTIATION	3.69E-06	5.28E-03
GO_NEURON_DIFFERENTIATION	4.11E-06	5.28E-03
GO_EYE_MORPHOGENESIS	4.14E-06	5.28E-03
GO_CELLULAR_RESPONSE_TO_ENDOGENOUS_STIMULUS	6.54E-06	7.21E-03
GO_NEGATIVE_REGULATION_OF_CELL_DEATH	7.07E-06	7.21E-03
GO_CELL_PART_MORPHOGENESIS	8.93E-06	8.27E-03
GO_NEUROGENESIS	1.47E-05	1.18E-02
GO_REPRODUCTIVE_SYSTEM_DEVELOPMENT	1.51E-05	1.18E-02
GO_CIRCULATORY_SYSTEM_DEVELOPMENT	1.66E-05	1.21E-02
GO_NEGATIVE_REGULATION_OF_PROTEIN_METABOLIC_PROCESS	1.79E-05	1.22E-02
GO_NEGATIVE_REGULATION_OF_RESPONSE_TO_STIMULUS	2.06E-05	1.31E-02
GO_RESPONSE_TO_ENDOGENOUS_STIMULUS	2.22E-05	1.33E-02
GO_CELL_GROWTH	2.39E-05	1.35E-02
GO_AXON_DEVELOPMENT	3.49E-05	1.83E-02
GO_SENSORY_ORGAN_MORPHOGENESIS	3.59E-05	1.83E-02

Supplementary Table 16 a-c *Focal copy number amplifications across tumors with "TpC-predominant" (n=245) (**a**), "CpG-predominant" (n=87) (**b**) and "Other" (n=39) (**c**) mutations signatures.*

Supplementary Table 16a TpC-predominant tumors

Cytoband	Genomic region	Putative driver gene(s)	Comment	Reference(s)
**1q21.3**	chr1:151300377 - 151331927	*RFX5*	Transcriptional activator in hepatocellular carcinoma.	(2, 3)
**1p31.3**	chr1:61831890 - 61932604	*NFIA*		(1)
**2q33.1**	chr2:201314735 - 201352739	*SPATS2L*		(83)
**3q26.2**	chr3:170766695 - 170820725	*TNIK*	TNIK inhibition abrogates colorectal cancer stemness.	(84)
**5p15.33**	chr5:914233 - 1020354	*TERT*		(85)
**9p24.1**	chr9:5341593 - 5423103	*CD274, PLGRKT, PDCD1LG2*		(42, 86)
**11q22.2**	chr11:102666106 - 102676891	*MMP1*	Prognostic in cervical cancer.	(87)
**11p13**	chr11:35244643 - 35257677	*CD44*		(42, 88)
**11q13.3**	chr11:69873977 - 69989793	*ANO1, CCND1*		(89, 90)
**12p12.1**	chr12:25939146 - 26114034	*KRAS*		(41, 42)
**13q22.1**	chr13:73953432 - 73954914	*LINC00393, KLF5*		(42, 43)
**16p13.13**	chr16:11901466 - 11913383	*BCAR4*		(42)
**17q25.1**	chr17:73270906 - 73296953	*GRB2*		(45)
**18p11.31**	chr18:3155700 - 3254846	*TGIF1*		(6, 7)
**19q13.13**	chr19:38373478 - 38390659	*WDR87*	*Manuscript in preparation.*	

Supplementary Table 16b CpG-predominant tumors

Cytoband	Genomic region	Putative driver gene(s)	Comment	Reference(s)
**3q26.31**	chr3:172093535 - 172160428	*FNDC3B, GHSR*	Induces EMT; upregulated in cervical cancer.	(91, 92)
**4q12**	chr4:56003117 - 56400995	*SRD5A3*	Overexpressed in prostate cancer.	(4)
**17q25.3**	chr17:80648318 - 80648857	*RAB40B*	GTPase that facilitates breast cancer cell invasion.	(93)
**14q12**	chr14:31364669 - 32080009	*NUBPL*	Induces ENT in colorectal cancer.	(94)
**19p13.2**	chr19:11080491 - 11101731	*SMARCA4*		(95)

Supplementary Table 16c 'Other'-subset tumors

Cytoband	Genomic region	Putative driver gene(s)	Comment	Reference(s)
**3q26.33**	chr3:181377182 - 181479964	*SOX2*		(96)
**20q11.21**	chr20:31558283 - 31680334	*BPIFB2*	Has higher expression in gastric carcinomas with high stromal score and is associated with resistance to PD1/PDL1 immunotherapy.	(97)
**21q22.13**	chr21:38450795 - 38682147	*VPS26C*	VPS26C is a member of the Retriever complex for endosomal cargo cycling, required for HPV viral DNA to reach the nucleus, suggesting it can facilitate HPV-driven oncogenesis.	(47)

Supplementary Table 17 *Significantly enriched MSigDB Curated (c2), Gene Ontology (c5) and Hallmarks (H) gene sets within amplified genes found in TpC-, but not CpC-predominant tumors.*

Significantly enriched Curated gene sets (c2)	**P-value**	**FDR q-value**
BIOCARTA_TEL_PATHWAY	8.20E-10	3.59E-06
PROVENZANI_METASTASIS_DN	1.50E-09	3.59E-06
KEGG_GLIOMA	1.95E-09	3.59E-06
KEGG_DORSO_VENTRAL_AXIS_FORMATION	3.65E-09	5.04E-06
NOJIMA_SFRP2_TARGETS_DN	5.13E-09	5.67E-06
KEGG_PATHWAYS_IN_CANCER	6.44E-09	5.93E-06
KEGG_PROSTATE_CANCER	9.66E-09	7.63E-06
KEGG_BLADDER_CANCER	3.80E-08	2.10E-05
REACTOME_EGFR_TRANSACTIVATION_BY_GASTRIN	3.99E-08	2.10E-05
REACTOME_SHC_RELATED_EVENTS_TRIGGERED_BY_IGF1R	3.99E-08	2.10E-05
ZHU_CMV_ALL_DN	4.18E-08	2.10E-05
KEGG_ENDOMETRIAL_CANCER	9.15E-08	4.22E-05
KEGG_NON_SMALL_CELL_LUNG_CANCER	1.07E-07	4.54E-05
PID_SHP2_PATHWAY	1.33E-07	5.26E-05
BARIS_THYROID_CANCER_DN	1.53E-07	5.60E-05
OZANNE_AP1_TARGETS_UP	1.72E-07	5.60E-05
REACTOME_SHC1_EVENTS_IN_EGFR_SIGNALING	1.72E-07	5.60E-05
REACTOME_CONSTITUTIVE_SIGNALING_BY_EGFRVIII	2.15E-07	6.61E-05
REACTOME_GRB2_EVENTS_IN_ERBB2_SIGNALING	2.65E-07	6.97E-05
REACTOME_SIGNALING_BY_ERBB2_ECD_MUTANTS	2.65E-07	6.97E-05
Significantly enriched Gene Ontology gene sets (c5)	**P-value**	**FDR q-value**
GO_POSITIVE_REGULATION_OF_CELL_POPULATION_PROLIFERATION	1.21E-10	1.23E-06
GO_NEGATIVE_REGULATION_OF_TRANSCRIPTION_BY_RNA_POLYMERASE_II	1.76E-08	8.97E-05
GO_RESPONSE_TO_OXYGEN_CONTAINING_COMPOUND	4.56E-08	1.29E-04
GO_REGULATION_OF_CELL_POPULATION_PROLIFERATION	5.08E-08	1.29E-04
GO_POSITIVE_REGULATION_OF_SIGNALING	1.21E-07	2.36E-04
GO_NEGATIVE_REGULATION_OF_NUCLEOBASE_CONTAINING_COMPOUND_METABOLIC_PROCESS	1.39E-07	2.36E-04
GO_POSITIVE_REGULATION_OF_MITOTIC_CELL_CYCLE	2.07E-07	3.02E-04
GO_CELLULAR_RESPONSE_TO_DNA_DAMAGE_STIMULUS	2.95E-07	3.26E-04
GO_CELLULAR_RESPONSE_TO_OXYGEN_CONTAINING_COMPOUND	3.16E-07	3.26E-04
GO_RESPONSE_TO_CYTOKINE	3.20E-07	3.26E-04
GO_REGULATION_OF_CELL_CYCLE	3.53E-07	3.27E-04
GO_CELL_CELL_SIGNALING	4.39E-07	3.73E-04
GO_NEGATIVE_REGULATION_OF_RNA_BIOSYNTHETIC_PROCESS	5.13E-07	4.02E-04
GO_EPITHELIUM_DEVELOPMENT	5.69E-07	4.04E-04
GO_RESPONSE_TO_ENDOGENOUS_STIMULUS	5.95E-07	4.04E-04
GO_POSITIVE_REGULATION_OF_CELL_CYCLE	6.36E-07	4.05E-04
GO_REGULATION_OF_MITOTIC_CELL_CYCLE	8.20E-07	4.49E-04
GO_DOUBLE_STRANDED_DNA_BINDING	8.25E-07	4.49E-04
GO_TRANSCRIPTION_FACTOR_BINDING	8.37E-07	4.49E-04
GO_POSITIVE_REGULATION_OF_INTRACELLULAR_SIGNAL_TRANSDUCTION	1.15E-06	5.88E-04
Significantly enriched Hallmark gene sets (H)	**P-value**	**FDR q-value**
HALLMARK_APOPTOSIS	2.98E-04	4.68E-03
HALLMARK_NOTCH_SIGNALING	3.08E-04	4.68E-03
HALLMARK_IL2_STAT5_SIGNALING	5.54E-04	4.68E-03
HALLMARK_EPITHELIAL_MESENCHYMAL_TRANSITION	5.62E-04	4.68E-03
HALLMARK_ESTROGEN_RESPONSE_EARLY	5.62E-04	4.68E-03
HALLMARK_TNFA_SIGNALING_VIA_NFKB	5.62E-04	4.68E-03
HALLMARK_IL6_JAK_STAT3_SIGNALING	2.26E-03	1.61E-02
HALLMARK_PI3K_AKT_MTOR_SIGNALING	3.27E-03	2.04E-02
HALLMARK_UV_RESPONSE_DN	6.05E-03	3.36E-02
HALLMARK_APICAL_JUNCTION	1.14E-02	4.06E-02
HALLMARK_ESTROGEN_RESPONSE_LATE	1.14E-02	4.06E-02
HALLMARK_G2M_CHECKPOINT	1.14E-02	4.06E-02
HALLMARK_GLYCOLYSIS	1.14E-02	4.06E-02
HALLMARK_KRAS_SIGNALING_UP	1.14E-02	4.06E-02

Supplementary Table 18 *Significantly enriched MSigDB Curated (c2), Gene Ontology (c5) and Hallmarks (H) gene sets within amplified genes found in TpC-predominant, but not in the 'Other'-subset tumors.*

Significantly enriched Curated gene sets (c2)	**P-value**	**FDR q-value**
BIOCARTA_TEL_PATHWAY	9.37E-10	4.25E-06
PROVENZANI_METASTASIS_DN	1.84E-09	4.25E-06
KEGG_GLIOMA	2.31E-09	4.25E-06
KEGG_DORSO_VENTRAL_AXIS_FORMATION	4.17E-09	5.76E-06
NOJIMA_SFRP2_TARGETS_DN	5.86E-09	6.48E-06
KEGG_PATHWAYS_IN_CANCER	8.18E-09	7.54E-06
KEGG_PROSTATE_CANCER	1.14E-08	9.03E-06
KEGG_BLADDER_CANCER	4.34E-08	2.43E-05
REACTOME_EGFR_TRANSACTIVATION_BY_GASTRIN	4.40E-08	2.43E-05
REACTOME_SHC_RELATED_EVENTS_TRIGGERED_BY_IGF1R	4.40E-08	2.43E-05
ZHU_CMV_ALL_DN	4.94E-08	2.48E-05
KEGG_ENDOMETRIAL_CANCER	1.04E-07	4.81E-05
KEGG_NON_SMALL_CELL_LUNG_CANCER	1.22E-07	5.18E-05
PID_SHP2_PATHWAY	1.52E-07	6.00E-05
BARIS_THYROID_CANCER_DN	1.75E-07	6.18E-05
OZANNE_AP1_TARGETS_UP	1.90E-07	6.18E-05
REACTOME_SHC1_EVENTS_IN_EGFR_SIGNALING	1.90E-07	6.18E-05
REACTOME_CONSTITUTIVE_SIGNALING_BY_EGFRVIII	2.37E-07	7.29E-05
REACTOME_GRB2_EVENTS_IN_ERBB2_SIGNALING	2.92E-07	7.69E-05
REACTOME_SIGNALING_BY_ERBB2_ECD_MUTANTS	2.92E-07	7.69E-05
Significantly enriched Gene Ontology gene sets (c5)	**P-value**	**FDR q-value**
GO_POSITIVE_REGULATION_OF_CELL_POPULATION_PROLIFERATION	7.93E-12	8.08E-08
GO_NEGATIVE_REGULATION_OF_TRANSCRIPTION_BY_RNA_POLYMERASE_II	1.24E-09	6.34E-06
GO_REGULATION_OF_CELL_POPULATION_PROLIFERATION	5.98E-09	2.03E-05
GO_NEGATIVE_REGULATION_OF_NUCLEOBASE_CONTAINING_COMPOUND_METABOLIC_PROCESS	1.53E-08	3.16E-05
GO_POSITIVE_REGULATION_OF_SIGNALING	1.55E-08	3.16E-05
GO_CELLULAR_RESPONSE_TO_DNA_DAMAGE_STIMULUS	2.31E-08	3.92E-05
GO_NEGATIVE_REGULATION_OF_RNA_BIOSYNTHETIC_PROCESS	5.40E-08	6.00E-05
GO_CELL_CELL_SIGNALING	5.47E-08	6.00E-05
GO_EPITHELIUM_DEVELOPMENT	6.06E-08	6.00E-05
GO_REPRODUCTIVE_SYSTEM_DEVELOPMENT	6.39E-08	6.00E-05
GO_RESPONSE_TO_OXYGEN_CONTAINING_COMPOUND	6.68E-08	6.00E-05
GO_DOUBLE_STRANDED_DNA_BINDING	7.38E-08	6.00E-05
GO_RESPONSE_TO_ENDOGENOUS_STIMULUS	7.65E-08	6.00E-05
GO_REPRODUCTION	1.80E-07	1.31E-04
GO_POSITIVE_REGULATION_OF_MITOTIC_CELL_CYCLE	2.45E-07	1.66E-04
GO_MORPHOGENESIS_OF_AN_EPITHELIUM	2.73E-07	1.74E-04
GO_SEQUENCE_SPECIFIC_DNA_BINDING	3.74E-07	2.24E-04
GO_CELLULAR_RESPONSE_TO_OXYGEN_CONTAINING_COMPOUND	4.27E-07	2.32E-04
GO_RESPONSE_TO_CYTOKINE	4.33E-07	2.32E-04
GO_REGULATION_OF_CELL_CYCLE	4.77E-07	2.43E-04
Significantly enriched Hallmark gene sets (H)	**P-value**	**FDR q-value**
HALLMARK_APOPTOSIS	3.28E-04	5.14E-03
HALLMARK_NOTCH_SIGNALING	3.28E-04	5.14E-03
HALLMARK_IL2_STAT5_SIGNALING	6.08E-04	5.14E-03
HALLMARK_EPITHELIAL_MESENCHYMAL_TRANSITION	6.17E-04	5.14E-03
HALLMARK_ESTROGEN_RESPONSE_EARLY	6.17E-04	5.14E-03
HALLMARK_TNFA_SIGNALING_VIA_NFKB	6.17E-04	5.14E-03
HALLMARK_IL6_JAK_STAT3_SIGNALING	2.41E-03	1.72E-02
HALLMARK_PI3K_AKT_MTOR_SIGNALING	3.48E-03	2.18E-02
HALLMARK_UV_RESPONSE_DN	6.43E-03	3.57E-02
HALLMARK_APICAL_JUNCTION	1.21E-02	4.31E-02
HALLMARK_ESTROGEN_RESPONSE_LATE	1.21E-02	4.31E-02
HALLMARK_G2M_CHECKPOINT	1.21E-02	4.31E-02
HALLMARK_GLYCOLYSIS	1.21E-02	4.31E-02
HALLMARK_KRAS_SIGNALING_UP	1.21E-02	4.31E-02

Supplementary Table 19 a-*c* *Focal copy number deletions across tumors with "TpC-predominant" (n=245) (**a**), "CpG-predominant" (n=87) (**b**) and "Other" (n=39) (**c**) mutations signatures.*

Supplementary Table 19a Tp*C-predominant tumors				
Cytoband	***Genomic region***	***Putative driver gene(s)***	***Comment***	***Reference(s)***
**1p13.2**	chr1:112912722 - 113195407	*ST7L*		(53)
**1p36.22**	chr1:12225351 - 12269707	*MIR4632, TNFRSF1B*	Possibly paradoxical; TNFRSF1B has oncogenic properties in several cancer types	(98)
**2q37.1**	chr2:234825559 - 234929559	*SH3BP4*	Negative regulator of mTORC1 signaling and inhibits beta-catenin nuclear localization	(99, 100)
**2q37.3**	chr2:239748320 - 240337482	*TWIST2*	Tumor suppressor in murine osteosarcoma. Activates p21 in AML.	(101, 102)
**4q21.3**	chr4:87511571 - 87737413	*PTPN13*	Loss is associated with HPV-induced oncogenic cervical transformation. Expression can by restored by curcumin treatment of cervical cancer cells.	(57, 58)
**4q35.2**	chr4:187508513 - 187652260	*FAT1*		(42)
**5q31.1**	chr5:131817027 - 131827191	*IRF1*		(103)
**5q35.2**	chr5:175082112 - 175113539	*HRH2*		(104)
**6p25.3**	chr6:1309918 - 1315309	*FOXQ1*	Possibly paradoxical; FOXQ1 has oncogenic properties in several cancer types	(18)
**6q25.3**	chr6:157775999 - 158099269	*ZDHHC14*		(105)
**7q31.1**	chr7:110239721 - 111353361	*LRRN3*		
**7q36.1**	chr7:151830319 - 152135594	*KMT2C*		(106)
**11p15.1**	chr11:19371907 - 20143721	*E2F8*		(107, 108)
**11q23.3**	chr11:119492338 - 119604854	*PVRL1*	PVRL1 is a metastasis suppressor gene in melanoma.	(23)
**11q25**	chr11:133708065 - 133768886	*MIR4697*		
**13q13.3**	chr13:37389687 - 37404660	*RFXAP*		(109)
**14q32.31**	chr14:102972764 - 103011886	*ANKRD9*		(110)
**15q15.1**	chr15:42138456 - 42186351	*MIR4310*	Negative transcriptional regulator of MMP-10 which promotes cervical cancer progression.	(26, 27)
**16q11.2**	chr16:31850299 - 46615362	*ZNF267, TP53TG3, TP53TG3D, TP53TG3C, TP53TG3B*	Induced by TP53.	(28)
**16q24.1**	chr16:86506106 - 86555873	*FENDRR, FOXF1*	lncRNA FENDRR inhibits progression of lung cancer, colon cancer, and cholangiocarcinoma.	(111-115)
**17p12**	chr17:11921944 - 12047931	*MIR744, MAP2K4*	Possibly paradoxical; MAP2K4 in an oncogene in breast cancer.	(116)
**17q25.3**	chr17:80917016 - 80917016	*METRNL FOXK2, ZNF750*	METRNL regulates immune-adipose interactions and insulin resistance. FOXK2 is a transcriptional repressor, which inhibits hypoxic response and suppresses breast cancer proliferation and invasion.	(69, 71)
**18q23**	chr18:74240503 - 74326105	*LINC00908*		(72)
**19q13.41**	chr19:52766328 - 52795985	*MIR643*		(117)
**20p12.1**	chr20:13974549 - 16036115	*MACROD2, FLRT3*		(118)
**21q11.2**	chr21:15347621 - 15372509	*ANKRD20A11P*		
**22q13.31**	chr22:46505806 - 46511645	*MIRLET7A3, MIRLET7B*		(119, 120)

**Xp11.3**	chrX:44729855 - 44975013	*KDM6A*	Inactivated in multiple cancers.	(29-31)
**Xp21.1**	chrX:30873422 - 34033463	*DMD*		(121)
**Xq21.33**	chrX:95939469 - 96860054	*RPA4*	Replication factor essential for DNA repair; RPA4 expression is decreased in cancerous tissues.	(74, 75)

Supplementary Table 19b *CpG-predominant tumors

Cytoband	*Genomic region*	*Putative driver gene(s)*	*Comment*	*Reference(s)*
**1p36.22**	chr1:10531619 - 10694040	*PEX14*		(52)
**2q37.3**	chr2:23839114 3 - 238466356	*RAB17*		(122)
**3p14.1**	chr3:69152593 - 69186319	*ARL6IP5*		(123)
**4q22.1**	chr4:91039783 - 92523213	*CCSER1*	Patel et al demonstrated that CCSER1 (aka FAM190A) deficient is associated with a cell division defect.	(18)
**4q35.2**	chr4:18876224 0 - 188762240	*TRIML2*		(124)
**5q12.1**	chr5:58261529-59818040	*PDE4D*		
**5q12.1**	chr5:58261529-59818040	*PDE4D*	Although thought to have pro-cancer properties, PDE4D deletions have been observed in multiple tumor types.	(14, 15)
**7q31.33**	chr7:12605852 2 - 126898623	*GRM8*		(125)
**7p22.3**	chr7:762577 - 832985	*PRKAR1B*		(126)
**11q24.1**	chr11:1233957 13 - 123502821	*GRAMD1B*		(127)
**11q25**	chr11:1312398 04 - 132208428	*NTM, OPCML*	NTM belongs to the IgLON family of GPI-anchored cell adhesion molecules, which is part of the immunoglobulin (Ig) domain-containing superfamily.	(24)
**11q14.2**	chr11:8580323 8 - 85805593	*PICALM, EED*	EED is a context-dependent tumor-suppressor in KRAS-driven lung cancer.	(21, 22)
**13q14.2**	chr13:5010505 5 - 50160945	*RCBTB1*	Deletion is associated with sarcoma metastasis and docetaxel resistance.	(66)
**18p11.32**	chr18:2846246 - 2916005	*EMILIN2*		(128)
18q21.2	*chr18:4849216 8 - 48616656*	*SMAD4*	*Smad4/DPC4-mediated tumor suppression through suppression of angiogenesis.*	*(80)*
**19q13.11**	chr19:3428334 0 - 34308164	*KCTD15*		(129)

Supplementary Table 19c 'Other'-subset tumors

**Cytoband**	**Genomic region**	**Putative driver gene(s)**	**Comment**	**Reference(s)**
**1p36.32**	chr1:3218610 - 3819336	*TP73, PRDM16*	PRDM16 suppresses lung adenocarcinoma metastasis.	(76)
**2q37.1**	chr2:233034495 - 233048132	*MIR562*	A putative role of miR-562 as the tumor suppressor in the 2q37.1 deletion peak in Wilms' tumors.	(10)
**4q35.2**	chr4:187383890 - 187479257	*MTNR1A*	Paradoxical; oncogene in gastric adenocarcinoma.	(130)
**5q12.1**	chr5:60621045 - 60842284	*ZSWIM6*		
**7p22.3**	chr7:762577 - 832985	*PRKAR1B*		(126)
**8p23.3**	chr8:42933372 - 47460945	*CSMD1*		(19)
**11q14.1**	chr11:78363072 - 79153837	*TENM4*		(131)
**13q12.11**	chr13:19520887 - 19643178	*LINC00442*		
**15q21.1**	chr15:47474882 - 48066582	*COPS2, SEMA6D*		(132)
**17p13.3**	chr17:3118733 - 3119871	*RAP1GAP2*		(133)

Supplementary Table 20 *DNA repair pathway genes*

**Supplementary Table 20a.** *276 curated DNA repair pathway genes from Knijnenburg et al 2018* **.**			
AEN	*FANCF*	*PARPBP*	*RNMT*
ALKBH1	*FANCG*	*PAXIP1*	*RPA1*
ALKBH2	*FANCI*	*PCNA*	*RPA2*
ALKBH3	*FANCL*	*PER1*	*RPA3*
APEX1	*FANCM*	*PLK3*	*RPA4*
APEX2	*FEN1*	*PLRG1*	*RRM1*
APITD1	*GADD45A*	*PMS1*	*RRM2*
APLF	*GADD45G*	*PMS2*	*RRM2B*
APTX	*GEN1*	*PNKP*	*RTEL1*
ASCC3	*GTF2H1*	*POLA1*	*SETMAR*
ATM	*GTF2H2*	*POLB*	*SHFM1*
ATR	*GTF2H3*	*POLD1*	*SHPRH*
ATRIP	*GTF2H4*	*POLD2*	*SLX1A*
ATRX	*GTF2H5*	*POLD3*	*SLX1B*
BABAM1	*H2AFX*	*POLD4*	*SLX4*
BARD1	*HELQ*	*POLE*	*SMARCA4*
BCAS2	*HERC2*	*POLE2*	*SMARCAD1*
BLM	*HES1*	*POLE3*	*SMARCC1*
BRCA1	*HFM1*	*POLE4*	*SMC5*
BRCA2	*HLTF*	*POLG*	*SMC6*
BRCC3	*HMGB1*	*POLH*	*SMUG1*
BRE	*HMGB2*	*POLI*	*SOX4*
BRIP1	*HUS1*	*POLK*	*SPO11*
CCNH	*IDH1*	*POLL*	*SPRTN*
CDC25A	*INO80*	*POLM*	*STRA13*
CDC25B	*KAT5*	*POLN*	*SWI5*
CDC25C	*LIG1*	*POLQ*	*SWSAP1*
CDC5L	*LIG3*	*PPP4C*	*TCEA1*
CDK7	*LIG4*	*PPP4R1*	*TCEB1*
CETN2	*MAD2L2*	*PPP4R2*	*TCEB2*
CHAF1A	*MBD4*	*PPP4R4*	*TCEB3*
CHEK1	*MDC1*	*PRKDC*	*TDG*
CHEK2	*MGMT*	*PRPF19*	*TDP1*
CLK2	*MLH1*	*PTEN*	*TDP2*
CUL3	*MLH3*	*RAD1*	*TELO2*
CUL4A	*MMS19*	*RAD17*	*TOP3A*
CUL5	*MNAT1*	*RAD18*	*TOP3B*
DCLRE1A	*MORF4L1*	*RAD23A*	*TOPBP1*
DCLRE1B	*MPG*	*RAD23B*	*TP53*
DCLRE1C	*MPLKIP*	*RAD50*	*TP53BP1*
DDB1	*MRE11A*	*RAD51*	*TREX1*
DDB2	*MRPL40*	*RAD51B*	*TREX2*
DMC1	*MSH2*	*RAD51C*	*TTK*
DNA2	*MSH3*	*RAD51D*	*TYMS*
DNTT	*MSH6*	*RAD52*	*UBE2A*
DUT	*MUS81*	*RAD54B*	*UBE2B*
EID3	*MUTYH*	*RAD54L*	*UBE2N*
EME1	*NABP2*	*RAD9A*	*UBE2T*
EME2	*NBN*	*RAD9B*	*UBE2V2*
ENDOV	*NEIL1*	*RBBP8*	*UIMC1*
ERCC1	*NEIL2*	*RBX1*	*UNG*
ERCC2	*NEIL3*	*RDM1*	*USP1*
ERCC3	*NFATC2IP*	*RECQL*	*UVSSA*
ERCC4	*NHEJ1*	*RECQL4*	*WDR48*
ERCC5	*NSMCE1*	*RECQL5*	*WEE1*
ERCC6	*NSMCE2*	*REV1*	*WRN*
ERCC8	*NSMCE3*	*REV3L*	*XAB2*
EXO1	*NSMCE4A*	*RFC1*	*XPA*
EXO5	*NTHL1*	*RFC2*	*XPC*
FAAP100	*NUDT1*	*RFC3*	*XRCC1*
FAAP20	*NUDT15*	*RFC4*	*XRCC2*
FAAP24	*NUDT18*	*RFC5*	*XRCC3*
FAM175A	*OGG1*	*RIF1*	*XRCC4*
FAN1	*PALB2*	*RMI1*	*XRCC5*
FANCA	*PARG*	*RMI2*	*XRCC6*
FANCB	*PARP1*	*RNF168*	*YWHAB*
FANCC	*PARP2*	*RNF169*	*YWHAE*
FANCD2	*PARP3*	*RNF4*	*YWHAG*
FANCE	*PARP4*	*RNF8*	*ZSWIM7*

Supplementary Table 20b. *255 DNA repair pathway overlapping with the Mutational allelic frequency (MAF) file* **.**

AEN	*GADD45G*	*POLD1*	*SMARCC1*
ALKBH1	*GEN1*	*POLD2*	*SMC5*
ALKBH2	*GTF2H1*	*POLD3*	*SMC6*
ALKBH3	*GTF2H2*	*POLD4*	*SMUG1*
APEX1	*GTF2H3*	*POLE*	*SOX4*
APEX2	*GTF2H4*	*POLE2*	*SPO11*
APITD1	*GTF2H5*	*POLE3*	*STRA13*
APLF	*H2AFX*	*POLE4*	*TCEA1*
APTX	*HELQ*	*POLG*	*TCEB1*
ASCC3	*HERC2*	*POLH*	*TCEB2*
ATM	*HES1*	*POLI*	*TCEB3*
ATR	*HFM1*	*POLK*	*TDG*
ATRIP	*HLTF*	*POLL*	*TDP1*
ATRX	*HMGB1*	*POLM*	*TDP2*
BARD1	*HMGB2*	*POLN*	*TELO2*
BCAS2	*HUS1*	*POLQ*	*TOP3A*
BLM	*IDH1*	*PPP4C*	*TOP3B*
BRCA1	*INO80*	*PPP4R1*	*TOPBP1*
BRCA2	*KAT5*	*PPP4R2*	*TP53*
BRCC3	*LIG1*	*PPP4R4*	*TP53BP1*
BRE	*LIG3*	*PRKDC*	*TREX1*
BRIP1	*LIG4*	*PRPF19*	*TREX2*
CCNH	*MAD2L2*	*PTEN*	*TTK*
CDC25A	*MBD4*	*RAD1*	*TYMS*
CDC25B	*MDC1*	*RAD17*	*UBE2A*
CDC25C	*MGMT*	*RAD18*	*UBE2B*
CDC5L	*MLH1*	*RAD23A*	*UBE2N*
CDK7	*MLH3*	*RAD23B*	*UBE2T*
CETN2	*MMS19*	*RAD50*	*UBE2V2*
CHAF1A	*MNAT1*	*RAD51*	*UIMC1*
CHEK1	*MORF4L1*	*RAD51C*	*UNG*
CHEK2	*MPG*	*RAD52*	*USP1*
CLK2	*MRE11A*	*RAD54B*	*WDR48*
CUL3	*MRPL40*	*RAD54L*	*WEE1*
CUL4A	*MSH2*	*RAD9A*	*WRN*
CUL5	*MSH3*	*RAD9B*	*XAB2*
DCLRE1A	*MSH6*	*RBBP8*	*XPA*
DCLRE1B	*MUS81*	*RBX1*	*XPC*
DCLRE1C	*MUTYH*	*RDM1*	*XRCC1*
DDB1	*NBN*	*RECQL*	*XRCC2*
DDB2	*NEIL1*	*RECQL4*	*XRCC3*
DMC1	*NEIL2*	*RECQL5*	*XRCC4*
DNA2	*NEIL3*	*REV1*	*XRCC5*
DNTT	*NFATC2IP*	*REV3L*	*XRCC6*
DUT	*NHEJ1*	*RFC1*	*YWHAB*
EID3	*NSMCE1*	*RFC2*	*YWHAE*
EME1	*NSMCE2*	*RFC3*	*YWHAG*
EME2	*NSMCE4A*	*RFC4*	*ZSWIM7*
ERCC1	*NTHL1*	*RFC5*	
ERCC2	*NUDT1*	*RIF1*	
ERCC3	*NUDT15*	*RMI1*	
ERCC4	*NUDT18*	*RNF168*	
ERCC5	*OGG1*	*RNF169*	
ERCC6	*PALB2*	*RNF4*	
ERCC8	*PARG*	*RNF8*	
EXO1	*PARP1*	*RNMT*	
FAM175A	*PARP2*	*RPA1*	
FANCA	*PARP3*	*RPA2*	
FANCB	*PARP4*	*RPA3*	
FANCC	*PAXIP1*	*RPA4*	
FANCD2	*PCNA*	*RRM1*	
FANCE	*PER1*	*RRM2*	
FANCF	*PLK3*	*RRM2B*	
FANCG	*PLRG1*	*RTEL1*	
FANCI	*PMS1*	*SETMAR*	
FANCL	*PMS2*	*SHFM1*	
FANCM	*PNKP*	*SHPRH*	
FEN1	*POLA1*	*SMARCA4*	
GADD45A	*POLB*	*SMARCAD1*	

REFERENCES

1. Hoxha E, Andreas K, Song H-R. BIOL-06. NOVEL ONCOGENIC ROLE FOR NFIA IN GLIOMA. Neuro-Oncology. 2017;19(suppl_4):iv4-iv.
2. Zhao Y, Xie X, Liao W, Zhang H, Cao H, Fei R, et al. The transcription factor RFX5 is a transcriptional activator of the TPP1 gene in hepatocellular carcinoma. Oncol Rep. 2017;37(1):289-96.
3. Chen DB, Zhao YJ, Wang XY, Liao WJ, Chen P, Deng KJ, et al. Regulatory factor X5 promotes hepatocellular carcinoma progression by transactivating tyrosine 3-monooxygenase/tryptophan 5-monooxygenase activation protein theta and suppressing apoptosis. Chin Med J (Engl). 2019;132(13):1572-81.
4. Uemura M, Tamura K, Chung S, Honma S, Okuyama A, Nakamura Y, et al. Novel 5 alpha-steroid reductase (SRD5A3, type-3) is overexpressed in hormone-refractory prostate cancer. Cancer Sci. 2008;99(1):81-6.
5. Krishnan R, Boddapati N, Mahalingam S. Interplay between human nucleolar GNL1 and RPS20 is critical to modulate cell proliferation. Sci Rep. 2018;8(1):11421.
6. Xiang G, Yi Y, Weiwei H, Weiming W. TGIF1 promoted the growth and migration of cancer cells in nonsmall cell lung cancer. Tumour Biol. 2015;36(12):9303-10.
7. Wang Y, Pan T, Wang H, Li L, Li J, Zhang C, et al. Silencing of TGIF attenuates the tumorigenicity of A549 cells in vitro and in vivo. Tumour Biol. 2016;37(9):12725-30.
8. Baldwin A, Pirisi L, Creek KE. NFI-Ski interactions mediate transforming growth factor beta modulation of human papillomavirus type 16 early gene expression. J Virol. 2004;78(8):3953-64.
9. Glinsky GV, Berezovska O, Glinskii AB. Microarray analysis identifies a death-from-cancer signature predicting therapy failure in patients with multiple types of cancer. J Clin Invest. 2005;115(6):1503-21.
10. Drake KM, Ruteshouser EC, Natrajan R, Harbor P, Wegert J, Gessler M, et al. Loss of heterozygosity at 2q37 in sporadic Wilms' tumor: putative role for miR-562. Clin Cancer Res. 2009;15(19):5985-92.
11. Liu W, Yu Q, Ma J, Cheng Y, Zhang H, Luo W, et al. Knockdown of a DIS3L2 promoter upstream long noncoding RNA (AC105461.1) enhances colorectal cancer stem cell properties in vitro by down-regulating DIS3L2. Onco Targets Ther. 2017;10:2367-76.
12. Astuti D, Morris MR, Cooper WN, Staals RH, Wake NC, Fews GA, et al. Germline mutations in DIS3L2 cause the Perlman syndrome of overgrowth and Wilms tumor susceptibility. Nat Genet. 2012;44(3):277-84.
13. Patel K, Scrimieri F, Ghosh S, Zhong J, Kim MS, Ren YR, et al. FAM190A deficiency creates a cell division defect. Am J Pathol. 2013;183(1):296-303.
14. Lin DC, Xu L, Ding LW, Sharma A, Liu LZ, Yang H, et al. Genomic and functional characterizations of phosphodiesterase subtype 4D in human cancers. Proc Natl Acad Sci U S A. 2013;110(15):6109-14.
15. Cheng J, Demeulemeester J, Wedge DC, Vollan HKM, Pitt JJ, Russnes HG, et al. Pan-cancer analysis of homozygous deletions in primary tumours uncovers rare tumour suppressors. Nat Commun. 2017;8(1):1221.
16. Bagati A, Bianchi-Smiraglia A, Moparthy S, Kolesnikova K, Fink EE, Lipchick BC, et al. Melanoma Suppressor Functions of the Carcinoma Oncogene FOXQ1. Cell Rep. 2017;20(12):2820-32.
17. Agirre X, Roman-Gomez J, Vazquez I, Jimenez-Velasco A, Garate L, Montiel-Duarte C, et al. Abnormal methylation of the common PARK2 and PACRG promoter is associated with downregulation of gene expression in acute lymphoblastic leukemia and chronic myeloid leukemia. Int J Cancer. 2006;118(8):1945-53.
18. Poulogiannis G, McIntyre RE, Dimitriadi M, Apps JR, Wilson CH, Ichimura K, et al. PARK2 deletions occur frequently in sporadic colorectal cancer and accelerate adenoma development in Apc mutant mice. Proc Natl Acad Sci U S A. 2010;107(34):15145-50.
19. Escudero-Esparza A, Bartoschek M, Gialeli C, Okroj M, Owen S, Jirström K, et al. Complement inhibitor CSMD1 acts as tumor suppressor in human breast cancer. Oncotarget. 2016;7(47):76920-33.
20. Gao G, Johnson SH, Vasmatzis G, Pauley CE, Tombers NM, Kasperbauer JL, et al. Common fragile sites (CFS) and extremely large CFS genes are targets for human papillomavirus integrations and chromosome rearrangements in oropharyngeal squamous cell carcinoma. Genes Chromosomes Cancer. 2017;56(1):59-74.

21. Serresi M, Gargiulo G, Proost N, Siteur B, Cesaroni M, Koppens M, et al. Polycomb Repressive Complex 2 Is a Barrier to KRAS-Driven Inflammation and Epithelial-Mesenchymal Transition in Non-Small-Cell Lung Cancer. Cancer Cell. 2016;29(1):17-31.
22. Mercer JL, Argus JP, Crabtree DM, Keenan MM, Wilks MQ, Chi JT, et al. Modulation of PICALM Levels Perturbs Cellular Cholesterol Homeostasis. PLoS One. 2015;10(6):e0129776.
23. Ablain J, Dang M, Rothschild H, Zon LI. Abstract 93: Tissue-specific CRISPR in zebrafish identifies PVRL1 as a novel metastasis suppressor gene in melanoma. Cancer Research. 2018;78(13 Supplement):93-.
24. Sellar GC, Watt KP, Rabiasz GJ, Stronach EA, Li L, Miller EP, et al. OPCML at 11q25 is epigenetically inactivated and has tumor-suppressor function in epithelial ovarian cancer. Nat Genet. 2003;34(3):337-43.
25. Han M, Wang S, Yang N, Wang X, Zhao W, Saed HS, et al. Therapeutic implications of altered cholesterol homeostasis mediated by loss of CYP46A1 in human glioblastoma. EMBO Mol Med. 2020;12(1):e10924.
26. Schnabl B, Hu K, Mühlbauer M, Hellerbrand C, Stefanovic B, Brenner DA, et al. Zinc finger protein 267 is up-regulated during the activation process of human hepatic stellate cells and functions as a negative transcriptional regulator of MMP-10. Biochem Biophys Res Commun. 2005;335(1):87-96.
27. Zhang G, Miyake M, Lawton A, Goodison S, Rosser CJ. Matrix metalloproteinase-10 promotes tumor progression through regulation of angiogenic and apoptotic pathways in cervical tumors. BMC Cancer. 2014;14:310.
28. Ng CC, Koyama K, Okamura S, Kondoh H, Takei Y, Nakamura Y. Isolation and characterization of a novel TP53-inducible gene, TP53TG3. Genes Chromosomes Cancer. 1999;26(4):329-35.
29. Ler LD, Ghosh S, Chai X, Thike AA, Heng HL, Siew EY, et al. Loss of tumor suppressor KDM6A amplifies PRC2-regulated transcriptional repression in bladder cancer and can be targeted through inhibition of EZH2. Sci Transl Med. 2017;9(378).
30. Ezponda T, Dupéré-Richer D, Will CM, Small EC, Varghese N, Patel T, et al. UTX/KDM6A Loss Enhances the Malignant Phenotype of Multiple Myeloma and Sensitizes Cells to EZH2 inhibition. Cell Rep. 2017;21(3):628-40.
31. Schulz WA, Lang A, Koch J, Greife A. The histone demethylase UTX/KDM6A in cancer: Progress and puzzles. Int J Cancer. 2019;145(3):614-20.
32. Turner KM, Sun Y, Ji P, Granberg KJ, Bernard B, Hu L, et al. Genomically amplified Akt3 activates DNA repair pathway and promotes glioma progression. Proc Natl Acad Sci U S A. 2015;112(11):3421-6.
33. Yi M, Tan Y, Wang L, Cai J, Li X, Zeng Z, et al. TP63 links chromatin remodeling and enhancer reprogramming to epidermal differentiation and squamous cell carcinoma development. Cell Mol Life Sci. 2020.
34. Wee P, Wang Z. Epidermal Growth Factor Receptor Cell Proliferation Signaling Pathways. Cancers (Basel). 2017;9(5).
35. Iida K, Nakayama K, Rahman MT, Rahman M, Ishikawa M, Katagiri A, et al. EGFR gene amplification is related to adverse clinical outcomes in cervical squamous cell carcinoma, making the EGFR pathway a novel therapeutic target. Br J Cancer. 2011;105(3):420-7.
36. Iwai Y, Ishida M, Tanaka Y, Okazaki T, Honjo T, Minato N. Involvement of PD-L1 on tumor cells in the escape from host immune system and tumor immunotherapy by PD-L1 blockade. Proc Natl Acad Sci U S A. 2002;99(19):12293-7.
37. Roemer MG, Advani RH, Ligon AH, Natkunam Y, Redd RA, Homer H, et al. PD-L1 and PD-L2 Genetic Alterations Define Classical Hodgkin Lymphoma and Predict Outcome. J Clin Oncol. 2016;34(23):2690-7.
38. Zhu G, Cheng Z, Huang Y, Zheng W, Yang S, Lin C, et al. TRAF6 promotes the progression and growth of colorectal cancer through nuclear shuttle regulation NF-kB/c-jun signaling pathway. Life Sci. 2019;235:116831.
39. Sugahara K, Michikawa Y, Ishikawa K, Shoji Y, Iwakawa M, Shibahara T, et al. Combination effects of distinct cores in 11q13 amplification region on cervical lymph node metastasis of oral squamous cell carcinoma. Int J Oncol. 2011;39(4):761-9.

40. Cheng L, Zhou Z, Flesken-Nikitin A, Toshkov IA, Wang W, Camps J, et al. Rb inactivation accelerates neoplastic growth and substitutes for recurrent amplification of cIAP1, cIAP2 and Yap1 in sporadic mammary carcinoma associated with p53 deficiency. Oncogene. 2010;29(42):5700-11.
41. Pylayeva-Gupta Y, Grabocka E, Bar-Sagi D. RAS oncogenes: weaving a tumorigenic web. Nat Rev Cancer. 2011;11(11):761-74.
42. Integrated genomic and molecular characterization of cervical cancer. Nature. 2017;543(7645):378-84.
43. Zhang L, Wu Y, Wu J, Zhou M, Li D, Wan X, et al. KLF5-mediated COX2 upregulation contributes to tumorigenesis driven by PTEN deficiency. Cell Signal. 2020:109767.
44. Warburton AJ, Boone DN. Insights from Global Analyses of Long Noncoding RNAs in Breast Cancer. Curr Pathobiol Rep. 2017;5(1):23-34.
45. Beigbeder A, Chartier FJM, Bisson N. MPZL1 forms a signalling complex with GRB2 adaptor and PTPN11 phosphatase in HER2-positive breast cancer cells. Sci Rep. 2017;7(1):11514.
46. Turnham DJ, Yang WW, Davies J, Varnava A, Ridley AJ, Conlan RS, et al. Bcl-3 promotes multi-modal tumour cell migration via NF-κB1 mediated regulation of Cdc42. Carcinogenesis. 2020.
47. McNally KE, Faulkner R, Steinberg F, Gallon M, Ghai R, Pim D, et al. Retriever is a multiprotein complex for retromer-independent endosomal cargo recycling. Nat Cell Biol. 2017;19(10):1214-25.
48. Dorfleutner A, Bryan NB, Talbott SJ, Funya KN, Rellick SL, Reed JC, et al. Cellular pyrin domain-only protein 2 is a candidate regulator of inflammasome activation. Infect Immun. 2007;75(3):1484-92.
49. Sengelaub CA, Navrazhina K, Ross JB, Halberg N, Tavazoie SF. PTPRN2 and PLCβ1 promote metastatic breast cancer cell migration through PI(4,5)P2-dependent actin remodeling. Embo j. 2016;35(1):62-76.
50. Natrajan R, Mackay A, Wilkerson PM, Lambros MB, Wetterskog D, Arnedos M, et al. Functional characterization of the 19q12 amplicon in grade III breast cancers. Breast Cancer Res. 2012;14(2):R53.
51. Shastrula PK, Lund PJ, Garcia BA, Janicki SM. Rpp29 regulates histone H3.3 chromatin assembly through transcriptional mechanisms. J Biol Chem. 2018;293(32):12360-77.
52. Okumoto K, Shermely ME, Natsui M, Kosako H, Natsuyama R, Marutani T, et al. The peroxisome counteracts oxidative stresses by suppressing catalase import via Pex14 phosphorylation. Elife. 2020;9.
53. Katoh M. Molecular cloning and characterization of ST7R (ST7-like, ST7L) on human chromosome 1p13, a novel gene homologous to tumor suppressor gene ST7 on human chromosome 7q31. Int J Oncol. 2002;20(6):1247-53.
54. Lucas B, Grigo K, Erdmann S, Lausen J, Klein-Hitpass L, Ryffel GU. HNF4alpha reduces proliferation of kidney cells and affects genes deregulated in renal cell carcinoma. Oncogene. 2005;24(42):6418-31.
55. Morita M, Ler LW, Fabian MR, Siddiqui N, Mullin M, Henderson VC, et al. A novel 4EHP-GIGYF2 translational repressor complex is essential for mammalian development. Mol Cell Biol. 2012;32(17):3585-93.
56. Lee TG, Jeong EH, Kim SY, Kim HR, Kim H, Kim CH. Fhit, a tumor suppressor protein, induces autophagy via 14-3-3τ in non-small cell lung cancer cells. Oncotarget. 2017;8(19):31923-37.
57. Hoover AC, Strand GL, Nowicki PN, Anderson ME, Vermeer PD, Klingelhutz AJ, et al. Impaired PTPN13 phosphatase activity in spontaneous or HPV-induced squamous cell carcinomas potentiates oncogene signaling through the MAP kinase pathway. Oncogene. 2009;28(45):3960-70.
58. Maher DM, Bell MC, O'Donnell EA, Gupta BK, Jaggi M, Chauhan SC. Curcumin suppresses human papillomavirus oncoproteins, restores p53, Rb, and PTPN13 proteins and inhibits benzo[a]pyrene-induced upregulation of HPV E7. Mol Carcinog. 2011;50(1):47-57.
59. Ding W, Fan XL, Xu X, Huang JZ, Xu SH, Geng Q, et al. Epigenetic silencing of ITGA2 by MiR-373 promotes cell migration in breast cancer. PLoS One. 2015;10(8):e0135128.
60. Kang YP, Yoon JH, Long NP, Koo GB, Noh HJ, Oh SJ, et al. Spheroid-Induced Epithelial-Mesenchymal Transition Provokes Global Alterations of Breast Cancer Lipidome: A Multi-Layered Omics Analysis. Front Oncol. 2019;9:145.
61. Ding Y, Yang J, Ma Y, Yao T, Chen X, Ge S, et al. MYCN and PRC1 cooperatively repress docosahexaenoic acid synthesis in neuroblastoma via ELOVL2. J Exp Clin Cancer Res. 2019;38(1):498.

62. Wang S, Dong Y, Zhang Y, Wang X, Xu L, Yang S, et al. DACT2 is a functional tumor suppressor through inhibiting Wnt/β-catenin pathway and associated with poor survival in colon cancer. Oncogene. 2015;34(20):2575-85.
63. Chang HY, Fan CC, Chu PC, Hong BE, Lee HJ, Chang MS. hPuf-A/KIAA0020 modulates PARP-1 cleavage upon genotoxic stress. Cancer Res. 2011;71(3):1126-34.
64. Budka JA, Ferris MW, Capone MJ, Hollenhorst PC. Common ELF1 deletion in prostate cancer bolsters oncogenic ETS function, inhibits senescence and promotes docetaxel resistance. Genes Cancer. 2018;9(5-6):198-214.
65. Genau HM, Huber J, Baschieri F, Akutsu M, Dötsch V, Farhan H, et al. CUL3-KBTBD6/KBTBD7 ubiquitin ligase cooperates with GABARAP proteins to spatially restrict TIAM1-RAC1 signaling. Mol Cell. 2015;57(6):995-1010.
66. Mauduit O, Brulard C, Lesluyes T, Delcroix V, Pérot G, Choublier N, et al. RCBTB1 Deletion Is Associated with Metastatic Outcome and Contributes to Docetaxel Resistance in Nontranslocation-Related Pleomorphic Sarcomas. Cancers (Basel). 2019;11(1).
67. Williams EA, Montesion M, Sharaf R, Corines J, Patel PJ, Gillespie BJ, et al. CYLD-mutant cylindroma-like basaloid carcinoma of the anus: a genetically and morphologically distinct class of HPV-related anal carcinoma. Mod Pathol. 2020.
68. Corbin MV, Rockx DA, Oostra AB, Joenje H, Dorsman JC. The iron-sulfur cluster assembly network component NARFL is a key element in the cellular defense against oxidative stress. Free Radic Biol Med. 2015;89:863-72.
69. Rao RR, Long JZ, White JP, Svensson KJ, Lou J, Lokurkar I, et al. Meteorin-like is a hormone that regulates immune-adipose interactions to increase beige fat thermogenesis. Cell. 2014;157(6):1279-91.
70. Shan L, Zhou X, Liu X, Wang Y, Su D, Hou Y, et al. FOXK2 Elicits Massive Transcription Repression and Suppresses the Hypoxic Response and Breast Cancer Carcinogenesis. Cancer Cell. 2016;30(5):708-22.
71. Pan L, Yang H, Xu C, Chen S, Meng Z, Li K, et al. ZNF750 inhibited the malignant progression of oral squamous cell carcinoma by regulating tumor vascular microenvironment. Biomed Pharmacother. 2018;105:566-72.
72. Fan L, Li H, Zhang Y. LINC00908 negatively regulates microRNA-483-5p to increase TSPYL5 expression and inhibit the development of prostate cancer. Cancer Cell Int. 2020;20:10.
73. Wu C, Su Z, Lin M, Ou J, Zhao W, Cui J, et al. NLRP11 attenuates Toll-like receptor signalling by targeting TRAF6 for degradation via the ubiquitin ligase RNF19A. Nat Commun. 2017;8(1):1977.
74. Byrne BM, Oakley GG. Replication protein A, the laxative that keeps DNA regular: The importance of RPA phosphorylation in maintaining genome stability. Semin Cell Dev Biol. 2019;86:112-20.
75. Kemp MG, Mason AC, Carreira A, Reardon JT, Haring SJ, Borgstahl GE, et al. An alternative form of replication protein a expressed in normal human tissues supports DNA repair. J Biol Chem. 2010;285(7):4788-97.
76. Fei LR, Huang WJ, Wang Y, Lei L, Li ZH, Zheng YW, et al. PRDM16 functions as a suppressor of lung adenocarcinoma metastasis. J Exp Clin Cancer Res. 2019;38(1):35.
77. Takimoto M. Multidisciplinary Roles of LRRFIP1/GCF2 in Human Biological Systems and Diseases. Cells. 2019;8(2).
78. Li HH, Cai X, Shouse GP, Piluso LG, Liu X. A specific PP2A regulatory subunit, B56gamma, mediates DNA damage-induced dephosphorylation of p53 at Thr55. Embo j. 2007;26(2):402-11.
79. Holmquist Mengelbier L, Lindell-Munther S, Yasui H, Jansson C, Esfandyari J, Karlsson J, et al. The Iroquois homeobox proteins IRX3 and IRX5 have distinct roles in Wilms tumour development and human nephrogenesis. J Pathol. 2019;247(1):86-98.
80. Schwarte-Waldhoff I, Volpert OV, Bouck NP, Sipos B, Hahn SA, Klein-Scory S, et al. Smad4/DPC4-mediated tumor suppression through suppression of angiogenesis. Proceedings of the National Academy of Sciences. 2000;97(17):9624-9.

81. Lu W, Huang SY, Su L, Zhao BX, Miao JY. Long Noncoding RNA LOC100129973 Suppresses Apoptosis by Targeting miR-4707-5p and miR-4767 in Vascular Endothelial Cells. Sci Rep. 2016;6:21620.
82. Teng W, Qiu C, He Z, Wang G, Xue Y, Hui X. Linc00152 suppresses apoptosis and promotes migration by sponging miR-4767 in vascular endothelial cells. Oncotarget. 2017;8(49):85014-23.
83. Min P, Li W, Zeng D, Ma Y, Xu D, Zheng W, et al. A single nucleotide variant in microRNA-1269a promotes the occurrence and process of hepatocellular carcinoma by targeting to oncogenes SPATS2L and LRP6. Bull Cancer. 2017;104(4):311-20.
84. Masuda M, Uno Y, Ohbayashi N, Ohata H, Mimata A, Kukimoto-Niino M, et al. TNIK inhibition abrogates colorectal cancer stemness. Nat Commun. 2016;7:12586.
85. Diaz A, Puig-Butillé JA, Muñoz C, Costa D, Díez A, Garcia-Herrera A, et al. TERT gene amplification is associated with poor outcome in acral lentiginous melanoma. J Am Acad Dermatol. 2014;71(4):839-41.
86. Miles LA, Baik N, Krajewski S, Parmer RJ, Mueller BM. The Novel Plasminogen Receptor, Plg-RKT, and Breast Cancer Progression. Blood. 2011;118(21):853-.
87. Tian R, Li X, Gao Y, Li Y, Yang P, Wang K. Identification and validation of the role of matrix metalloproteinase-1 in cervical cancer. Int J Oncol. 2018;52(4):1198-208.
88. Yan Y, Zuo X, Wei D. Concise Review: Emerging Role of CD44 in Cancer Stem Cells: A Promising Biomarker and Therapeutic Target. Stem Cells Transl Med. 2015;4(9):1033-43.
89. Liu Z, Zhang S, Hou F, Zhang C, Gao J, Wang K. Inhibition of Ca(2+) -activated chloride channel ANO1 suppresses ovarian cancer through inactivating PI3K/Akt signaling. Int J Cancer. 2019;144(9):2215-26.
90. Li Y, Wei J, Xu CH, Zhao ZX, You TG. Prognostic Significance of Cyclin D1 Expression in Colorectal Cancer: A Meta-Analysis of Observational Studies. Plos One. 2014;9(4).
91. Cai C, Rajaram M, Zhou X, Liu Q, Marchica J, Li J, et al. Activation of multiple cancer pathways and tumor maintenance function of the 3q amplified oncogene FNDC3B. Cell Cycle. 2012;11(9):1773-81.
92. Han B, Wang H, Zhang J, Tian J. FNDC3B is associated with ER stress and poor prognosis in cervical cancer. Oncol Lett. 2020;19(1):406-14.
93. Jacob A, Jing J, Lee J, Schedin P, Gilbert SM, Peden AA, et al. Rab40b regulates trafficking of MMP2 and MMP9 during invadopodia formation and invasion of breast cancer cells. J Cell Sci. 2013;126(Pt 20):4647-58.
94. Wang Y, Wu N, Sun D, Sun H, Tong D, Liu D, et al. NUBPL, a novel metastasis-related gene, promotes colorectal carcinoma cell motility by inducing epithelial-mesenchymal transition. Cancer Sci. 2017;108(6):1169-76.
95. Chen Z, Lu X, Jia D, Jing Y, Chen D, Wang Q, et al. Hepatic SMARCA4 predicts HCC recurrence and promotes tumour cell proliferation by regulating SMAD6 expression. Cell Death Dis. 2018;9(2):59.
96. Bass AJ, Watanabe H, Mermel CH, Yu S, Perner S, Verhaak RG, et al. SOX2 is an amplified lineage-survival oncogene in lung and esophageal squamous cell carcinomas. Nat Genet. 2009;41(11):1238-42.
97. Ren Q, Zhu P, Zhang H, Ye T, Liu D, Gong Z, et al. Identification and validation of stromal-tumor microenvironment-based subtypes tightly associated with PD-1/PD-L1 immunotherapy and outcomes in patients with gastric cancer. Cancer Cell Int. 2020;20:92.
98. Pan S, An P, Zhang R, He X, Yin G, Min W. Etk/Bmx as a tumor necrosis factor receptor type 2-specific kinase: role in endothelial cell migration and angiogenesis. Mol Cell Biol. 2002;22(21):7512-23.
99. Antas P, Novellasdemunt L, Kucharska A, Massie I, Carvalho J, Oukrif D, et al. SH3BP4 Regulates Intestinal Stem Cells and Tumorigenesis by Modulating β-Catenin Nuclear Localization. Cell Rep. 2019;26(9):2266-73.e4.
100. Kim YM, Stone M, Hwang TH, Kim YG, Dunlevy JR, Griffin TJ, et al. SH3BP4 is a negative regulator of amino acid-Rag GTPase-mTORC1 signaling. Mol Cell. 2012;46(6):833-46.
101. Zhang X, Ma W, Cui J, Yao H, Zhou H, Ge Y, et al. Regulation of p21 by TWIST2 contributes to its tumor-suppressor function in human acute myeloid leukemia. Oncogene. 2015;34(23):3000-10.
102. Ishikawa T, Shimizu T, Ueki A, Yamaguchi SI, Onishi N, Sugihara E, et al. Twist2 functions as a tumor suppressor in murine osteosarcoma cells. Cancer Sci. 2013;104(7):880-8.

103. Wang J, Li H, Xue B, Deng R, Huang X, Xu Y, et al. IRF1 promotes innate immune response to viral infection through enhancing the activation of IRF3. J Virol. 2020.
104. Shi Z, Fultz RS, Engevik MA, Gao C, Hall A, Major A, et al. Distinct roles of histamine H1- and H2-receptor signaling pathways in inflammation-associated colonic tumorigenesis. Am J Physiol Gastrointest Liver Physiol. 2019;316(1):G205-g16.
105. Yeste-Velasco M, Mao X, Grose R, Kudahetti SC, Lin D, Marzec J, et al. Identification of ZDHHC14 as a novel human tumour suppressor gene. J Pathol. 2014;232(5):566-77.
106. Scholl S, Popovic M, de la Rochefordiere A, Girard E, Dureau S, Mandic A, et al. Clinical and genetic landscape of treatment naive cervical cancer: Alterations in PIK3CA and in epigenetic modulators associated with sub-optimal outcome. EBioMedicine. 2019;43:253-60.
107. Kim LK, Park SA, Eoh KJ, Heo TH, Kim YT, Kim HJ. E2F8 regulates the proliferation and invasion through epithelial-mesenchymal transition in cervical cancer. Int J Biol Sci. 2020;16(2):320-9.
108. Kent LN, Rakijas JB, Pandit SK, Westendorp B, Chen HZ, Huntington JT, et al. E2f8 mediates tumor suppression in postnatal liver development. J Clin Invest. 2016;126(8):2955-69.
109. van Eggermond MC, Tezcan I, Heemskerk MH, van den Elsen PJ. Transcriptional silencing of RFXAP in MHC class II-deficiency. Mol Immunol. 2008;45(10):2920-8.
110. Lee Y, Lim B, Lee SW, Lee WR, Kim YI, Kim M, et al. ANKRD9 is associated with tumor suppression as a substrate receptor subunit of ubiquitin ligase. Biochim Biophys Acta Mol Basis Dis. 2018;1864(10):3145-53.
111. Xu R, Han Y. Long non-coding RNA FOXF1 adjacent non-coding developmental regulatory RNA inhibits growth and chemotherapy resistance in non-small cell lung cancer. Arch Med Sci. 2019;15(6):1539-46.
112. Zhang G, Wang Q, Zhang X, Ding Z, Liu R. LncRNA FENDRR suppresses the progression of NSCLC via regulating miR-761/TIMP2 axis. Biomed Pharmacother. 2019;118:109309.
113. Zhang MY, Zhang ZL, Cui HX, Wang RK, Fu L. Long non-coding RNA FENDRR inhibits NSCLC cell growth and aggressiveness by sponging miR-761. Eur Rev Med Pharmacol Sci. 2018;22(23):8324-32.
114. Liu J, Du W. LncRNA FENDRR attenuates colon cancer progression by repression of SOX4 protein. Onco Targets Ther. 2019;12:4287-95.
115. Qin X, Lu M, Zhou Y, Li G, Liu Z. LncRNA FENDRR represses proliferation, migration and invasion through suppression of survivin in cholangiocarcinoma cells. Cell Cycle. 2019;18(8):889-97.
116. Chen XF, Liu Y. MicroRNA-744 inhibited cervical cancer growth and progression through apoptosis induction by regulating Bcl-2. Biomed Pharmacother. 2016;81:379-87.
117. Wang H, Xing D, Ren D, Feng W, Chen Y, Zhao Z, et al. MicroRNA-643 regulates the expression of ZEB1 and inhibits tumorigenesis in osteosarcoma. Mol Med Rep. 2017;16(4):5157-64.
118. Zhou ZJ, Luo CB, Xin HY, Hu ZQ, Zhu GQ, Li J, et al. MACROD2 deficiency promotes hepatocellular carcinoma growth and metastasis by activating GSK-3β/β-catenin signaling. NPJ Genom Med. 2020;5:15.
119. Waly AA, El-Ekiaby N, Assal RA, Abdelrahman MM, Hosny KA, El Tayebi HM, et al. Methylation in MIRLET7A3 Gene Induces the Expression of IGF-II and Its mRNA Binding Proteins IGF2BP-2 and 3 in Hepatocellular Carcinoma. Front Physiol. 2018;9:1918.
120. Kumar V, Mondal G, Slavik P, Rachagani S, Batra SK, Mahato RI. Codelivery of small molecule hedgehog inhibitor and miRNA for treating pancreatic cancer. Mol Pharm. 2015;12(4):1289-98.
121. Wang Y, Marino-Enriquez A, Bennett RR, Zhu M, Shen Y, Eilers G, et al. Dystrophin is a tumor suppressor in human cancers with myogenic programs. Nat Genet. 2014;46(6):601-6.
122. Wang K, Mao Z, Liu L, Zhang R, Liang Q, Xiong Y, et al. Rab17 inhibits the tumourigenic properties of hepatocellular carcinomas via the Erk pathway. Tumour Biol. 2015;36(8):5815-24.
123. Chen Y, Huang Y, Huang Y, Xia X, Zhang J, Zhou Y, et al. JWA suppresses tumor angiogenesis via Sp1-activated matrix metalloproteinase-2 and its prognostic significance in human gastric cancer. Carcinogenesis. 2014;35(2):442-51.

124. Kung CP, Khaku S, Jennis M, Zhou Y, Murphy ME. Identification of TRIML2, a novel p53 target, that enhances p53 SUMOylation and regulates the transactivation of proapoptotic genes. Mol Cancer Res. 2015;13(2):250-62.
125. Jantas D, Grygier B, Gołda S, Chwastek J, Zatorska J, Tertil M. An endogenous and ectopic expression of metabotropic glutamate receptor 8 (mGluR8) inhibits proliferation and increases chemosensitivity of human neuroblastoma and glioma cells. Cancer Lett. 2018;432:1-16.
126. Wang S, Cheng Y, Zheng Y, He Z, Chen W, Zhou W, et al. PRKAR1A is a functional tumor suppressor inhibiting ERK/Snail/E-cadherin pathway in lung adenocarcinoma. Sci Rep. 2016;6:39630.
127. Khanna P, Lee JS, Sereemaspun A, Lee H, Baeg GH. GRAMD1B regulates cell migration in breast cancer cells through JAK/STAT and Akt signaling. Sci Rep. 2018;8(1):9511.
128. Marastoni S, Andreuzzi E, Paulitti A, Colladel R, Pellicani R, Todaro F, et al. EMILIN2 down-modulates the Wnt signalling pathway and suppresses breast cancer cell growth and migration. J Pathol. 2014;232(4):391-404.
129. Spiombi E, Angrisani A, Fonte S, De Feudis G, Fabretti F, Cucchi D, et al. KCTD15 inhibits the Hedgehog pathway in Medulloblastoma cells by increasing protein levels of the oncosuppressor KCASH2. Oncogenesis. 2019;8(11):64.
130. Nakamura E, Kozaki K, Tsuda H, Suzuki E, Pimkhaokham A, Yamamoto G, et al. Frequent silencing of a putative tumor suppressor gene melatonin receptor 1 A (MTNR1A) in oral squamous-cell carcinoma. Cancer Sci. 2008;99(7):1390-400.
131. Graumann R, Di Capua GA, Oyarzún JE, Vásquez MA, Liao C, Brañes JA, et al. Expression of teneurins is associated with tumor differentiation and patient survival in ovarian cancer. PLoS One. 2017;12(5):e0177244.
132. Chen D, Li Y, Wang L, Jiao K. SEMA6D Expression and Patient Survival in Breast Invasive Carcinoma. Int J Breast Cancer. 2015;2015:539721.
133. Dong X, Tang W, Stopenski S, Brose MS, Korch C, Meinkoth JL. RAP1GAP inhibits cytoskeletal remodeling and motility in thyroid cancer cells. Endocr Relat Cancer. 2012;19(4):575-88.
